# A bacterial phyla dataset for protein function prediction

**DOI:** 10.1016/j.dib.2019.105002

**Published:** 2019-12-18

**Authors:** Sarthak Mishra, Yash Pratap Rastogi, Suraiya Jabin, Punit Kaur, Mohammad Amir, Shabanam Khatoon

**Affiliations:** a Department of Computer Science, Jamia Millia Islamia, Jamia Nagar, New Delhi, 110025, Delhi, India; b Department of Biophysics, All India Institute of Medical Sciences (AIIMS), New Delhi, 110029, Delhi, India

**Keywords:** Reviewed protein, Unreviewed protein, Motif, Physicochemical features, Sequence-based features, Annotation based features, Function prediction, Molecular function

## Abstract

Protein function prediction has been the most worked upon and the most challenging problem for computational biologists. The vast majority of known proteins have yet not been characterised experimentally, and there is significant gap between their structures and functions. New un-annotated sequences are being added to the public protein databases (e.g. UniprotKB) at an enormous pace [1]. Such proteins with unknown functions might play key role in the metabolism, growth and development regulation. Thus, if functions of unknown proteins left undiscovered, researchers may skip important information(s). Based on their sequence, structure, evolutionary history, and their association with other proteins, tools of computational biology can provide insights into the function of proteins [2]. For proteins with well characterised close relatives, it is trivial to infer function. Orphan proteins without discernible sequence relatives present a greater challenge [3]. Here the task of experimental characterisation is blind and becomes unwieldy. It is highly unlikely that all known proteins will ever be completely experimentally characterised [4]. Thus, there is an emergent need to develop fast and accurate computational approaches to fulfil this requirement. Towards this end, we prepared a dataset for protein function prediction by extracting protein sequences and annotations of reviewed prokaryotic proteins (total count 323,719 as accessed on date March 10, 2019) belonging to 9 bacterial phyla Actinobacteria, Bacteroidetes, Chlamydiae, Cyanobacteria, Firmicutes, Fusobacteria, Proteobacteria, Spirochaetes and Tenericutes. Corresponding to the most frequent 1739 Gene Ontology (Molecular Function) terms, samples were filtered, and 171,212 proteins were retrieved for feature generation. The Dataset was generated by calculating the sequence, sub-sequence, physiochemical, annotation-based features for each 171,212 reviewed proteins using method in [10].

These features constitute a total of 9890 attributes for each sequence of protein along with 1739 Gene Ontology terms. Each protein sequence is assigned one or more of 1739 Gene Ontology (Molecular Function) term as its target label. The Dataset contains the Entry and Entry name of each sequence corresponding to UniprotKB Database. This dataset being huge in size (171,212 samples X 9890 features, 1739 classes with multiple values) and equipped with enough number of positive and negative samples of each 1739 class, is good for testing efficiency of any upcoming deep learning models [5]. We divided the full dataset of 171,212 reviewed proteins in the ratio 3:1 to form Train/Test dataset 1; train dataset with 128,409 samples and test dataset with 42,803 samples to facilitate training of a deep learning model. The train and test datasets are stratified to contain good proportion of each 1739 classes. We then prepared a dataset 2 of pathogenic unreviewed proteins of the 9 bacterial phyla each with 9890 features same as train/train dataset of reviewed proteins but without target labels in order to predict their functions using deep learning model proposed in [5].

**Table:** Specifications Table

Subject	Biochemistry, Genetics and Molecular Biology (General)
Specific subject area	Deep learning task for protein function prediction of 9 bacterial phyla into multi-valued and multi-class labels
Type of data	Tables (excel sheets) and Fasta files
How data were acquired	Web-Scraping and Feature Generation through Python libraries
Data format	**Raw:**
	- Fasta Sequences of 171,212 proteins of 9 bacterial phyla
	**Analysed and Filtered:**
	- Train/Test Dataset 1 with 9890 extracted features and 1739 GO terms in the form of Training vectors for 171,212 proteins of 9 bacterial phyla
	- Test Dataset 2 with 9890 extracted features for unreviewed protein of the 9 phyla extracted from UniProtKB for predictions using deep neural network based protein function prediction model [5].
Parameters for data collection	Both Reviewed and Unreviewed protein sequences were collected from UniprotKB belonging to 9 bacterial Phyla. Reviewed Proteins were used to generate Dataset for Training and Testing (Train/Test Dataset 1). Unreviewed Proteins (with annotation score 1 or 2 out of 5, and proteins with evidence of existence level either predicted or uncertain) from UniprotKB belonging to 9 bacterial Phyla were used to generate Dataset for predictions only (Test Dataset 2).
Description of data collection	Data was collected using Python Web-Scraping library from UniprotKB and Prosite Servers. The 323,719 reviewed protein Sequences were downloaded from UniprotKB and their Motifs were extracted from the Prosite Server. The Sequences were then filtered using relevant 1739 Gene Ontology (Molecular Function domain). The sequence, subsequence (motif count), annotation, and physiochemical features for filtered 171,212 protein sequences were generated using method in [10]. The final Dataset contains Entry, Entry name, Sequences, 9890 generated features and 1739 GO terms for each sample.
Data source location	https://www.uniprot.org/(for downloading reviewed and unreviewed protein sequences of 9 bacterial Phyla) https://prosite.expasy.org/(for acquiring motifs of protein sequences of 9 bacterial phyla)
Data accessibility	With the article As well as in a public repository: Repository name: GitHub URL: https://github.com/sarry2905/Protein-Function-Prediction
Related research article	Author's name: Sarthak Mishra, Yash Pratap Rastogi, Suraiya Jabin, Punit Kaur, Mohammad Amir, Shabanam Khatoon Title: A deep neural network based model for function prediction of hypothetical proteins from pathogenic bacterial species [5] Journal: Computational Biology and Chemistry (under review) DOI: (under progress)

**Table:** 

**Value of the Data**This dataset can be used for training a machine learning based model for probable function prediction of proteins belonging to the considered bacterial phyla without functional annotation i.e. under category unreviewed (TrEMBL)-computationally analysed on UniProtKB. • This dataset contributes important step towards the protein function prediction problem for bacterial species. • Researchers trying to design new deep learning models can use this dataset for testing performance of their model. • We provide 1739 molecular function domain GO terms as target label in the dataset for designing a supervised learning model but these 1739 GO terms can be used as features as well for some other kind of study such as clustering of bacterial proteins into functional groups etc. • This dataset being huge in size, can be used to test and design GPU based parallelized deep learning algorithms for multi-class labelling.

## 1 Data

The 171,212 extracted reviewed protein samples belong to 9 bacterial phyla Actinobacteria, Bacteroidetes, Chlamydiae, Cyanobacteria, Firmicutes, Fusobacteria, Proteobacteria, Spirochaetes and Tenericutes. Each Phyla has a Train and Test.csv (comma separated values) files, where Train file contains the 75% of data and Test file contains 25% of the data from each Phyla. A Test dataset 2 was constructed for pathogenic unreviewed protein sequences belonging to 9 bacterial Phyla. These entries in UniProtKB have yet not received any annotation [[1], [2], [3], [4]] towards Gene Ontology and therefore can be used for prediction.

Each data file contains the following columns given below in points 1 to 8.

1. Entry

Entry is the unique ID given to each protein entry available on UniProtKB.

2. Entry name

Entry name is a mnemonic identifier for the unique ID provided to each protein entry.

3. Sequence

Amino acid sequence for the corresponding protein entry.

4. Sequence based Features

These are the attributes guided by the primary structure of protein.

5. Physicochemical Features

These are the attributes based on the physical and chemical properties of the monomeric unit of a protein i.e. an amino acid.

6. Annotation based features

These are the attributes based on already present annotations regarding subcellular localisation, binding preference of proteins and presence of transmembrane regions.

7. Subsequence based features

These are the attributes corresponding to the local similarities within a given protein sequence.

8. Gene Ontology (Molecular Function domain only) terms

The following are the names of supplementary data files along with their short description:

**Dataset 1 (FASTA files of Dataset 1):** Fasta Sequences of 171,212 proteins of 9 bacterial phyla in 2 parts with names “Dataset1 non-proteo.fasta” (containing fasta sequences of all proteins of phylum other than proteobacterium) and “Dataset1 proteo.fasta” (containing fasta sequences of all proteins of phylum proteobacterium).

These two fasta files are zipped together *(fasta seq of dataset.zip).*

**Dataset 2 (Train Dataset 1):** with feature vectors extracted from reviewed proteins (75% of 171,212 reviewed proteins) of 9 Bacterial phyla. A total of 18 excel sheets all zipped, also available on project's GitHub repository.

**Dataset 3 (Test Dataset 1):** with feature vectors extracted from reviewed proteins (25% of 171,212 reviewed proteins) 9 Bacterial phyla. A total of 12 excel sheets all zipped, also available on project's GitHub repository.

**Dataset 4 (Test Dataset 2):** with feature vectors extracted from unreviewed and hypothetical Proteins of 9 Bacterial phyla from pathogenic bacterial species (9 excel sheets all zipped).

**Dataset 5:** Predictions on Test Dataset 2 (9 excel sheets all zipped).

## 2 Experiment design, materials, and methods

Using web-scraping libraries in Python [7], reviewed proteins of 9 bacterial phyla were extracted from UniprotKB. These samples were filtered based on the relevant 1739 Gene Ontology (belonging to molecular function domain only) terms. Further, for each sample, Motifs were extracted from Prosite server [9] using Python. These Motifs were analysed to remove redundancy and added as feature in dataset. Finally, for each sample, Sequence-based, sub-sequence-based [8], annotation-based and Physiochemical features were calculated along with Gene Ontology (Molecular Function) as a target label (If a sample consist a GO term, it had 1 in the corresponding column, else 0). All the features are generated using method in [10] utilising the following packages: Biopython [7], and I-feature [6]. The dataset acquired is then randomly split into two parts: Train (75%) and Test (25%) for each phylum, each of which is stratified to contain good proportion of each 1739 classes.

Every Train/Test dataset which is part of this bacterial phyla dataset for protein function prediction is having 9890 features and 1739 GO terms stored in excel (CSV) sheet format. Test dataset 2 is not having any target label associated with its entries as this dataset is used for predictions and belongs to hypothetical and unreviewed category.

A complete list of these 9890 features extracted for training/testing is shown below and summarised in Table 1 [5]: (Note: Entry, Entry name and Sequence may not be used for training but they are present in each csv file).

1. Sequence based features (Count of Amino acid compositions, Dipeptide composition, Tripeptide composition) of protein sequences with a minimum length of 30 amino acids.
  A,C,D,E,F,G,H,I,K,L,M,N,P,Q,R,S,T,V,W,Y,AA,AC,AD,AE,AF,AG,AH,AI,AK,AL,AM,AN,AP,AQ,AR,AS,AT,AV,AW,AY,CA,CC,CD,CE,CF,CG,CH,CI,CK,CL,CM,CN,CP,CQ,CR,CS,CT,CV,CW,CY,DA,DC,DD,DE,DF,DG,DH,DI,DK,DL,DM,DN,DP,DQ,DR,DS,DT,DV,DW,DY,EA,EC,ED,EE,EF,EG,EH,EI,EK,EL,EM,EN,EP,EQ,ER,ES,ET,EV,EW,EY,FA,FC,FD,FE,FF,FG,FH,FI,FK,FL,FM,FN,FP,FQ,FR,FS,FT,FV,FW,FY,GA,GC,GD,GE,GF,GG,GH,GI,GK,GL,GM,GN,GP,GQ,GR,GS,GT,GV,GW,GY,HA,HC,HD,HE,HF,HG,HH,HI,HK,HL,HM,HN,HP,HQ,HR,HS,HT,HV,HW,HY,IA,IC,ID,IE,IF,IG,IH,II,IK,IL,IM,IN,IP,IQ,IR,IS,IT,IV,IW,IY,KA,KC,KD,KE,KF,KG,KH,KI,KK,KL,KM,KN,KP,KQ,KR,KS,KT,KV,KW,KY,LA,LC,LD,LE,LF,LG,LH,LI,LK,LL,LM,LN,LP,LQ,LR,LS,LT,LV,LW,LY,MA,MC,MD,ME,MF,MG,MH,MI,MK,ML,MM,MN,MP,MQ,MR,MS,MT,MV,MW,MY,NA,NC,ND,NE,NF,NG,NH,NI,NK,NL,NM,NN,NP,NQ,NR,NS,NT,NV,NW,NY,PA,PC,PD,PE,PF,PG,PH,PI,PK,PL,PM,PN,PP,PQ,PR,PS,PT,PV,PW,PY,QA,QC,QD,QE,QF,QG,QH,QI,QK,QL,QM,QN,QP,QQ,QR,QS,QT,QV,QW,QY,RA,RC,RD,RE,RF,RG,RH,RI,RK,RL,RM,RN,RP,RQ,RR,RS,RT,RV,RW,RY,SA,SC,SD,SE,SF,SG,SH,SI,SK,SL,SM,SN,SP,SQ,SR,SS,ST,SV,SW,SY,TA,TC,TD,TE,TF,TG,TH,TI,TK,TL,TM,TN,TP,TQ,TR,TS,TT,TV,TW,TY,VA,VC,VD,VE,VF,VG,VH,VI,VK,VL,VM,VN,VP,VQ,VR,VS,VT,VV,VW,VY,WA,WC,WD,WE,WF,WG,WH,WI,WK,WL,WM,WN,WP,WQ,WR,WS,WT,WV,WW,WY,YA,YC,YD,YE,YF,YG,YH,YI,YK,YL,YM,YN,YP,YQ,YR,YS,YT,YV,YW,YY,AAA,AAC,AAD,AAE,AAF,AAG,AAH,AAI,AAK,AAL,AAM,AAN,AAP,AAQ,AAR,AAS,AAT,AAV,AAW,AAY,ACA,ACC,ACD,ACE,ACF,ACG,ACH,ACI,ACK,ACL,ACM,ACN,ACP,ACQ,ACR,ACS,ACT,ACV,ACW,ACY,ADA,ADC,ADD,ADE,ADF,ADG,ADH,ADI,ADK,ADL,ADM,ADN,ADP,ADQ,ADR,ADS,ADT,ADV,ADW,ADY,AEA,AEC,AED,AEE,AEF,AEG,AEH,AEI,AEK,AEL,AEM,AEN,AEP,AEQ,AER,AES,AET,AEV,AEW,AEY,AFA,AFC,AFD,AFE,AFF,AFG,AFH,AFI,AFK,AFL,AFM,AFN,AFP,AFQ,AFR,AFS,AFT,AFV,AFW,AFY,AGA,AGC,AGD,AGE,AGF,AGG,AGH,AGI,AGK,AGL,AGM,AGN,AGP,AGQ,AGR,AGS,AGT,AGV,AGW,AGY,AHA,AHC,AHD,AHE,AHF,AHG,AHH,AHI,AHK,AHL,AHM,AHN,AHP,AHQ,AHR,AHS,AHT,AHV,AHW,AHY,AIA,AIC,AID,AIE,AIF,AIG,AIH,AII,AIK,AIL,AIM,AIN,AIP,AIQ,AIR,AIS,AIT,AIV,AIW,AIY,AKA,AKC,AKD,AKE,AKF,AKG,AKH,AKI,AKK,AKL,AKM,AKN,AKP,AKQ,AKR,AKS,AKT,AKV,AKW,AKY,ALA,ALC,ALD,ALE,ALF,ALG,ALH,ALI,ALK,ALL,ALM,ALN,ALP,ALQ,ALR,ALS,ALT,ALV,ALW,ALY,AMA,AMC,AMD,AME,AMF,AMG,AMH,AMI,AMK,AML,AMM,AMN,AMP,AMQ,AMR,AMS,AMT,AMV,AMW,AMY,ANA,ANC,AND,ANE,ANF,ANG,ANH,ANI,ANK,ANL,ANM,ANN,ANP,ANQ,ANR,ANS,ANT,ANV,ANW,ANY,APA,APC,APD,APE,APF,APG,APH,API,APK,APL,APM,APN,APP,APQ,APR,APS,APT,APV,APW,APY,AQA,AQC,AQD,AQE,AQF,AQG,AQH,AQI,AQK,AQL,AQM,AQN,AQP,AQQ,AQR,AQS,AQT,AQV,AQW,AQY,ARA,ARC,ARD,ARE,ARF,ARG,ARH,ARI,ARK,ARL,ARM,ARN,ARP,ARQ,ARR,ARS,ART,ARV,ARW,ARY,ASA,ASC,ASD,ASE,ASF,ASG,ASH,ASI,ASK,ASL,ASM,ASN,ASP,ASQ,ASR,ASS,AST,ASV,ASW,ASY,ATA,ATC,ATD,ATE,ATF,ATG,ATH,ATI,ATK,ATL,ATM,ATN,ATP,ATQ,ATR,ATS,ATT,ATV,ATW,ATY,AVA,AVC,AVD,AVE,AVF,AVG,AVH,AVI,AVK,AVL,AVM,AVN,AVP,AVQ,AVR,AVS,AVT,AVV,AVW,AVY,AWA,AWC,AWD,AWE,AWF,AWG,AWH,AWI,AWK,AWL,AWM,AWN,AWP,AWQ,AWR,AWS,AWT,AWV,AWW,AWY,AYA,AYC,AYD,AYE,AYF,AYG,AYH,AYI,AYK,AYL,AYM,AYN,AYP,AYQ,AYR,AYS,AYT,AYV,AYW,AYY,CAA,CAC,CAD,CAE,CAF,CAG,CAH,CAI,CAK,CAL,CAM,CAN,CAP,CAQ,CAR,CAS,CAT,CAV,CAW,CAY,CCA,CCC,CCD,CCE,CCF,CCG,CCH,CCI,CCK,CCL,CCM,CCN,CCP,CCQ,CCR,CCS,CCT,CCV,CCW,CCY,CDA,CDC,CDD,CDE,CDF,CDG,CDH,CDI,CDK,CDL,CDM,CDN,CDP,CDQ,CDR,CDS,CDT,CDV,CDW,CDY,CEA,CEC,CED,CEE,CEF,CEG,CEH,CEI,CEK,CEL,CEM,CEN,CEP,CEQ,CER,CES,CET,CEV,CEW,CEY,CFA,CFC,CFD,CFE,CFF,CFG,CFH,CFI,CFK,CFL,CFM,CFN,CFP,CFQ,CFR,CFS,CFT,CFV,CFW,CFY,CGA,CGC,CGD,CGE,CGF,CGG,CGH,CGI,CGK,CGL,CGM,CGN,CGP,CGQ,CGR,CGS,CGT,CGV,CGW,CGY,CHA,CHC,CHD,CHE,CHF,CHG,CHH,CHI,CHK,CHL,CHM,CHN,CHP,CHQ,CHR,CHS,CHT,CHV,CHW,CHY,CIA,CIC,CID,CIE,CIF,CIG,CIH,CII,CIK,CIL,CIM,CIN,CIP,CIQ,CIR,CIS,CIT,CIV,CIW,CIY,CKA,CKC,CKD,CKE,CKF,CKG,CKH,CKI,CKK,CKL,CKM,CKN,CKP,CKQ,CKR,CKS,CKT,CKV,CKW,CKY,CLA,CLC,CLD,CLE,CLF,CLG,CLH,CLI,CLK,CLL,CLM,CLN,CLP,CLQ,CLR,CLS,CLT,CLV,CLW,CLY,CMA,CMC,CMD,CME,CMF,CMG,CMH,CMI,CMK,CML,CMM,CMN,CMP,CMQ,CMR,CMS,CMT,CMV,CMW,CMY,CNA,CNC,CND,CNE,CNF,CNG,CNH,CNI,CNK,CNL,CNM,CNN,CNP,CNQ,CNR,CNS,CNT,CNV,CNW,CNY,CPA,CPC,CPD,CPE,CPF,CPG,CPH,CPI,CPK,CPL,CPM,CPN,CPP,CPQ,CPR,CPS,CPT,CPV,CPW,CPY,CQA,CQC,CQD,CQE,CQF,CQG,CQH,CQI,CQK,CQL,CQM,CQN,CQP,CQQ,CQR,CQS,CQT,CQV,CQW,CQY,CRA,CRC,CRD,CRE,CRF,CRG,CRH,CRI,CRK,CRL,CRM,CRN,CRP,CRQ,CRR,CRS,CRT,CRV,CRW,CRY,CSA,CSC,CSD,CSE,CSF,CSG,CSH,CSI,CSK,CSL,CSM,CSN,CSP,CSQ,CSR,CSS,CST,CSV,CSW,CSY,CTA,CTC,CTD,CTE,CTF,CTG,CTH,CTI,CTK,CTL,CTM,CTN,CTP,CTQ,CTR,CTS,CTT,CTV,CTW,CTY,CVA,CVC,CVD,CVE,CVF,CVG,CVH,CVI,CVK,CVL,CVM,CVN,CVP,CVQ,CVR,CVS,CVT,CVV,CVW,CVY,CWA,CWC,CWD,CWE,CWF,CWG,CWH,CWI,CWK,CWL,CWM,CWN,CWP,CWQ,CWR,CWS,CWT,CWV,CWW,CWY,CYA,CYC,CYD,CYE,CYF,CYG,CYH,CYI,CYK,CYL,CYM,CYN,CYP,CYQ,CYR,CYS,CYT,CYV,CYW,CYY,DAA,DAC,DAD,DAE,DAF,DAG,DAH,DAI,DAK,DAL,DAM,DAN,DAP,DAQ,DAR,DAS,DAT,DAV,DAW,DAY,DCA,DCC,DCD,DCE,DCF,DCG,DCH,DCI,DCK,DCL,DCM,DCN,DCP,DCQ,DCR,DCS,DCT,DCV,DCW,DCY,DDA,DDC,DDD,DDE,DDF,DDG,DDH,DDI,DDK,DDL,DDM,DDN,DDP,DDQ,DDR,DDS,DDT,DDV,DDW,DDY,DEA,DEC,DED,DEE,DEF,DEG,DEH,DEI,DEK,DEL,DEM,DEN,DEP,DEQ,DER,DES,DET,DEV,DEW,DEY,DFA,DFC,DFD,DFE,DFF,DFG,DFH,DFI,DFK,DFL,DFM,DFN,DFP,DFQ,DFR,DFS,DFT,DFV,DFW,DFY,DGA,DGC,DGD,DGE,DGF,DGG,DGH,DGI,DGK,DGL,DGM,DGN,DGP,DGQ,DGR,DGS,DGT,DGV,DGW,DGY,DHA,DHC,DHD,DHE,DHF,DHG,DHH,DHI,DHK,DHL,DHM,DHN,DHP,DHQ,DHR,DHS,DHT,DHV,DHW,DHY,DIA,DIC,DID,DIE,DIF,DIG,DIH,DII,DIK,DIL,DIM,DIN,DIP,DIQ,DIR,DIS,DIT,DIV,DIW,DIY,DKA,DKC,DKD,DKE,DKF,DKG,DKH,DKI,DKK,DKL,DKM,DKN,DKP,DKQ,DKR,DKS,DKT,DKV,DKW,DKY,DLA,DLC,DLD,DLE,DLF,DLG,DLH,DLI,DLK,DLL,DLM,DLN,DLP,DLQ,DLR,DLS,DLT,DLV,DLW,DLY,DMA,DMC,DMD,DME,DMF,DMG,DMH,DMI,DMK,DML,DMM,DMN,DMP,DMQ,DMR,DMS,DMT,DMV,DMW,DMY,DNA,DNC,DND,DNE,DNF,DNG,DNH,DNI,DNK,DNL,DNM,DNN,DNP,DNQ,DNR,DNS,DNT,DNV,DNW,DNY,DPA,DPC,DPD,DPE,DPF,DPG,DPH,DPI,DPK,DPL,DPM,DPN,DPP,DPQ,DPR,DPS,DPT,DPV,DPW,DPY,DQA,DQC,DQD,DQE,DQF,DQG,DQH,DQI,DQK,DQL,DQM,DQN,DQP,DQQ,DQR,DQS,DQT,DQV,DQW,DQY,DRA,DRC,DRD,DRE,DRF,DRG,DRH,DRI,DRK,DRL,DRM,DRN,DRP,DRQ,DRR,DRS,DRT,DRV,DRW,DRY,DSA,DSC,DSD,DSE,DSF,DSG,DSH,DSI,DSK,DSL,DSM,DSN,DSP,DSQ,DSR,DSS,DST,DSV,DSW,DSY,DTA,DTC,DTD,DTE,DTF,DTG,DTH,DTI,DTK,DTL,DTM,DTN,DTP,DTQ,DTR,DTS,DTT,DTV,DTW,DTY,DVA,DVC,DVD,DVE,DVF,DVG,DVH,DVI,DVK,DVL,DVM,DVN,DVP,DVQ,DVR,DVS,DVT,DVV,DVW,DVY,DWA,DWC,DWD,DWE,DWF,DWG,DWH,DWI,DWK,DWL,DWM,DWN,DWP,DWQ,DWR,DWS,DWT,DWV,DWW,DWY,DYA,DYC,DYD,DYE,DYF,DYG,DYH,DYI,DYK,DYL,DYM,DYN,DYP,DYQ,DYR,DYS,DYT,DYV,DYW,DYY,EAA,EAC,EAD,EAE,EAF,EAG,EAH,EAI,EAK,EAL,EAM,EAN,EAP,EAQ,EAR,EAS,EAT,EAV,EAW,EAY,ECA,ECC,ECD,ECE,ECF,ECG,ECH,ECI,ECK,ECL,ECM,ECN,ECP,ECQ,ECR,ECS,ECT,ECV,ECW,ECY,EDA,EDC,EDD,EDE,EDF,EDG,EDH,EDI,EDK,EDL,EDM,EDN,EDP,EDQ,EDR,EDS,EDT,EDV,EDW,EDY,EEA,EEC,EED,EEE,EEF,EEG,EEH,EEI,EEK,EEL,EEM,EEN,EEP,EEQ,EER,EES,EET,EEV,EEW,EEY,EFA,EFC,EFD,EFE,EFF,EFG,EFH,EFI,EFK,EFL,EFM,EFN,EFP,EFQ,EFR,EFS,EFT,EFV,EFW,EFY,EGA,EGC,EGD,EGE,EGF,EGG,EGH,EGI,EGK,EGL,EGM,EGN,EGP,EGQ,EGR,EGS,EGT,EGV,EGW,EGY,EHA,EHC,EHD,EHE,EHF,EHG,EHH,EHI,EHK,EHL,EHM,EHN,EHP,EHQ,EHR,EHS,EHT,EHV,EHW,EHY,EIA,EIC,EID,EIE,EIF,EIG,EIH,EII,EIK,EIL,EIM,EIN,EIP,EIQ,EIR,EIS,EIT,EIV,EIW,EIY,EKA,EKC,EKD,EKE,EKF,EKG,EKH,EKI,EKK,EKL,EKM,EKN,EKP,EKQ,EKR,EKS,EKT,EKV,EKW,EKY,ELA,ELC,ELD,ELE,ELF,ELG,ELH,ELI,ELK,ELL,ELM,ELN,ELP,ELQ,ELR,ELS,ELT,ELV,ELW,ELY,EMA,EMC,EMD,EME,EMF,EMG,EMH,EMI,EMK,EML,EMM,EMN,EMP,EMQ,EMR,EMS,EMT,EMV,EMW,EMY,ENA,ENC,END,ENE,ENF,ENG,ENH,ENI,ENK,ENL,ENM,ENN,ENP,ENQ,ENR,ENS,ENT,ENV,ENW,ENY,EPA,EPC,EPD,EPE,EPF,EPG,EPH,EPI,EPK,EPL,EPM,EPN,EPP,EPQ,EPR,EPS,EPT,EPV,EPW,EPY,EQA,EQC,EQD,EQE,EQF,EQG,EQH,EQI,EQK,EQL,EQM,EQN,EQP,EQQ,EQR,EQS,EQT,EQV,EQW,EQY,ERA,ERC,ERD,ERE,ERF,ERG,ERH,ERI,ERK,ERL,ERM,ERN,ERP,ERQ,ERR,ERS,ERT,ERV,ERW,ERY,ESA,ESC,ESD,ESE,ESF,ESG,ESH,ESI,ESK,ESL,ESM,ESN,ESP,ESQ,ESR,ESS,EST,ESV,ESW,ESY,ETA,ETC,ETD,ETE,ETF,ETG,ETH,ETI,ETK,ETL,ETM,ETN,ETP,ETQ,ETR,ETS,ETT,ETV,ETW,ETY,EVA,EVC,EVD,EVE,EVF,EVG,EVH,EVI,EVK,EVL,EVM,EVN,EVP,EVQ,EVR,EVS,EVT,EVV,EVW,EVY,EWA,EWC,EWD,EWE,EWF,EWG,EWH,EWI,EWK,EWL,EWM,EWN,EWP,EWQ,EWR,EWS,EWT,EWV,EWW,EWY,EYA,EYC,EYD,EYE,EYF,EYG,EYH,EYI,EYK,EYL,EYM,EYN,EYP,EYQ,EYR,EYS,EYT,EYV,EYW,EYY,FAA,FAC,FAD,FAE,FAF,FAG,FAH,FAI,FAK,FAL,FAM,FAN,FAP,FAQ,FAR,FAS,FAT,FAV,FAW,FAY,FCA,FCC,FCD,FCE,FCF,FCG,FCH,FCI,FCK,FCL,FCM,FCN,FCP,FCQ,FCR,FCS,FCT,FCV,FCW,FCY,FDA,FDC,FDD,FDE,FDF,FDG,FDH,FDI,FDK,FDL,FDM,FDN,FDP,FDQ,FDR,FDS,FDT,FDV,FDW,FDY,FEA,FEC,FED,FEE,FEF,FEG,FEH,FEI,FEK,FEL,FEM,FEN,FEP,FEQ,FER,FES,FET,FEV,FEW,FEY,FFA,FFC,FFD,FFE,FFF,FFG,FFH,FFI,FFK,FFL,FFM,FFN,FFP,FFQ,FFR,FFS,FFT,FFV,FFW,FFY,FGA,FGC,FGD,FGE,FGF,FGG,FGH,FGI,FGK,FGL,FGM,FGN,FGP,FGQ,FGR,FGS,FGT,FGV,FGW,FGY,FHA,FHC,FHD,FHE,FHF,FHG,FHH,FHI,FHK,FHL,FHM,FHN,FHP,FHQ,FHR,FHS,FHT,FHV,FHW,FHY,FIA,FIC,FID,FIE,FIF,FIG,FIH,FII,FIK,FIL,FIM,FIN,FIP,FIQ,FIR,FIS,FIT,FIV,FIW,FIY,FKA,FKC,FKD,FKE,FKF,FKG,FKH,FKI,FKK,FKL,FKM,FKN,FKP,FKQ,FKR,FKS,FKT,FKV,FKW,FKY,FLA,FLC,FLD,FLE,FLF,FLG,FLH,FLI,FLK,FLL,FLM,FLN,FLP,FLQ,FLR,FLS,FLT,FLV,FLW,FLY,FMA,FMC,FMD,FME,FMF,FMG,FMH,FMI,FMK,FML,FMM,FMN,FMP,FMQ,FMR,FMS,FMT,FMV,FMW,FMY,FNA,FNC,FND,FNE,FNF,FNG,FNH,FNI,FNK,FNL,FNM,FNN,FNP,FNQ,FNR,FNS,FNT,FNV,FNW,FNY,FPA,FPC,FPD,FPE,FPF,FPG,FPH,FPI,FPK,FPL,FPM,FPN,FPP,FPQ,FPR,FPS,FPT,FPV,FPW,FPY,FQA,FQC,FQD,FQE,FQF,FQG,FQH,FQI,FQK,FQL,FQM,FQN,FQP,FQQ,FQR,FQS,FQT,FQV,FQW,FQY,FRA,FRC,FRD,FRE,FRF,FRG,FRH,FRI,FRK,FRL,FRM,FRN,FRP,FRQ,FRR,FRS,FRT,FRV,FRW,FRY,FSA,FSC,FSD,FSE,FSF,FSG,FSH,FSI,FSK,FSL,FSM,FSN,FSP,FSQ,FSR,FSS,FST,FSV,FSW,FSY,FTA,FTC,FTD,FTE,FTF,FTG,FTH,FTI,FTK,FTL,FTM,FTN,FTP,FTQ,FTR,FTS,FTT,FTV,FTW,FTY,FVA,FVC,FVD,FVE,FVF,FVG,FVH,FVI,FVK,FVL,FVM,FVN,FVP,FVQ,FVR,FVS,FVT,FVV,FVW,FVY,FWA,FWC,FWD,FWE,FWF,FWG,FWH,FWI,FWK,FWL,FWM,FWN,FWP,FWQ,FWR,FWS,FWT,FWV,FWW,FWY,FYA,FYC,FYD,FYE,FYF,FYG,FYH,FYI,FYK,FYL,FYM,FYN,FYP,FYQ,FYR,FYS,FYT,FYV,FYW,FYY,GAA,GAC,GAD,GAE,GAF,GAG,GAH,GAI,GAK,GAL,GAM,GAN,GAP,GAQ,GAR,GAS,GAT,GAV,GAW,GAY,GCA,GCC,GCD,GCE,GCF,GCG,GCH,GCI,GCK,GCL,GCM,GCN,GCP,GCQ,GCR,GCS,GCT,GCV,GCW,GCY,GDA,GDC,GDD,GDE,GDF,GDG,GDH,GDI,GDK,GDL,GDM,GDN,GDP,GDQ,GDR,GDS,GDT,GDV,GDW,GDY,GEA,GEC,GED,GEE,GEF,GEG,GEH,GEI,GEK,GEL,GEM,GEN,GEP,GEQ,GER,GES,GET,GEV,GEW,GEY,GFA,GFC,GFD,GFE,GFF,GFG,GFH,GFI,GFK,GFL,GFM,GFN,GFP,GFQ,GFR,GFS,GFT,GFV,GFW,GFY,GGA,GGC,GGD,GGE,GGF,GGG,GGH,GGI,GGK,GGL,GGM,GGN,GGP,GGQ,GGR,GGS,GGT,GGV,GGW,GGY,GHA,GHC,GHD,GHE,GHF,GHG,GHH,GHI,GHK,GHL,GHM,GHN,GHP,GHQ,GHR,GHS,GHT,GHV,GHW,GHY,GIA,GIC,GID,GIE,GIF,GIG,GIH,GII,GIK,GIL,GIM,GIN,GIP,GIQ,GIR,GIS,GIT,GIV,GIW,GIY,GKA,GKC,GKD,GKE,GKF,GKG,GKH,GKI,GKK,GKL,GKM,GKN,GKP,GKQ,GKR,GKS,GKT,GKV,GKW,GKY,GLA,GLC,GLD,GLE,GLF,GLG,GLH,GLI,GLK,GLL,GLM,GLN,GLP,GLQ,GLR,GLS,GLT,GLV,GLW,GLY,GMA,GMC,GMD,GME,GMF,GMG,GMH,GMI,GMK,GML,GMM,GMN,GMP,GMQ,GMR,GMS,GMT,GMV,GMW,GMY,GNA,GNC,GND,GNE,GNF,GNG,GNH,GNI,GNK,GNL,GNM,GNN,GNP,GNQ,GNR,GNS,GNT,GNV,GNW,GNY,GPA,GPC,GPD,GPE,GPF,GPG,GPH,GPI,GPK,GPL,GPM,GPN,GPP,GPQ,GPR,GPS,GPT,GPV,GPW,GPY,GQA,GQC,GQD,GQE,GQF,GQG,GQH,GQI,GQK,GQL,GQM,GQN,GQP,GQQ,GQR,GQS,GQT,GQV,GQW,GQY,GRA,GRC,GRD,GRE,GRF,GRG,GRH,GRI,GRK,GRL,GRM,GRN,GRP,GRQ,GRR,GRS,GRT,GRV,GRW,GRY,GSA,GSC,GSD,GSE,GSF,GSG,GSH,GSI,GSK,GSL,GSM,GSN,GSP,GSQ,GSR,GSS,GST,GSV,GSW,GSY,GTA,GTC,GTD,GTE,GTF,GTG,GTH,GTI,GTK,GTL,GTM,GTN,GTP,GTQ,GTR,GTS,GTT,GTV,GTW,GTY,GVA,GVC,GVD,GVE,GVF,GVG,GVH,GVI,GVK,GVL,GVM,GVN,GVP,GVQ,GVR,GVS,GVT,GVV,GVW,GVY,GWA,GWC,GWD,GWE,GWF,GWG,GWH,GWI,GWK,GWL,GWM,GWN,GWP,GWQ,GWR,GWS,GWT,GWV,GWW,GWY,GYA,GYC,GYD,GYE,GYF,GYG,GYH,GYI,GYK,GYL,GYM,GYN,GYP,GYQ,GYR,GYS,GYT,GYV,GYW,GYY,HAA,HAC,HAD,HAE,HAF,HAG,HAH,HAI,HAK,HAL,HAM,HAN,HAP,HAQ,HAR,HAS,HAT,HAV,HAW,HAY,HCA,HCC,HCD,HCE,HCF,HCG,HCH,HCI,HCK,HCL,HCM,HCN,HCP,HCQ,HCR,HCS,HCT,HCV,HCW,HCY,HDA,HDC,HDD,HDE,HDF,HDG,HDH,HDI,HDK,HDL,HDM,HDN,HDP,HDQ,HDR,HDS,HDT,HDV,HDW,HDY,HEA,HEC,HED,HEE,HEF,HEG,HEH,HEI,HEK,HEL,HEM,HEN,HEP,HEQ,HER,HES,HET,HEV,HEW,HEY,HFA,HFC,HFD,HFE,HFF,HFG,HFH,HFI,HFK,HFL,HFM,HFN,HFP,HFQ,HFR,HFS,HFT,HFV,HFW,HFY,HGA,HGC,HGD,HGE,HGF,HGG,HGH,HGI,HGK,HGL,HGM,HGN,HGP,HGQ,HGR,HGS,HGT,HGV,HGW,HGY,HHA,HHC,HHD,HHE,HHF,HHG,HHH,HHI,HHK,HHL,HHM,HHN,HHP,HHQ,HHR,HHS,HHT,HHV,HHW,HHY,HIA,HIC,HID,HIE,HIF,HIG,HIH,HII,HIK,HIL,HIM,HIN,HIP,HIQ,HIR,HIS,HIT,HIV,HIW,HIY,HKA,HKC,HKD,HKE,HKF,HKG,HKH,HKI,HKK,HKL,HKM,HKN,HKP,HKQ,HKR,HKS,HKT,HKV,HKW,HKY,HLA,HLC,HLD,HLE,HLF,HLG,HLH,HLI,HLK,HLL,HLM,HLN,HLP,HLQ,HLR,HLS,HLT,HLV,HLW,HLY,HMA,HMC,HMD,HME,HMF,HMG,HMH,HMI,HMK,HML,HMM,HMN,HMP,HMQ,HMR,HMS,HMT,HMV,HMW,HMY,HNA,HNC,HND,HNE,HNF,HNG,HNH,HNI,HNK,HNL,HNM,HNN,HNP,HNQ,HNR,HNS,HNT,HNV,HNW,HNY,HPA,HPC,HPD,HPE,HPF,HPG,HPH,HPI,HPK,HPL,HPM,HPN,HPP,HPQ,HPR,HPS,HPT,HPV,HPW,HPY,HQA,HQC,HQD,HQE,HQF,HQG,HQH,HQI,HQK,HQL,HQM,HQN,HQP,HQQ,HQR,HQS,HQT,HQV,HQW,HQY,HRA,HRC,HRD,HRE,HRF,HRG,HRH,HRI,HRK,HRL,HRM,HRN,HRP,HRQ,HRR,HRS,HRT,HRV,HRW,HRY,HSA,HSC,HSD,HSE,HSF,HSG,HSH,HSI,HSK,HSL,HSM,HSN,HSP,HSQ,HSR,HSS,HST,HSV,HSW,HSY,HTA,HTC,HTD,HTE,HTF,HTG,HTH,HTI,HTK,HTL,HTM,HTN,HTP,HTQ,HTR,HTS,HTT,HTV,HTW,HTY,HVA,HVC,HVD,HVE,HVF,HVG,HVH,HVI,HVK,HVL,HVM,HVN,HVP,HVQ,HVR,HVS,HVT,HVV,HVW,HVY,HWA,HWC,HWD,HWE,HWF,HWG,HWH,HWI,HWK,HWL,HWM,HWN,HWP,HWQ,HWR,HWS,HWT,HWV,HWW,HWY,HYA,HYC,HYD,HYE,HYF,HYG,HYH,HYI,HYK,HYL,HYM,HYN,HYP,HYQ,HYR,HYS,HYT,HYV,HYW,HYY,IAA,IAC,IAD,IAE,IAF,IAG,IAH,IAI,IAK,IAL,IAM,IAN,IAP,IAQ,IAR,IAS,IAT,IAV,IAW,IAY,ICA,ICC,ICD,ICE,ICF,ICG,ICH,ICI,ICK,ICL,ICM,ICN,ICP,ICQ,ICR,ICS,ICT,ICV,ICW,ICY,IDA,IDC,IDD,IDE,IDF,IDG,IDH,IDI,IDK,IDL,IDM,IDN,IDP,IDQ,IDR,IDS,IDT,IDV,IDW,IDY,IEA,IEC,IED,IEE,IEF,IEG,IEH,IEI,IEK,IEL,IEM,IEN,IEP,IEQ,IER,IES,IET,IEV,IEW,IEY,IFA,IFC,IFD,IFE,IFF,IFG,IFH,IFI,IFK,IFL,IFM,IFN,IFP,IFQ,IFR,IFS,IFT,IFV,IFW,IFY,IGA,IGC,IGD,IGE,IGF,IGG,IGH,IGI,IGK,IGL,IGM,IGN,IGP,IGQ,IGR,IGS,IGT,IGV,IGW,IGY,IHA,IHC,IHD,IHE,IHF,IHG,IHH,IHI,IHK,IHL,IHM,IHN,IHP,IHQ,IHR,IHS,IHT,IHV,IHW,IHY,IIA,IIC,IID,IIE,IIF,IIG,IIH,III,IIK,IIL,IIM,IIN,IIP,IIQ,IIR,IIS,IIT,IIV,IIW,IIY,IKA,IKC,IKD,IKE,IKF,IKG,IKH,IKI,IKK,IKL,IKM,IKN,IKP,IKQ,IKR,IKS,IKT,IKV,IKW,IKY,ILA,ILC,ILD,ILE,ILF,ILG,ILH,ILI,ILK,ILL,ILM,ILN,ILP,ILQ,ILR,ILS,ILT,ILV,ILW,ILY,IMA,IMC,IMD,IME,IMF,IMG,IMH,IMI,IMK,IML,IMM,IMN,IMP,IMQ,IMR,IMS,IMT,IMV,IMW,IMY,INA,INC,IND,INE,INF,ING,INH,INI,INK,INL,INM,INN,INP,INQ,INR,INS,INT,INV,INW,INY,IPA,IPC,IPD,IPE,IPF,IPG,IPH,IPI,IPK,IPL,IPM,IPN,IPP,IPQ,IPR,IPS,IPT,IPV,IPW,IPY,IQA,IQC,IQD,IQE,IQF,IQG,IQH,IQI,IQK,IQL,IQM,IQN,IQP,IQQ,IQR,IQS,IQT,IQV,IQW,IQY,IRA,IRC,IRD,IRE,IRF,IRG,IRH,IRI,IRK,IRL,IRM,IRN,IRP,IRQ,IRR,IRS,IRT,IRV,IRW,IRY,ISA,ISC,ISD,ISE,ISF,ISG,ISH,ISI,ISK,ISL,ISM,ISN,ISP,ISQ,ISR,ISS,IST,ISV,ISW,ISY,ITA,ITC,ITD,ITE,ITF,ITG,ITH,ITI,ITK,ITL,ITM,ITN,ITP,ITQ,ITR,ITS,ITT,ITV,ITW,ITY,IVA,IVC,IVD,IVE,IVF,IVG,IVH,IVI,IVK,IVL,IVM,IVN,IVP,IVQ,IVR,IVS,IVT,IVV,IVW,IVY,IWA,IWC,IWD,IWE,IWF,IWG,IWH,IWI,IWK,IWL,IWM,IWN,IWP,IWQ,IWR,IWS,IWT,IWV,IWW,IWY,IYA,IYC,IYD,IYE,IYF,IYG,IYH,IYI,IYK,IYL,IYM,IYN,IYP,IYQ,IYR,IYS,IYT,IYV,IYW,IYY,KAA,KAC,KAD,KAE,KAF,KAG,KAH,KAI,KAK,KAL,KAM,KAN,KAP,KAQ,KAR,KAS,KAT,KAV,KAW,KAY,KCA,KCC,KCD,KCE,KCF,KCG,KCH,KCI,KCK,KCL,KCM,KCN,KCP,KCQ,KCR,KCS,KCT,KCV,KCW,KCY,KDA,KDC,KDD,KDE,KDF,KDG,KDH,KDI,KDK,KDL,KDM,KDN,KDP,KDQ,KDR,KDS,KDT,KDV,KDW,KDY,KEA,KEC,KED,KEE,KEF,KEG,KEH,KEI,KEK,KEL,KEM,KEN,KEP,KEQ,KER,KES,KET,KEV,KEW,KEY,KFA,KFC,KFD,KFE,KFF,KFG,KFH,KFI,KFK,KFL,KFM,KFN,KFP,KFQ,KFR,KFS,KFT,KFV,KFW,KFY,KGA,KGC,KGD,KGE,KGF,KGG,KGH,KGI,KGK,KGL,KGM,KGN,KGP,KGQ,KGR,KGS,KGT,KGV,KGW,KGY,KHA,KHC,KHD,KHE,KHF,KHG,KHH,KHI,KHK,KHL,KHM,KHN,KHP,KHQ,KHR,KHS,KHT,KHV,KHW,KHY,KIA,KIC,KID,KIE,KIF,KIG,KIH,KII,KIK,KIL,KIM,KIN,KIP,KIQ,KIR,KIS,KIT,KIV,KIW,KIY,KKA,KKC,KKD,KKE,KKF,KKG,KKH,KKI,KKK,KKL,KKM,KKN,KKP,KKQ,KKR,KKS,KKT,KKV,KKW,KKY,KLA,KLC,KLD,KLE,KLF,KLG,KLH,KLI,KLK,KLL,KLM,KLN,KLP,KLQ,KLR,KLS,KLT,KLV,KLW,KLY,KMA,KMC,KMD,KME,KMF,KMG,KMH,KMI,KMK,KML,KMM,KMN,KMP,KMQ,KMR,KMS,KMT,KMV,KMW,KMY,KNA,KNC,KND,KNE,KNF,KNG,KNH,KNI,KNK,KNL,KNM,KNN,KNP,KNQ,KNR,KNS,KNT,KNV,KNW,KNY,KPA,KPC,KPD,KPE,KPF,KPG,KPH,KPI,KPK,KPL,KPM,KPN,KPP,KPQ,KPR,KPS,KPT,KPV,KPW,KPY,KQA,KQC,KQD,KQE,KQF,KQG,KQH,KQI,KQK,KQL,KQM,KQN,KQP,KQQ,KQR,KQS,KQT,KQV,KQW,KQY,KRA,KRC,KRD,KRE,KRF,KRG,KRH,KRI,KRK,KRL,KRM,KRN,KRP,KRQ,KRR,KRS,KRT,KRV,KRW,KRY,KSA,KSC,KSD,KSE,KSF,KSG,KSH,KSI,KSK,KSL,KSM,KSN,KSP,KSQ,KSR,KSS,KST,KSV,KSW,KSY,KTA,KTC,KTD,KTE,KTF,KTG,KTH,KTI,KTK,KTL,KTM,KTN,KTP,KTQ,KTR,KTS,KTT,KTV,KTW,KTY,KVA,KVC,KVD,KVE,KVF,KVG,KVH,KVI,KVK,KVL,KVM,KVN,KVP,KVQ,KVR,KVS,KVT,KVV,KVW,KVY,KWA,KWC,KWD,KWE,KWF,KWG,KWH,KWI,KWK,KWL,KWM,KWN,KWP,KWQ,KWR,KWS,KWT,KWV,KWW,KWY,KYA,KYC,KYD,KYE,KYF,KYG,KYH,KYI,KYK,KYL,KYM,KYN,KYP,KYQ,KYR,KYS,KYT,KYV,KYW,KYY,LAA,LAC,LAD,LAE,LAF,LAG,LAH,LAI,LAK,LAL,LAM,LAN,LAP,LAQ,LAR,LAS,LAT,LAV,LAW,LAY,LCA,LCC,LCD,LCE,LCF,LCG,LCH,LCI,LCK,LCL,LCM,LCN,LCP,LCQ,LCR,LCS,LCT,LCV,LCW,LCY,LDA,LDC,LDD,LDE,LDF,LDG,LDH,LDI,LDK,LDL,LDM,LDN,LDP,LDQ,LDR,LDS,LDT,LDV,LDW,LDY,LEA,LEC,LED,LEE,LEF,LEG,LEH,LEI,LEK,LEL,LEM,LEN,LEP,LEQ,LER,LES,LET,LEV,LEW,LEY,LFA,LFC,LFD,LFE,LFF,LFG,LFH,LFI,LFK,LFL,LFM,LFN,LFP,LFQ,LFR,LFS,LFT,LFV,LFW,LFY,LGA,LGC,LGD,LGE,LGF,LGG,LGH,LGI,LGK,LGL,LGM,LGN,LGP,LGQ,LGR,LGS,LGT,LGV,LGW,LGY,LHA,LHC,LHD,LHE,LHF,LHG,LHH,LHI,LHK,LHL,LHM,LHN,LHP,LHQ,LHR,LHS,LHT,LHV,LHW,LHY,LIA,LIC,LID,LIE,LIF,LIG,LIH,LII,LIK,LIL,LIM,LIN,LIP,LIQ,LIR,LIS,LIT,LIV,LIW,LIY,LKA,LKC,LKD,LKE,LKF,LKG,LKH,LKI,LKK,LKL,LKM,LKN,LKP,LKQ,LKR,LKS,LKT,LKV,LKW,LKY,LLA,LLC,LLD,LLE,LLF,LLG,LLH,LLI,LLK,LLL,LLM,LLN,LLP,LLQ,LLR,LLS,LLT,LLV,LLW,LLY,LMA,LMC,LMD,LME,LMF,LMG,LMH,LMI,LMK,LML,LMM,LMN,LMP,LMQ,LMR,LMS,LMT,LMV,LMW,LMY,LNA,LNC,LND,LNE,LNF,LNG,LNH,LNI,LNK,LNL,LNM,LNN,LNP,LNQ,LNR,LNS,LNT,LNV,LNW,LNY,LPA,LPC,LPD,LPE,LPF,LPG,LPH,LPI,LPK,LPL,LPM,LPN,LPP,LPQ,LPR,LPS,LPT,LPV,LPW,LPY,LQA,LQC,LQD,LQE,LQF,LQG,LQH,LQI,LQK,LQL,LQM,LQN,LQP,LQQ,LQR,LQS,LQT,LQV,LQW,LQY,LRA,LRC,LRD,LRE,LRF,LRG,LRH,LRI,LRK,LRL,LRM,LRN,LRP,LRQ,LRR,LRS,LRT,LRV,LRW,LRY,LSA,LSC,LSD,LSE,LSF,LSG,LSH,LSI,LSK,LSL,LSM,LSN,LSP,LSQ,LSR,LSS,LST,LSV,LSW,LSY,LTA,LTC,LTD,LTE,LTF,LTG,LTH,LTI,LTK,LTL,LTM,LTN,LTP,LTQ,LTR,LTS,LTT,LTV,LTW,LTY,LVA,LVC,LVD,LVE,LVF,LVG,LVH,LVI,LVK,LVL,LVM,LVN,LVP,LVQ,LVR,LVS,LVT,LVV,LVW,LVY,LWA,LWC,LWD,LWE,LWF,LWG,LWH,LWI,LWK,LWL,LWM,LWN,LWP,LWQ,LWR,LWS,LWT,LWV,LWW,LWY,LYA,LYC,LYD,LYE,LYF,LYG,LYH,LYI,LYK,LYL,LYM,LYN,LYP,LYQ,LYR,LYS,LYT,LYV,LYW,LYY,MAA,MAC,MAD,MAE,MAF,MAG,MAH,MAI,MAK,MAL,MAM,MAN,MAP,MAQ,MAR,MAS,MAT,MAV,MAW,MAY,MCA,MCC,MCD,MCE,MCF,MCG,MCH,MCI,MCK,MCL,MCM,MCN,MCP,MCQ,MCR,MCS,MCT,MCV,MCW,MCY,MDA,MDC,MDD,MDE,MDF,MDG,MDH,MDI,MDK,MDL,MDM,MDN,MDP,MDQ,MDR,MDS,MDT,MDV,MDW,MDY,MEA,MEC,MED,MEE,MEF,MEG,MEH,MEI,MEK,MEL,MEM,MEN,MEP,MEQ,MER,MES,MET,MEV,MEW,MEY,MFA,MFC,MFD,MFE,MFF,MFG,MFH,MFI,MFK,MFL,MFM,MFN,MFP,MFQ,MFR,MFS,MFT,MFV,MFW,MFY,MGA,MGC,MGD,MGE,MGF,MGG,MGH,MGI,MGK,MGL,MGM,MGN,MGP,MGQ,MGR,MGS,MGT,MGV,MGW,MGY,MHA,MHC,MHD,MHE,MHF,MHG,MHH,MHI,MHK,MHL,MHM,MHN,MHP,MHQ,MHR,MHS,MHT,MHV,MHW,MHY,MIA,MIC,MID,MIE,MIF,MIG,MIH,MII,MIK,MIL,MIM,MIN,MIP,MIQ,MIR,MIS,MIT,MIV,MIW,MIY,MKA,MKC,MKD,MKE,MKF,MKG,MKH,MKI,MKK,MKL,MKM,MKN,MKP,MKQ,MKR,MKS,MKT,MKV,MKW,MKY,MLA,MLC,MLD,MLE,MLF,MLG,MLH,MLI,MLK,MLL,MLM,MLN,MLP,MLQ,MLR,MLS,MLT,MLV,MLW,MLY,MMA,MMC,MMD,MME,MMF,MMG,MMH,MMI,MMK,MML,MMM,MMN,MMP,MMQ,MMR,MMS,MMT,MMV,MMW,MMY,MNA,MNC,MND,MNE,MNF,MNG,MNH,MNI,MNK,MNL,MNM,MNN,MNP,MNQ,MNR,MNS,MNT,MNV,MNW,MNY,MPA,MPC,MPD,MPE,MPF,MPG,MPH,MPI,MPK,MPL,MPM,MPN,MPP,MPQ,MPR,MPS,MPT,MPV,MPW,MPY,MQA,MQC,MQD,MQE,MQF,MQG,MQH,MQI,MQK,MQL,MQM,MQN,MQP,MQQ,MQR,MQS,MQT,MQV,MQW,MQY,MRA,MRC,MRD,MRE,MRF,MRG,MRH,MRI,MRK,MRL,MRM,MRN,MRP,MRQ,MRR,MRS,MRT,MRV,MRW,MRY,MSA,MSC,MSD,MSE,MSF,MSG,MSH,MSI,MSK,MSL,MSM,MSN,MSP,MSQ,MSR,MSS,MST,MSV,MSW,MSY,MTA,MTC,MTD,MTE,MTF,MTG,MTH,MTI,MTK,MTL,MTM,MTN,MTP,MTQ,MTR,MTS,MTT,MTV,MTW,MTY,MVA,MVC,MVD,MVE,MVF,MVG,MVH,MVI,MVK,MVL,MVM,MVN,MVP,MVQ,MVR,MVS,MVT,MVV,MVW,MVY,MWA,MWC,MWD,MWE,MWF,MWG,MWH,MWI,MWK,MWL,MWM,MWN,MWP,MWQ,MWR,MWS,MWT,MWV,MWW,MWY,MYA,MYC,MYD,MYE,MYF,MYG,MYH,MYI,MYK,MYL,MYM,MYN,MYP,MYQ,MYR,MYS,MYT,MYV,MYW,MYY,NAA,NAC,NAD,NAE,NAF,NAG,NAH,NAI,NAK,NAL,NAM,NAN,NAP,NAQ,NAR,NAS,NAT,NAV,NAW,NAY,NCA,NCC,NCD,NCE,NCF,NCG,NCH,NCI,NCK,NCL,NCM,NCN,NCP,NCQ,NCR,NCS,NCT,NCV,NCW,NCY,NDA,NDC,NDD,NDE,NDF,NDG,NDH,NDI,NDK,NDL,NDM,NDN,NDP,NDQ,NDR,NDS,NDT,NDV,NDW,NDY,NEA,NEC,NED,NEE,NEF,NEG,NEH,NEI,NEK,NEL,NEM,NEN,NEP,NEQ,NER,NES,NET,NEV,NEW,NEY,NFA,NFC,NFD,NFE,NFF,NFG,NFH,NFI,NFK,NFL,NFM,NFN,NFP,NFQ,NFR,NFS,NFT,NFV,NFW,NFY,NGA,NGC,NGD,NGE,NGF,NGG,NGH,NGI,NGK,NGL,NGM,NGN,NGP,NGQ,NGR,NGS,NGT,NGV,NGW,NGY,NHA,NHC,NHD,NHE,NHF,NHG,NHH,NHI,NHK,NHL,NHM,NHN,NHP,NHQ,NHR,NHS,NHT,NHV,NHW,NHY,NIA,NIC,NID,NIE,NIF,NIG,NIH,NII,NIK,NIL,NIM,NIN,NIP,NIQ,NIR,NIS,NIT,NIV,NIW,NIY,NKA,NKC,NKD,NKE,NKF,NKG,NKH,NKI,NKK,NKL,NKM,NKN,NKP,NKQ,NKR,NKS,NKT,NKV,NKW,NKY,NLA,NLC,NLD,NLE,NLF,NLG,NLH,NLI,NLK,NLL,NLM,NLN,NLP,NLQ,NLR,NLS,NLT,NLV,NLW,NLY,NMA,NMC,NMD,NME,NMF,NMG,NMH,NMI,NMK,NML,NMM,NMN,NMP,NMQ,NMR,NMS,NMT,NMV,NMW,NMY,NNA,NNC,NND,NNE,NNF,NNG,NNH,NNI,NNK,NNL,NNM,NNN,NNP,NNQ,NNR,NNS,NNT,NNV,NNW,NNY,NPA,NPC,NPD,NPE,NPF,NPG,NPH,NPI,NPK,NPL,NPM,NPN,NPP,NPQ,NPR,NPS,NPT,NPV,NPW,NPY,NQA,NQC,NQD,NQE,NQF,NQG,NQH,NQI,NQK,NQL,NQM,NQN,NQP,NQQ,NQR,NQS,NQT,NQV,NQW,NQY,NRA,NRC,NRD,NRE,NRF,NRG,NRH,NRI,NRK,NRL,NRM,NRN,NRP,NRQ,NRR,NRS,NRT,NRV,NRW,NRY,NSA,NSC,NSD,NSE,NSF,NSG,NSH,NSI,NSK,NSL,NSM,NSN,NSP,NSQ,NSR,NSS,NST,NSV,NSW,NSY,NTA,NTC,NTD,NTE,NTF,NTG,NTH,NTI,NTK,NTL,NTM,NTN,NTP,NTQ,NTR,NTS,NTT,NTV,NTW,NTY,NVA,NVC,NVD,NVE,NVF,NVG,NVH,NVI,NVK,NVL,NVM,NVN,NVP,NVQ,NVR,NVS,NVT,NVV,NVW,NVY,NWA,NWC,NWD,NWE,NWF,NWG,NWH,NWI,NWK,NWL,NWM,NWN,NWP,NWQ,NWR,NWS,NWT,NWV,NWW,NWY,NYA,NYC,NYD,NYE,NYF,NYG,NYH,NYI,NYK,NYL,NYM,NYN,NYP,NYQ,NYR,NYS,NYT,NYV,NYW,NYY,PAA,PAC,PAD,PAE,PAF,PAG,PAH,PAI,PAK,PAL,PAM,PAN,PAP,PAQ,PAR,PAS,PAT,PAV,PAW,PAY,PCA,PCC,PCD,PCE,PCF,PCG,PCH,PCI,PCK,PCL,PCM,PCN,PCP,PCQ,PCR,PCS,PCT,PCV,PCW,PCY,PDA,PDC,PDD,PDE,PDF,PDG,PDH,PDI,PDK,PDL,PDM,PDN,PDP,PDQ,PDR,PDS,PDT,PDV,PDW,PDY,PEA,PEC,PED,PEE,PEF,PEG,PEH,PEI,PEK,PEL,PEM,PEN,PEP,PEQ,PER,PES,PET,PEV,PEW,PEY,PFA,PFC,PFD,PFE,PFF,PFG,PFH,PFI,PFK,PFL,PFM,PFN,PFP,PFQ,PFR,PFS,PFT,PFV,PFW,PFY,PGA,PGC,PGD,PGE,PGF,PGG,PGH,PGI,PGK,PGL,PGM,PGN,PGP,PGQ,PGR,PGS,PGT,PGV,PGW,PGY,PHA,PHC,PHD,PHE,PHF,PHG,PHH,PHI,PHK,PHL,PHM,PHN,PHP,PHQ,PHR,PHS,PHT,PHV,PHW,PHY,PIA,PIC,PID,PIE,PIF,PIG,PIH,PII,PIK,PIL,PIM,PIN,PIP,PIQ,PIR,PIS,PIT,PIV,PIW,PIY,PKA,PKC,PKD,PKE,PKF,PKG,PKH,PKI,PKK,PKL,PKM,PKN,PKP,PKQ,PKR,PKS,PKT,PKV,PKW,PKY,PLA,PLC,PLD,PLE,PLF,PLG,PLH,PLI,PLK,PLL,PLM,PLN,PLP,PLQ,PLR,PLS,PLT,PLV,PLW,PLY,PMA,PMC,PMD,PME,PMF,PMG,PMH,PMI,PMK,PML,PMM,PMN,PMP,PMQ,PMR,PMS,PMT,PMV,PMW,PMY,PNA,PNC,PND,PNE,PNF,PNG,PNH,PNI,PNK,PNL,PNM,PNN,PNP,PNQ,PNR,PNS,PNT,PNV,PNW,PNY,PPA,PPC,PPD,PPE,PPF,PPG,PPH,PPI,PPK,PPL,PPM,PPN,PPP,PPQ,PPR,PPS,PPT,PPV,PPW,PPY,PQA,PQC,PQD,PQE,PQF,PQG,PQH,PQI,PQK,PQL,PQM,PQN,PQP,PQQ,PQR,PQS,PQT,PQV,PQW,PQY,PRA,PRC,PRD,PRE,PRF,PRG,PRH,PRI,PRK,PRL,PRM,PRN,PRP,PRQ,PRR,PRS,PRT,PRV,PRW,PRY,PSA,PSC,PSD,PSE,PSF,PSG,PSH,PSI,PSK,PSL,PSM,PSN,PSP,PSQ,PSR,PSS,PST,PSV,PSW,PSY,PTA,PTC,PTD,PTE,PTF,PTG,PTH,PTI,PTK,PTL,PTM,PTN,PTP,PTQ,PTR,PTS,PTT,PTV,PTW,PTY,PVA,PVC,PVD,PVE,PVF,PVG,PVH,PVI,PVK,PVL,PVM,PVN,PVP,PVQ,PVR,PVS,PVT,PVV,PVW,PVY,PWA,PWC,PWD,PWE,PWF,PWG,PWH,PWI,PWK,PWL,PWM,PWN,PWP,PWQ,PWR,PWS,PWT,PWV,PWW,PWY,PYA,PYC,PYD,PYE,PYF,PYG,PYH,PYI,PYK,PYL,PYM,PYN,PYP,PYQ,PYR,PYS,PYT,PYV,PYW,PYY,QAA,QAC,QAD,QAE,QAF,QAG,QAH,QAI,QAK,QAL,QAM,QAN,QAP,QAQ,QAR,QAS,QAT,QAV,QAW,QAY,QCA,QCC,QCD,QCE,QCF,QCG,QCH,QCI,QCK,QCL,QCM,QCN,QCP,QCQ,QCR,QCS,QCT,QCV,QCW,QCY,QDA,QDC,QDD,QDE,QDF,QDG,QDH,QDI,QDK,QDL,QDM,QDN,QDP,QDQ,QDR,QDS,QDT,QDV,QDW,QDY,QEA,QEC,QED,QEE,QEF,QEG,QEH,QEI,QEK,QEL,QEM,QEN,QEP,QEQ,QER,QES,QET,QEV,QEW,QEY,QFA,QFC,QFD,QFE,QFF,QFG,QFH,QFI,QFK,QFL,QFM,QFN,QFP,QFQ,QFR,QFS,QFT,QFV,QFW,QFY,QGA,QGC,QGD,QGE,QGF,QGG,QGH,QGI,QGK,QGL,QGM,QGN,QGP,QGQ,QGR,QGS,QGT,QGV,QGW,QGY,QHA,QHC,QHD,QHE,QHF,QHG,QHH,QHI,QHK,QHL,QHM,QHN,QHP,QHQ,QHR,QHS,QHT,QHV,QHW,QHY,QIA,QIC,QID,QIE,QIF,QIG,QIH,QII,QIK,QIL,QIM,QIN,QIP,QIQ,QIR,QIS,QIT,QIV,QIW,QIY,QKA,QKC,QKD,QKE,QKF,QKG,QKH,QKI,QKK,QKL,QKM,QKN,QKP,QKQ,QKR,QKS,QKT,QKV,QKW,QKY,QLA,QLC,QLD,QLE,QLF,QLG,QLH,QLI,QLK,QLL,QLM,QLN,QLP,QLQ,QLR,QLS,QLT,QLV,QLW,QLY,QMA,QMC,QMD,QME,QMF,QMG,QMH,QMI,QMK,QML,QMM,QMN,QMP,QMQ,QMR,QMS,QMT,QMV,QMW,QMY,QNA,QNC,QND,QNE,QNF,QNG,QNH,QNI,QNK,QNL,QNM,QNN,QNP,QNQ,QNR,QNS,QNT,QNV,QNW,QNY,QPA,QPC,QPD,QPE,QPF,QPG,QPH,QPI,QPK,QPL,QPM,QPN,QPP,QPQ,QPR,QPS,QPT,QPV,QPW,QPY,QQA,QQC,QQD,QQE,QQF,QQG,QQH,QQI,QQK,QQL,QQM,QQN,QQP,QQQ,QQR,QQS,QQT,QQV,QQW,QQY,QRA,QRC,QRD,QRE,QRF,QRG,QRH,QRI,QRK,QRL,QRM,QRN,QRP,QRQ,QRR,QRS,QRT,QRV,QRW,QRY,QSA,QSC,QSD,QSE,QSF,QSG,QSH,QSI,QSK,QSL,QSM,QSN,QSP,QSQ,QSR,QSS,QST,QSV,QSW,QSY,QTA,QTC,QTD,QTE,QTF,QTG,QTH,QTI,QTK,QTL,QTM,QTN,QTP,QTQ,QTR,QTS,QTT,QTV,QTW,QTY,QVA,QVC,QVD,QVE,QVF,QVG,QVH,QVI,QVK,QVL,QVM,QVN,QVP,QVQ,QVR,QVS,QVT,QVV,QVW,QVY,QWA,QWC,QWD,QWE,QWF,QWG,QWH,QWI,QWK,QWL,QWM,QWN,QWP,QWQ,QWR,QWS,QWT,QWV,QWW,QWY,QYA,QYC,QYD,QYE,QYF,QYG,QYH,QYI,QYK,QYL,QYM,QYN,QYP,QYQ,QYR,QYS,QYT,QYV,QYW,QYY,RAA,RAC,RAD,RAE,RAF,RAG,RAH,RAI,RAK,RAL,RAM,RAN,RAP,RAQ,RAR,RAS,RAT,RAV,RAW,RAY,RCA,RCC,RCD,RCE,RCF,RCG,RCH,RCI,RCK,RCL,RCM,RCN,RCP,RCQ,RCR,RCS,RCT,RCV,RCW,RCY,RDA,RDC,RDD,RDE,RDF,RDG,RDH,RDI,RDK,RDL,RDM,RDN,RDP,RDQ,RDR,RDS,RDT,RDV,RDW,RDY,REA,REC,RED,REE,REF,REG,REH,REI,REK,REL,REM,REN,REP,REQ,RER,RES,RET,REV,REW,REY,RFA,RFC,RFD,RFE,RFF,RFG,RFH,RFI,RFK,RFL,RFM,RFN,RFP,RFQ,RFR,RFS,RFT,RFV,RFW,RFY,RGA,RGC,RGD,RGE,RGF,RGG,RGH,RGI,RGK,RGL,RGM,RGN,RGP,RGQ,RGR,RGS,RGT,RGV,RGW,RGY,RHA,RHC,RHD,RHE,RHF,RHG,RHH,RHI,RHK,RHL,RHM,RHN,RHP,RHQ,RHR,RHS,RHT,RHV,RHW,RHY,RIA,RIC,RID,RIE,RIF,RIG,RIH,RII,RIK,RIL,RIM,RIN,RIP,RIQ,RIR,RIS,RIT,RIV,RIW,RIY,RKA,RKC,RKD,RKE,RKF,RKG,RKH,RKI,RKK,RKL,RKM,RKN,RKP,RKQ,RKR,RKS,RKT,RKV,RKW,RKY,RLA,RLC,RLD,RLE,RLF,RLG,RLH,RLI,RLK,RLL,RLM,RLN,RLP,RLQ,RLR,RLS,RLT,RLV,RLW,RLY,RMA,RMC,RMD,RME,RMF,RMG,RMH,RMI,RMK,RML,RMM,RMN,RMP,RMQ,RMR,RMS,RMT,RMV,RMW,RMY,RNA,RNC,RND,RNE,RNF,RNG,RNH,RNI,RNK,RNL,RNM,RNN,RNP,RNQ,RNR,RNS,RNT,RNV,RNW,RNY,RPA,RPC,RPD,RPE,RPF,RPG,RPH,RPI,RPK,RPL,RPM,RPN,RPP,RPQ,RPR,RPS,RPT,RPV,RPW,RPY,RQA,RQC,RQD,RQE,RQF,RQG,RQH,RQI,RQK,RQL,RQM,RQN,RQP,RQQ,RQR,RQS,RQT,RQV,RQW,RQY,RRA,RRC,RRD,RRE,RRF,RRG,RRH,RRI,RRK,RRL,RRM,RRN,RRP,RRQ,RRR,RRS,RRT,RRV,RRW,RRY,RSA,RSC,RSD,RSE,RSF,RSG,RSH,RSI,RSK,RSL,RSM,RSN,RSP,RSQ,RSR,RSS,RST,RSV,RSW,RSY,RTA,RTC,RTD,RTE,RTF,RTG,RTH,RTI,RTK,RTL,RTM,RTN,RTP,RTQ,RTR,RTS,RTT,RTV,RTW,RTY,RVA,RVC,RVD,RVE,RVF,RVG,RVH,RVI,RVK,RVL,RVM,RVN,RVP,RVQ,RVR,RVS,RVT,RVV,RVW,RVY,RWA,RWC,RWD,RWE,RWF,RWG,RWH,RWI,RWK,RWL,RWM,RWN,RWP,RWQ,RWR,RWS,RWT,RWV,RWW,RWY,RYA,RYC,RYD,RYE,RYF,RYG,RYH,RYI,RYK,RYL,RYM,RYN,RYP,RYQ,RYR,RYS,RYT,RYV,RYW,RYY,SAA,SAC,SAD,SAE,SAF,SAG,SAH,SAI,SAK,SAL,SAM,SAN,SAP,SAQ,SAR,SAS,SAT,SAV,SAW,SAY,SCA,SCC,SCD,SCE,SCF,SCG,SCH,SCI,SCK,SCL,SCM,SCN,SCP,SCQ,SCR,SCS,SCT,SCV,SCW,SCY,SDA,SDC,SDD,SDE,SDF,SDG,SDH,SDI,SDK,SDL,SDM,SDN,SDP,SDQ,SDR,SDS,SDT,SDV,SDW,SDY,SEA,SEC,SED,SEE,SEF,SEG,SEH,SEI,SEK,SEL,SEM,SEN,SEP,SEQ,SER,SES,SET,SEV,SEW,SEY,SFA,SFC,SFD,SFE,SFF,SFG,SFH,SFI,SFK,SFL,SFM,SFN,SFP,SFQ,SFR,SFS,SFT,SFV,SFW,SFY,SGA,SGC,SGD,SGE,SGF,SGG,SGH,SGI,SGK,SGL,SGM,SGN,SGP,SGQ,SGR,SGS,SGT,SGV,SGW,SGY,SHA,SHC,SHD,SHE,SHF,SHG,SHH,SHI,SHK,SHL,SHM,SHN,SHP,SHQ,SHR,SHS,SHT,SHV,SHW,SHY,SIA,SIC,SID,SIE,SIF,SIG,SIH,SII,SIK,SIL,SIM,SIN,SIP,SIQ,SIR,SIS,SIT,SIV,SIW,SIY,SKA,SKC,SKD,SKE,SKF,SKG,SKH,SKI,SKK,SKL,SKM,SKN,SKP,SKQ,SKR,SKS,SKT,SKV,SKW,SKY,SLA,SLC,SLD,SLE,SLF,SLG,SLH,SLI,SLK,SLL,SLM,SLN,SLP,SLQ,SLR,SLS,SLT,SLV,SLW,SLY,SMA,SMC,SMD,SME,SMF,SMG,SMH,SMI,SMK,SML,SMM,SMN,SMP,SMQ,SMR,SMS,SMT,SMV,SMW,SMY,SNA,SNC,SND,SNE,SNF,SNG,SNH,SNI,SNK,SNL,SNM,SNN,SNP,SNQ,SNR,SNS,SNT,SNV,SNW,SNY,SPA,SPC,SPD,SPE,SPF,SPG,SPH,SPI,SPK,SPL,SPM,SPN,SPP,SPQ,SPR,SPS,SPT,SPV,SPW,SPY,SQA,SQC,SQD,SQE,SQF,SQG,SQH,SQI,SQK,SQL,SQM,SQN,SQP,SQQ,SQR,SQS,SQT,SQV,SQW,SQY,SRA,SRC,SRD,SRE,SRF,SRG,SRH,SRI,SRK,SRL,SRM,SRN,SRP,SRQ,SRR,SRS,SRT,SRV,SRW,SRY,SSA,SSC,SSD,SSE,SSF,SSG,SSH,SSI,SSK,SSL,SSM,SSN,SSP,SSQ,SSR,SSS,SST,SSV,SSW,SSY,STA,STC,STD,STE,STF,STG,STH,STI,STK,STL,STM,STN,STP,STQ,STR,STS,STT,STV,STW,STY,SVA,SVC,SVD,SVE,SVF,SVG,SVH,SVI,SVK,SVL,SVM,SVN,SVP,SVQ,SVR,SVS,SVT,SVV,SVW,SVY,SWA,SWC,SWD,SWE,SWF,SWG,SWH,SWI,SWK,SWL,SWM,SWN,SWP,SWQ,SWR,SWS,SWT,SWV,SWW,SWY,SYA,SYC,SYD,SYE,SYF,SYG,SYH,SYI,SYK,SYL,SYM,SYN,SYP,SYQ,SYR,SYS,SYT,SYV,SYW,SYY,TAA,TAC,TAD,TAE,TAF,TAG,TAH,TAI,TAK,TAL,TAM,TAN,TAP,TAQ,TAR,TAS,TAT,TAV,TAW,TAY,TCA,TCC,TCD,TCE,TCF,TCG,TCH,TCI,TCK,TCL,TCM,TCN,TCP,TCQ,TCR,TCS,TCT,TCV,TCW,TCY,TDA,TDC,TDD,TDE,TDF,TDG,TDH,TDI,TDK,TDL,TDM,TDN,TDP,TDQ,TDR,TDS,TDT,TDV,TDW,TDY,TEA,TEC,TED,TEE,TEF,TEG,TEH,TEI,TEK,TEL,TEM,TEN,TEP,TEQ,TER,TES,TET,TEV,TEW,TEY,TFA,TFC,TFD,TFE,TFF,TFG,TFH,TFI,TFK,TFL,TFM,TFN,TFP,TFQ,TFR,TFS,TFT,TFV,TFW,TFY,TGA,TGC,TGD,TGE,TGF,TGG,TGH,TGI,TGK,TGL,TGM,TGN,TGP,TGQ,TGR,TGS,TGT,TGV,TGW,TGY,THA,THC,THD,THE,THF,THG,THH,THI,THK,THL,THM,THN,THP,THQ,THR,THS,THT,THV,THW,THY,TIA,TIC,TID,TIE,TIF,TIG,TIH,TII,TIK,TIL,TIM,TIN,TIP,TIQ,TIR,TIS,TIT,TIV,TIW,TIY,TKA,TKC,TKD,TKE,TKF,TKG,TKH,TKI,TKK,TKL,TKM,TKN,TKP,TKQ,TKR,TKS,TKT,TKV,TKW,TKY,TLA,TLC,TLD,TLE,TLF,TLG,TLH,TLI,TLK,TLL,TLM,TLN,TLP,TLQ,TLR,TLS,TLT,TLV,TLW,TLY,TMA,TMC,TMD,TME,TMF,TMG,TMH,TMI,TMK,TML,TMM,TMN,TMP,TMQ,TMR,TMS,TMT,TMV,TMW,TMY,TNA,TNC,TND,TNE,TNF,TNG,TNH,TNI,TNK,TNL,TNM,TNN,TNP,TNQ,TNR,TNS,TNT,TNV,TNW,TNY,TPA,TPC,TPD,TPE,TPF,TPG,TPH,TPI,TPK,TPL,TPM,TPN,TPP,TPQ,TPR,TPS,TPT,TPV,TPW,TPY,TQA,TQC,TQD,TQE,TQF,TQG,TQH,TQI,TQK,TQL,TQM,TQN,TQP,TQQ,TQR,TQS,TQT,TQV,TQW,TQY,TRA,TRC,TRD,TRE,TRF,TRG,TRH,TRI,TRK,TRL,TRM,TRN,TRP,TRQ,TRR,TRS,TRT,TRV,TRW,TRY,TSA,TSC,TSD,TSE,TSF,TSG,TSH,TSI,TSK,TSL,TSM,TSN,TSP,TSQ,TSR,TSS,TST,TSV,TSW,TSY,TTA,TTC,TTD,TTE,TTF,TTG,TTH,TTI,TTK,TTL,TTM,TTN,TTP,TTQ,TTR,TTS,TTT,TTV,TTW,TTY,TVA,TVC,TVD,TVE,TVF,TVG,TVH,TVI,TVK,TVL,TVM,TVN,TVP,TVQ,TVR,TVS,TVT,TVV,TVW,TVY,TWA,TWC,TWD,TWE,TWF,TWG,TWH,TWI,TWK,TWL,TWM,TWN,TWP,TWQ,TWR,TWS,TWT,TWV,TWW,TWY,TYA,TYC,TYD,TYE,TYF,TYG,TYH,TYI,TYK,TYL,TYM,TYN,TYP,TYQ,TYR,TYS,TYT,TYV,TYW,TYY,VAA,VAC,VAD,VAE,VAF,VAG,VAH,VAI,VAK,VAL,VAM,VAN,VAP,VAQ,VAR,VAS,VAT,VAV,VAW,VAY,VCA,VCC,VCD,VCE,VCF,VCG,VCH,VCI,VCK,VCL,VCM,VCN,VCP,VCQ,VCR,VCS,VCT,VCV,VCW,VCY,VDA,VDC,VDD,VDE,VDF,VDG,VDH,VDI,VDK,VDL,VDM,VDN,VDP,VDQ,VDR,VDS,VDT,VDV,VDW,VDY,VEA,VEC,VED,VEE,VEF,VEG,VEH,VEI,VEK,VEL,VEM,VEN,VEP,VEQ,VER,VES,VET,VEV,VEW,VEY,VFA,VFC,VFD,VFE,VFF,VFG,VFH,VFI,VFK,VFL,VFM,VFN,VFP,VFQ,VFR,VFS,VFT,VFV,VFW,VFY,VGA,VGC,VGD,VGE,VGF,VGG,VGH,VGI,VGK,VGL,VGM,VGN,VGP,VGQ,VGR,VGS,VGT,VGV,VGW,VGY,VHA,VHC,VHD,VHE,VHF,VHG,VHH,VHI,VHK,VHL,VHM,VHN,VHP,VHQ,VHR,VHS,VHT,VHV,VHW,VHY,VIA,VIC,VID,VIE,VIF,VIG,VIH,VII,VIK,VIL,VIM,VIN,VIP,VIQ,VIR,VIS,VIT,VIV,VIW,VIY,VKA,VKC,VKD,VKE,VKF,VKG,VKH,VKI,VKK,VKL,VKM,VKN,VKP,VKQ,VKR,VKS,VKT,VKV,VKW,VKY,VLA,VLC,VLD,VLE,VLF,VLG,VLH,VLI,VLK,VLL,VLM,VLN,VLP,VLQ,VLR,VLS,VLT,VLV,VLW,VLY,VMA,VMC,VMD,VME,VMF,VMG,VMH,VMI,VMK,VML,VMM,VMN,VMP,VMQ,VMR,VMS,VMT,VMV,VMW,VMY,VNA,VNC,VND,VNE,VNF,VNG,VNH,VNI,VNK,VNL,VNM,VNN,VNP,VNQ,VNR,VNS,VNT,VNV,VNW,VNY,VPA,VPC,VPD,VPE,VPF,VPG,VPH,VPI,VPK,VPL,VPM,VPN,VPP,VPQ,VPR,VPS,VPT,VPV,VPW,VPY,VQA,VQC,VQD,VQE,VQF,VQG,VQH,VQI,VQK,VQL,VQM,VQN,VQP,VQQ,VQR,VQS,VQT,VQV,VQW,VQY,VRA,VRC,VRD,VRE,VRF,VRG,VRH,VRI,VRK,VRL,VRM,VRN,VRP,VRQ,VRR,VRS,VRT,VRV,VRW,VRY,VSA,VSC,VSD,VSE,VSF,VSG,VSH,VSI,VSK,VSL,VSM,VSN,VSP,VSQ,VSR,VSS,VST,VSV,VSW,VSY,VTA,VTC,VTD,VTE,VTF,VTG,VTH,VTI,VTK,VTL,VTM,VTN,VTP,VTQ,VTR,VTS,VTT,VTV,VTW,VTY,VVA,VVC,VVD,VVE,VVF,VVG,VVH,VVI,VVK,VVL,VVM,VVN,VVP,VVQ,VVR,VVS,VVT,VVV,VVW,VVY,VWA,VWC,VWD,VWE,VWF,VWG,VWH,VWI,VWK,VWL,VWM,VWN,VWP,VWQ,VWR,VWS,VWT,VWV,VWW,VWY,VYA,VYC,VYD,VYE,VYF,VYG,VYH,VYI,VYK,VYL,VYM,VYN,VYP,VYQ,VYR,VYS,VYT,VYV,VYW,VYY,WAA,WAC,WAD,WAE,WAF,WAG,WAH,WAI,WAK,WAL,WAM,WAN,WAP,WAQ,WAR,WAS,WAT,WAV,WAW,WAY,WCA,WCC,WCD,WCE,WCF,WCG,WCH,WCI,WCK,WCL,WCM,WCN,WCP,WCQ,WCR,WCS,WCT,WCV,WCW,WCY,WDA,WDC,WDD,WDE,WDF,WDG,WDH,WDI,WDK,WDL,WDM,WDN,WDP,WDQ,WDR,WDS,WDT,WDV,WDW,WDY,WEA,WEC,WED,WEE,WEF,WEG,WEH,WEI,WEK,WEL,WEM,WEN,WEP,WEQ,WER,WES,WET,WEV,WEW,WEY,WFA,WFC,WFD,WFE,WFF,WFG,WFH,WFI,WFK,WFL,WFM,WFN,WFP,WFQ,WFR,WFS,WFT,WFV,WFW,WFY,WGA,WGC,WGD,WGE,WGF,WGG,WGH,WGI,WGK,WGL,WGM,WGN,WGP,WGQ,WGR,WGS,WGT,WGV,WGW,WGY,WHA,WHC,WHD,WHE,WHF,WHG,WHH,WHI,WHK,WHL,WHM,WHN,WHP,WHQ,WHR,WHS,WHT,WHV,WHW,WHY,WIA,WIC,WID,WIE,WIF,WIG,WIH,WII,WIK,WIL,WIM,WIN,WIP,WIQ,WIR,WIS,WIT,WIV,WIW,WIY,WKA,WKC,WKD,WKE,WKF,WKG,WKH,WKI,WKK,WKL,WKM,WKN,WKP,WKQ,WKR,WKS,WKT,WKV,WKW,WKY,WLA,WLC,WLD,WLE,WLF,WLG,WLH,WLI,WLK,WLL,WLM,WLN,WLP,WLQ,WLR,WLS,WLT,WLV,WLW,WLY,WMA,WMC,WMD,WME,WMF,WMG,WMH,WMI,WMK,WML,WMM,WMN,WMP,WMQ,WMR,WMS,WMT,WMV,WMW,WMY,WNA,WNC,WND,WNE,WNF,WNG,WNH,WNI,WNK,WNL,WNM,WNN,WNP,WNQ,WNR,WNS,WNT,WNV,WNW,WNY,WPA,WPC,WPD,WPE,WPF,WPG,WPH,WPI,WPK,WPL,WPM,WPN,WPP,WPQ,WPR,WPS,WPT,WPV,WPW,WPY,WQA,WQC,WQD,WQE,WQF,WQG,WQH,WQI,WQK,WQL,WQM,WQN,WQP,WQQ,WQR,WQS,WQT,WQV,WQW,WQY,WRA,WRC,WRD,WRE,WRF,WRG,WRH,WRI,WRK,WRL,WRM,WRN,WRP,WRQ,WRR,WRS,WRT,WRV,WRW,WRY,WSA,WSC,WSD,WSE,WSF,WSG,WSH,WSI,WSK,WSL,WSM,WSN,WSP,WSQ,WSR,WSS,WST,WSV,WSW,WSY,WTA,WTC,WTD,WTE,WTF,WTG,WTH,WTI,WTK,WTL,WTM,WTN,WTP,WTQ,WTR,WTS,WTT,WTV,WTW,WTY,WVA,WVC,WVD,WVE,WVF,WVG,WVH,WVI,WVK,WVL,WVM,WVN,WVP,WVQ,WVR,WVS,WVT,WVV,WVW,WVY,WWA,WWC,WWD,WWE,WWF,WWG,WWH,WWI,WWK,WWL,WWM,WWN,WWP,WWQ,WWR,WWS,WWT,WWV,WWW,WWY,WYA,WYC,WYD,WYE,WYF,WYG,WYH,WYI,WYK,WYL,WYM,WYN,WYP,WYQ,WYR,WYS,WYT,WYV,WYW,WYY,YAA,YAC,YAD,YAE,YAF,YAG,YAH,YAI,YAK,YAL,YAM,YAN,YAP,YAQ,YAR,YAS,YAT,YAV,YAW,YAY,YCA,YCC,YCD,YCE,YCF,YCG,YCH,YCI,YCK,YCL,YCM,YCN,YCP,YCQ,YCR,YCS,YCT,YCV,YCW,YCY,YDA,YDC,YDD,YDE,YDF,YDG,YDH,YDI,YDK,YDL,YDM,YDN,YDP,YDQ,YDR,YDS,YDT,YDV,YDW,YDY,YEA,YEC,YED,YEE,YEF,YEG,YEH,YEI,YEK,YEL,YEM,YEN,YEP,YEQ,YER,YES,YET,YEV,YEW,YEY,YFA,YFC,YFD,YFE,YFF,YFG,YFH,YFI,YFK,YFL,YFM,YFN,YFP,YFQ,YFR,YFS,YFT,YFV,YFW,YFY,YGA,YGC,YGD,YGE,YGF,YGG,YGH,YGI,YGK,YGL,YGM,YGN,YGP,YGQ,YGR,YGS,YGT,YGV,YGW,YGY,YHA,YHC,YHD,YHE,YHF,YHG,YHH,YHI,YHK,YHL,YHM,YHN,YHP,YHQ,YHR,YHS,YHT,YHV,YHW,YHY,YIA,YIC,YID,YIE,YIF,YIG,YIH,YII,YIK,YIL,YIM,YIN,YIP,YIQ,YIR,YIS,YIT,YIV,YIW,YIY,YKA,YKC,YKD,YKE,YKF,YKG,YKH,YKI,YKK,YKL,YKM,YKN,YKP,YKQ,YKR,YKS,YKT,YKV,YKW,YKY,YLA,YLC,YLD,YLE,YLF,YLG,YLH,YLI,YLK,YLL,YLM,YLN,YLP,YLQ,YLR,YLS,YLT,YLV,YLW,YLY,YMA,YMC,YMD,YME,YMF,YMG,YMH,YMI,YMK,YML,YMM,YMN,YMP,YMQ,YMR,YMS,YMT,YMV,YMW,YMY,YNA,YNC,YND,YNE,YNF,YNG,YNH,YNI,YNK,YNL,YNM,YNN,YNP,YNQ,YNR,YNS,YNT,YNV,YNW,YNY,YPA,YPC,YPD,YPE,YPF,YPG,YPH,YPI,YPK,YPL,YPM,YPN,YPP,YPQ,YPR,YPS,YPT,YPV,YPW,YPY,YQA,YQC,YQD,YQE,YQF,YQG,YQH,YQI,YQK,YQL,YQM,YQN,YQP,YQQ,YQR,YQS,YQT,YQV,YQW,YQY,YRA,YRC,YRD,YRE,YRF,YRG,YRH,YRI,YRK,YRL,YRM,YRN,YRP,YRQ,YRR,YRS,YRT,YRV,YRW,YRY,YSA,YSC,YSD,YSE,YSF,YSG,YSH,YSI,YSK,YSL,YSM,YSN,YSP,YSQ,YSR,YSS,YST,YSV,YSW,YSY,YTA,YTC,YTD,YTE,YTF,YTG,YTH,YTI,YTK,YTL,YTM,YTN,YTP,YTQ,YTR,YTS,YTT,YTV,YTW,YTY,YVA,YVC,YVD,YVE,YVF,YVG,YVH,YVI,YVK,YVL,YVM,YVN,YVP,YVQ,YVR,YVS,YVT,YVV,YVW,YVY,YWA,YWC,YWD,YWE,YWF,YWG,YWH,YWI,YWK,YWL,YWM,YWN,YWP,YWQ,YWR,YWS,YWT,YWV,YWW,YWY,YYA,YYC,YYD,YYE,YYF,YYG,YYH,YYI,YYK,YYL,YYM,YYN,YYP,YYQ,YYR,YYS,YYT,YYV,YYW,YYY,Xc1.A,Xc1.C,Xc1.D,Xc1.E,Xc1.F,Xc1.G,Xc1.H,Xc1.I,Xc1.K,Xc1.L,Xc1.M,Xc1.N,Xc1.P,Xc1.Q,Xc1.R,Xc1.S,Xc1.T,Xc1.V,Xc1.W,Xc1.Y,Xc2.lambda1,Xc2.lambda2,Xc2.lambda3,Xc2.lambda4,Xc2.lambda5,xXc2.lambda6,Xc2.lambda7,Xc2.lambda8,Xc2.lambda9,Xc2.lambda10,Xc2.lambda11,Xc2.lambda12,Xc2.lambda13,Xc2.lambda14,Xc2.lambda15,Xc2.lambda16,Xc2.lambda17,Xc2.lambda18,Xc2.lambda19,Xc2.lambda20,Xc2.lambda21,Xc2.lambda22,Xc2.lambda23,Xc2.lambda24,Xc2.lambda25,Xc2.lambda26,Xc2.lambda27,Xc2.lambda28,Xc2.lambda29
2. **Physicochemical based features:**
  **aliphatic,aromatic,postivecharge**,negativecharge,uncharge. Moran_CIDH920105.lag1,Moran_CIDH920105.lag2,Moran_CIDH920105.lag3,Moran_CIDH920105.lag4,Moran_CIDH920105.lag5,Moran_CIDH920105.lag6,Moran_CIDH920105.lag7,Moran_CIDH920105.lag8,Moran_CIDH920105.lag9,Moran_CIDH920105.lag10,Moran_CIDH920105.lag11,Moran_CIDH920105.lag12,Moran_CIDH920105.lag13,Moran_CIDH920105.lag14,Moran_CIDH920105.lag15,Moran_CIDH920105.lag16,Moran_CIDH920105.lag17,Moran_CIDH920105.lag18,Moran_CIDH920105.lag19,Moran_CIDH920105.lag20,Moran_CIDH920105.lag21,Moran_CIDH920105.lag22,Moran_CIDH920105.lag23,Moran_CIDH920105.lag24,Moran_CIDH920105.lag25,Moran_CIDH920105.lag26,Moran_CIDH920105.lag27,Moran_CIDH920105.lag28,Moran_CIDH920105.lag29,Moran_BHAR880101.lag1,Moran_BHAR880101.lag2,Moran_BHAR880101.lag3,Moran_BHAR880101.lag4,Moran_BHAR880101.lag5,Moran_BHAR880101.lag6,Moran_BHAR880101.lag7,Moran_BHAR880101.lag8,Moran_BHAR880101.lag9,Moran_BHAR880101.lag10,Moran_BHAR880101.lag11,Moran_BHAR880101.lag12,Moran_BHAR880101.lag13,Moran_BHAR880101.lag14,Moran_BHAR880101.lag15,Moran_BHAR880101.lag16,Moran_BHAR880101.lag17,Moran_BHAR880101.lag18,Moran_BHAR880101.lag19,Moran_BHAR880101.lag20,Moran_BHAR880101.lag21,Moran_BHAR880101.lag22,Moran_BHAR880101.lag23,Moran_BHAR880101.lag24,Moran_BHAR880101.lag25,Moran_BHAR880101.lag26,Moran_BHAR880101.lag27,Moran_BHAR880101.lag28,Moran_BHAR880101.lag29,Moran_CHAM820101.lag1,Moran_CHAM820101.lag2,Moran_CHAM820101.lag3,Moran_CHAM820101.lag4,Moran_CHAM820101.lag5,Moran_CHAM820101.lag6,Moran_CHAM820101.lag7,Moran_CHAM820101.lag8,Moran_CHAM820101.lag9,Moran_CHAM820101.lag10,Moran_CHAM820101.lag11,Moran_CHAM820101.lag12,Moran_CHAM820101.lag13,Moran_CHAM820101.lag14,Moran_CHAM820101.lag15,Moran_CHAM820101.lag16,Moran_CHAM820101.lag17,Moran_CHAM820101.lag18,Moran_CHAM820101.lag19,Moran_CHAM820101.lag20,Moran_CHAM820101.lag21,Moran_CHAM820101.lag22,Moran_CHAM820101.lag23,Moran_CHAM820101.lag24,Moran_CHAM820101.lag25,Moran_CHAM820101.lag26,Moran_CHAM820101.lag27,Moran_CHAM820101.lag28,Moran_CHAM820101.lag29,Moran_CHAM820102.lag1,Moran_CHAM820102.lag2,Moran_CHAM820102.lag3,Moran_CHAM820102.lag4,Moran_CHAM820102.lag5,Moran_CHAM820102.lag6,Moran_CHAM820102.lag7,Moran_CHAM820102.lag8,Moran_CHAM820102.lag9,Moran_CHAM820102.lag10,Moran_CHAM820102.lag11,Moran_CHAM820102.lag12,Moran_CHAM820102.lag13,Moran_CHAM820102.lag14,Moran_CHAM820102.lag15,Moran_CHAM820102.lag16,Moran_CHAM820102.lag17,Moran_CHAM820102.lag18,Moran_CHAM820102.lag19,Moran_CHAM820102.lag20,Moran_CHAM820102.lag21,Moran_CHAM820102.lag22,Moran_CHAM820102.lag23,Moran_CHAM820102.lag24,Moran_CHAM820102.lag25,Moran_CHAM820102.lag26,Moran_CHAM820102.lag27,Moran_CHAM820102.lag28,Moran_CHAM820102.lag29,Moran_CHOC760101.lag1,Moran_CHOC760101.lag2,Moran_CHOC760101.lag3,Moran_CHOC760101.lag4,Moran_CHOC760101.lag5,Moran_CHOC760101.lag6,Moran_CHOC760101.lag7,Moran_CHOC760101.lag8,Moran_CHOC760101.lag9,Moran_CHOC760101.lag10,Moran_CHOC760101.lag11,Moran_CHOC760101.lag12,Moran_CHOC760101.lag13,Moran_CHOC760101.lag14,Moran_CHOC760101.lag15,Moran_CHOC760101.lag16,Moran_CHOC760101.lag17,Moran_CHOC760101.lag18,Moran_CHOC760101.lag19,Moran_CHOC760101.lag20,Moran_CHOC760101.lag21,Moran_CHOC760101.lag22,Moran_CHOC760101.lag23,Moran_CHOC760101.lag24,Moran_CHOC760101.lag25,Moran_CHOC760101.lag26,Moran_CHOC760101.lag27,Moran_CHOC760101.lag28,Moran_CHOC760101.lag29,Moran_BIGC670101.lag1,Moran_BIGC670101.lag2,Moran_BIGC670101.lag3,Moran_BIGC670101.lag4,Moran_BIGC670101.lag5,Moran_BIGC670101.lag6,Moran_BIGC670101.lag7,Moran_BIGC670101.lag8,Moran_BIGC670101.lag9,Moran_BIGC670101.lag10,Moran_BIGC670101.lag11,Moran_BIGC670101.lag12,Moran_BIGC670101.lag13,Moran_BIGC670101.lag14,Moran_BIGC670101.lag15,Moran_BIGC670101.lag16,Moran_BIGC670101.lag17,Moran_BIGC670101.lag18,Moran_BIGC670101.lag19,Moran_BIGC670101.lag20,Moran_BIGC670101.lag21,Moran_BIGC670101.lag22,Moran_BIGC670101.lag23,Moran_BIGC670101.lag24,Moran_BIGC670101.lag25,Moran_BIGC670101.lag26,Moran_BIGC670101.lag27,Moran_BIGC670101.lag28,Moran_BIGC670101.lag29,Moran_CHAM810101.lag1,Moran_CHAM810101.lag2,Moran_CHAM810101.lag3,Moran_CHAM810101.lag4,Moran_CHAM810101.lag5,Moran_CHAM810101.lag6,Moran_CHAM810101.lag7,Moran_CHAM810101.lag8,Moran_CHAM810101.lag9,Moran_CHAM810101.lag10,Moran_CHAM810101.lag11,Moran_CHAM810101.lag12,Moran_CHAM810101.lag13,Moran_CHAM810101.lag14,Moran_CHAM810101.lag15,Moran_CHAM810101.lag16,Moran_CHAM810101.lag17,Moran_CHAM810101.lag18,Moran_CHAM810101.lag19,Moran_CHAM810101.lag20,Moran_CHAM810101.lag21,Moran_CHAM810101.lag22,Moran_CHAM810101.lag23,Moran_CHAM810101.lag24,Moran_CHAM810101.lag25,Moran_CHAM810101.lag26,Moran_CHAM810101.lag27,Moran_CHAM810101.lag28,Moran_CHAM810101.lag29,Moran_DAYM780201.lag1,Moran_DAYM780201.lag2,Moran_DAYM780201.lag3,Moran_DAYM780201.lag4,Moran_DAYM780201.lag5,Moran_DAYM780201.lag6,Moran_DAYM780201.lag7,Moran_DAYM780201.lag8,Moran_DAYM780201.lag9,Moran_DAYM780201.lag10,Moran_DAYM780201.lag11,Moran_DAYM780201.lag12,Moran_DAYM780201.lag13,Moran_DAYM780201.lag14,Moran_DAYM780201.lag15,Moran_DAYM780201.lag16,Moran_DAYM780201.lag17,Moran_DAYM780201.lag18,Moran_DAYM780201.lag19,Moran_DAYM780201.lag20,Moran_DAYM780201.lag21,Moran_DAYM780201.lag22,Moran_DAYM780201.lag23,Moran_DAYM780201.lag24,Moran_DAYM780201.lag25,Moran_DAYM780201.lag26,Moran_DAYM780201.lag27,Moran_DAYM780201.lag28,Moran_DAYM780201.lag29,hydrophobicity_PRAM900101.G1,hydrophobicity_PRAM900101.G2,hydrophobicity_PRAM900101.G3,hydrophobicity_ARGP820101.G1,hydrophobicity_ARGP820101.G2,hydrophobicity_ARGP820101.G3,hydrophobicity_ZIMJ680101.G1,hydrophobicity_ZIMJ680101.G2,hydrophobicity_ZIMJ680101.G3,hydrophobicity_PONP930101.G1,hydrophobicity_PONP930101.G2,hydrophobicity_PONP930101.G3,hydrophobicity_CASG920101.G1,hydrophobicity_CASG920101.G2,hydrophobicity_CASG920101.G3,hydrophobicity_ENGD860101.G1,hydrophobicity_ENGD860101.G2,hydrophobicity_ENGD860101.G3,hydrophobicity_FASG890101.G1,hydrophobicity_FASG890101.G2,hydrophobicity_FASG890101.G3,normwaalsvolume.G1,normwaalsvolume.G2,normwaalsvolume.G3,polarity.G1,polarity.G2,polarity.G3,polarizability.G1,polarizability.G2,polarizability.G3,charge.G1,charge.G2,charge.G3,secondarystruct.G1,secondarystruct.G2,secondarystruct.G3,solventaccess.G1,solventaccess.G2,solventaccess.G3,hydrophobicity_PRAM900101.1.residue0,hydrophobicity_PRAM900101.1.residue25,hydrophobicity_PRAM900101.1.residue50,hydrophobicity_PRAM900101.1.residue75,hydrophobicity_PRAM900101.1.residue100,hydrophobicity_PRAM900101.2.residue0,hydrophobicity_PRAM900101.2.residue25,hydrophobicity_PRAM900101.2.residue50,hydrophobicity_PRAM900101.2.residue75,hydrophobicity_PRAM900101.2.residue100,hydrophobicity_PRAM900101.3.residue0,hydrophobicity_PRAM900101.3.residue25,hydrophobicity_PRAM900101.3.residue50,hydrophobicity_PRAM900101.3.residue75,hydrophobicity_PRAM900101.3.residue100,hydrophobicity_ARGP820101.1.residue0,hydrophobicity_ARGP820101.1.residue25,hydrophobicity_ARGP820101.1.residue50,hydrophobicity_ARGP820101.1.residue75,hydrophobicity_ARGP820101.1.residue100,hydrophobicity_ARGP820101.2.residue0,hydrophobicity_ARGP820101.2.residue25,hydrophobicity_ARGP820101.2.residue50,hydrophobicity_ARGP820101.2.residue75,hydrophobicity_ARGP820101.2.residue100,hydrophobicity_ARGP820101.3.residue0,hydrophobicity_ARGP820101.3.residue25,hydrophobicity_ARGP820101.3.residue50,hydrophobicity_ARGP820101.3.residue75,hydrophobicity_ARGP820101.3.residue100,hydrophobicity_ZIMJ680101.1.residue0,hydrophobicity_ZIMJ680101.1.residue25,hydrophobicity_ZIMJ680101.1.residue50,hydrophobicity_ZIMJ680101.1.residue75,hydrophobicity_ZIMJ680101.1.residue100,hydrophobicity_ZIMJ680101.2.residue0,hydrophobicity_ZIMJ680101.2.residue25,hydrophobicity_ZIMJ680101.2.residue50,hydrophobicity_ZIMJ680101.2.residue75,hydrophobicity_ZIMJ680101.2.residue100,hydrophobicity_ZIMJ680101.3.residue0,hydrophobicity_ZIMJ680101.3.residue25,hydrophobicity_ZIMJ680101.3.residue50,hydrophobicity_ZIMJ680101.3.residue75,hydrophobicity_ZIMJ680101.3.residue100,hydrophobicity_PONP930101.1.residue0,hydrophobicity_PONP930101.1.residue25,hydrophobicity_PONP930101.1.residue50,hydrophobicity_PONP930101.1.residue75,hydrophobicity_PONP930101.1.residue100,hydrophobicity_PONP930101.2.residue0,hydrophobicity_PONP930101.2.residue25,hydrophobicity_PONP930101.2.residue50,hydrophobicity_PONP930101.2.residue75,hydrophobicity_PONP930101.2.residue100,hydrophobicity_PONP930101.3.residue0,hydrophobicity_PONP930101.3.residue25,hydrophobicity_PONP930101.3.residue50,hydrophobicity_PONP930101.3.residue75,hydrophobicity_PONP930101.3.residue100,hydrophobicity_CASG920101.1.residue0,hydrophobicity_CASG920101.1.residue25,hydrophobicity_CASG920101.1.residue50,hydrophobicity_CASG920101.1.residue75,hydrophobicity_CASG920101.1.residue100,hydrophobicity_CASG920101.2.residue0,hydrophobicity_CASG920101.2.residue25,hydrophobicity_CASG920101.2.residue50,hydrophobicity_CASG920101.2.residue75,hydrophobicity_CASG920101.2.residue100,hydrophobicity_CASG920101.3.residue0,hydrophobicity_CASG920101.3.residue25,hydrophobicity_CASG920101.3.residue50,hydrophobicity_CASG920101.3.residue75,hydrophobicity_CASG920101.3.residue100,hydrophobicity_ENGD860101.1.residue0,hydrophobicity_ENGD860101.1.residue25,hydrophobicity_ENGD860101.1.residue50,hydrophobicity_ENGD860101.1.residue75,hydrophobicity_ENGD860101.1.residue100,hydrophobicity_ENGD860101.2.residue0,hydrophobicity_ENGD860101.2.residue25,hydrophobicity_ENGD860101.2.residue50,hydrophobicity_ENGD860101.2.residue75,hydrophobicity_ENGD860101.2.residue100,hydrophobicity_ENGD860101.3.residue0,hydrophobicity_ENGD860101.3.residue25,hydrophobicity_ENGD860101.3.residue50,hydrophobicity_ENGD860101.3.residue75,hydrophobicity_ENGD860101.3.residue100,hydrophobicity_FASG890101.1.residue0,hydrophobicity_FASG890101.1.residue25,hydrophobicity_FASG890101.1.residue50,hydrophobicity_FASG890101.1.residue75,hydrophobicity_FASG890101.1.residue100,hydrophobicity_FASG890101.2.residue0,hydrophobicity_FASG890101.2.residue25,hydrophobicity_FASG890101.2.residue50,hydrophobicity_FASG890101.2.residue75,hydrophobicity_FASG890101.2.residue100,hydrophobicity_FASG890101.3.residue0,hydrophobicity_FASG890101.3.residue25,hydrophobicity_FASG890101.3.residue50,hydrophobicity_FASG890101.3.residue75,hydrophobicity_FASG890101.3.residue100,normwaalsvolume.1.residue0,normwaalsvolume.1.residue25,normwaalsvolume.1.residue50,normwaalsvolume.1.residue75,normwaalsvolume.1.residue100,normwaalsvolume.2.residue0,normwaalsvolume.2.residue25,normwaalsvolume.2.residue50,normwaalsvolume.2.residue75,normwaalsvolume.2.residue100,normwaalsvolume.3.residue0,normwaalsvolume.3.residue25,normwaalsvolume.3.residue50,normwaalsvolume.3.residue75,normwaalsvolume.3.residue100,polarity.1.residue0,polarity.1.residue25,polarity.1.residue50,polarity.1.residue75,polarity.1.residue100,polarity.2.residue0,polarity.2.residue25,polarity.2.residue50,polarity.2.residue75,polarity.2.residue100,polarity.3.residue0,polarity.3.xresidue25,polarity.3.residue50,polarity.3.residue75,polarity.3.residue100,polarizability.1.residue0,polarizability.1.residue25,polarizability.1.residue50,polarizability.1.residue75,polarizability.1.residue100,polarizability.2.residue0,polarizability.2.residue25,polarizability.2.residue50,polarizability.2.residue75,polarizability.2.residue100,polarizability.3.residue0,polarizability.3.residue25,polarizability.3.residue50,polarizability.3.residue75,polarizability.3.residue100,charge.1.residue0,charge.1.residue25,charge.1.residue50,charge.1.residue75,charge.1.residue100,charge.2.residue0,charge.2.residue25,charge.2.residue50,charge.2.residue75,charge.2.residue100,charge.3.residue0,charge.3.residue25,charge.3.residue50,charge.3.residue75,charge.3.residue100,secondarystruct.1.residue0,secondarystruct.1.residue25,secondarystruct.1.residue50,secondarystruct.1.residue75,secondarystruct.1.residue100,secondarystruct.2.residue0,secondarystruct.2.residue25,secondarystruct.2.residue50,secondarystruct.2.residue75,secondarystruct.2.residue100,secondarystruct.3.residue0,secondarystruct.3.residue25,secondarystruct.3.residue50,secondarystruct.3.residue75,secondarystruct.3.residue100,solventaccess.1.residue0,solventaccess.1.residue25,solventaccess.1.residue50,solventaccess.1.residue75,solventaccess.1.residue100,solventaccess.2.residue0,solventaccess.2.residue25,solventaccess.2.residue50,solventaccess.2.residue75,solventaccess.2.residue100,solventaccess.3.residue0,solventaccess.3.residue25,solventaccess.3.residue50,solventaccess.3.residue75,solventaccess.3.residue100,hydrophobicity_PRAM900101.Tr1221,hydrophobicity_PRAM900101.Tr1331,hydrophobicity_PRAM900101.Tr2332,hydrophobicity_ARGP820101.Tr1221,hydrophobicity_ARGP820101.Tr1331,hydrophobicity_ARGP820101.Tr2332,hydrophobicity_ZIMJ680101.Tr1221,hydrophobicity_ZIMJ680101.Tr1331,hydrophobicity_ZIMJ680101.Tr2332,hydrophobicity_PONP930101.Tr1221,hydrophobicity_PONP930101.Tr1331,hydrophobicity_PONP930101.Tr2332,hydrophobicity_CASG920101.Tr1221,hydrophobicity_CASG920101.Tr1331,hydrophobicity_CASG920101.Tr2332,hydrophobicity_ENGD860101.Tr1221,hydrophobicity_ENGD860101.Tr1331,hydrophobicity_ENGD860101.Tr2332,hydrophobicity_FASG890101.Tr1221,hydrophobicity_FASG890101.Tr1331,hydrophobicity_FASG890101.Tr2332,normwaalsvolume.Tr1221,normwaalsvolume.Tr1331,normwaalsvolume.Tr2332,polarity.Tr1221,polarity.Tr1331,polarity.Tr2332,polarizability.Tr1221,polarizability.Tr1331,polarizability.Tr2332,charge.Tr1221,charge.Tr1331,charge.Tr2332,secondarystruct.Tr1221,secondarystruct.Tr1331,secondarystruct.Tr2332,solventaccess.Tr1221,solventaccess.Tr1331,solventaccess.Tr2332,g1.g1.g1,g1.g1.g2,g1.g1.g3,g1.g1.g4,g1.g1.g5,g1.g1.g6,g1.g1.g7,g1.g2.g1,g1.g2.g2,g1.g2.g3,g1.g2.g4,g1.g2.g5,g1.g2.g6,g1.g2.g7,g1.g3.g1,g1.g3.g2,g1.g3.g3,g1.g3.g4,g1.g3.g5,g1.g3.g6,g1.g3.g7,g1.g4.g1,g1.g4.g2,g1.g4.g3,g1.g4.g4,g1.g4.g5,g1.g4.g6,g1.g4.g7,g1.g5.g1,g1.g5.g2,g1.g5.g3,g1.g5.g4,g1.g5.g5,g1.g5.g6,g1.g5.g7,g1.g6.g1,g1.g6.g2,g1.g6.g3,g1.g6.g4,g1.g6.g5,g1.g6.g6,g1.g6.g7,g1.g7.g1,g1.g7.g2,g1.g7.g3,g1.g7.g4,g1.g7.g5,g1.g7.g6,g1.g7.g7,g2.g1.g1,g2.g1.g2,g2.g1.g3,g2.g1.g4,g2.g1.g5,g2.g1.g6,g2.g1.g7,g2.g2.g1,g2.g2.g2,g2.g2.g3,g2.g2.g4,g2.g2.g5,g2.g2.g6,g2.g2.g7,g2.g3.g1,g2.g3.g2,g2.g3.g3,g2.g3.g4,g2.g3.g5,g2.g3.g6,g2.g3.g7,g2.g4.g1,g2.g4.g2,g2.g4.g3,g2.g4.g4,g2.g4.g5,g2.g4.g6,g2.g4.g7,g2.g5.g1,g2.g5.g2,g2.g5.g3,g2.g5.g4,g2.g5.g5,g2.g5.g6,g2.g5.g7,g2.g6.g1,g2.g6.g2,g2.g6.g3,g2.g6.g4,g2.g6.g5,g2.g6.g6,g2.g6.g7,g2.g7.g1,g2.g7.g2,g2.g7.g3,g2.g7.g4,g2.g7.g5,g2.g7.g6,g2.g7.g7,g3.g1.g1,g3.g1.g2,g3.g1.g3,g3.g1.g4,g3.g1.g5,g3.g1.g6,g3.g1.g7,g3.g2.g1,g3.g2.g2,g3.g2.g3,g3.g2.g4,g3.g2.g5,g3.g2.g6,g3.g2.g7,g3.g3.g1,g3.g3.g2,g3.g3.g3,g3.g3.g4,g3.g3.g5,g3.g3.g6,g3.g3.g7,g3.g4.g1,g3.g4.g2,g3.g4.g3,g3.g4.g4,g3.g4.g5,g3.g4.g6,g3.g4.g7,g3.g5.g1,g3.g5.g2,g3.g5.g3,g3.g5.g4,g3.g5.g5,g3.g5.g6,g3.g5.g7,g3.g6.g1,g3.g6.g2,g3.g6.g3,g3.g6.g4,g3.g6.g5,g3.g6.g6,g3.g6.g7,g3.g7.g1,g3.g7.g2,g3.g7.g3,g3.g7.g4,g3.g7.g5,g3.g7.g6,g3.g7.g7,g4.g1.g1,g4.g1.g2,g4.g1.g3,g4.g1.g4,g4.g1.g5,g4.g1.g6,g4.g1.g7,g4.g2.g1,g4.g2.g2,g4.g2.g3,g4.g2.g4,g4.g2.g5,g4.g2.g6,g4.g2.g7,g4.g3.g1,g4.g3.g2,g4.g3.g3,g4.g3.g4,g4.g3.g5,g4.g3.g6,g4.g3.g7,g4.g4.g1,g4.g4.g2,g4.g4.g3,g4.g4.g4,g4.g4.g5,g4.g4.g6,g4.g4.g7,g4.g5.g1,g4.g5.g2,g4.g5.g3,g4.g5.g4,g4.g5.g5,g4.g5.g6,g4.g5.g7,g4.g6.g1,g4.g6.g2,g4.g6.g3,g4.g6.g4,g4.g6.g5,g4.g6.g6,g4.g6.g7,g4.g7.g1,g4.g7.g2,g4.g7.g3,g4.g7.g4,g4.g7.g5,g4.g7.g6,g4.g7.g7,g5.g1.g1,g5.g1.g2,g5.g1.g3,g5.g1.g4,g5.g1.g5,g5.g1.g6,g5.g1.g7,g5.g2.g1,g5.g2.g2,g5.g2.g3,g5.g2.g4,g5.g2.g5,g5.g2.g6,g5.g2.g7,g5.g3.g1,g5.g3.g2,g5.g3.g3,g5.g3.g4,g5.g3.g5,g5.g3.g6,g5.g3.g7,g5.g4.g1,g5.g4.g2,g5.g4.g3,g5.g4.g4,g5.g4.g5,g5.g4.g6,g5.g4.g7,g5.g5.g1,g5.g5.g2,g5.g5.g3,g5.g5.g4,g5.g5.g5,g5.g5.g6,g5.g5.g7,g5.g6.g1,g5.g6.g2,g5.g6.g3,g5.g6.g4,g5.g6.g5,g5.g6.g6,g5.g6.g7,g5.g7.g1,g5.g7.g2,g5.g7.g3,g5.g7.g4,g5.g7.g5,g5.g7.g6,g5.g7.g7,g6.g1.g1,g6.g1.g2,g6.g1.g3,g6.g1.g4,g6.g1.g5,g6.g1.g6,g6.g1.g7,g6.g2.g1,g6.g2.g2,g6.g2.g3,g6.g2.g4,g6.g2.g5,g6.g2.g6,g6.g2.g7,g6.g3.g1,g6.g3.g2,g6.g3.g3,g6.g3.g4,g6.g3.g5,g6.g3.g6,g6.g3.g7,g6.g4.g1,g6.g4.g2,g6.g4.g3,g6.g4.g4,g6.g4.g5,g6.g4.g6,g6.g4.g7,g6.g5.g1,g6.g5.g2,g6.g5.g3,g6.g5.g4,g6.g5.g5,g6.g5.g6,g6.g5.g7,g6.g6.g1,g6.g6.g2,g6.g6.g3,g6.g6.g4,g6.g6.g5,g6.g6.g6,g6.g6.g7,g6.g7.g1,g6.g7.g2,g6.g7.g3,g6.g7.g4,g6.g7.g5,g6.g7.g6,g6.g7.g7,g7.g1.g1,g7.g1.g2,g7.g1.g3,g7.g1.g4,g7.g1.g5,g7.g1.g6,g7.g1.g7,g7.g2.g1,g7.g2.g2,g7.g2.g3,g7.g2.g4,g7.g2.g5,g7.g2.g6,g7.g2.g7,g7.g3.g1,g7.g3.g2,g7.g3.g3,g7.g3.g4,g7.g3.g5,g7.g3.g6,g7.g3.g7,g7.g4.g1,g7.g4.g2,g7.g4.g3,g7.g4.g4,g7.g4.g5,g7.g4.g6,g7.g4.g7,g7.g5.g1,g7.g5.g2,g7.g5.g3,g7.g5.g4,g7.g5.g5,g7.g5.g6,g7.g5.g7,g7.g6.g1,g7.g6.g2,g7.g6.g3,g7.g6.g4,g7.g6.g5,g7.g6.g6,g7.g6.g7,g7.g7.g1,g7.g7.g2,g7.g7.g3,g7.g7.g4,g7.g7.g5,g7.g7.g6,g7.g7.g7,mol_wt,isoelectric_pt, gravy, instability_index, helix, sheet, turn, cysteines, cystines, Length
3. **Annotation based features:**

**Table 1:** Summary of different feature groups and their descriptors [5].

S. No	Feature Group	Feature Name	Python Package used	Number of descriptor values
1.	Sequence-based	Protein Length	Biopython	1
2.	Sequence-based	Amino acid composition	ifeature	20
3.	Sequence-based	Dipeptide composition	Ifeature	400
4.	Sequence-based	Tripeptide composition	ifeature	8000
5.	Sequence-based	Pseudo amino acid composition	ifeature	49
6.	Subsequence-based	Motif count	Biopython	541
7.	Physicochemical-based	Molecular weight	Biopython	1
8.	Physicochemical-based	Instability index	Biopython	1
9.	Physicochemical-based	Isoelectric point	Biopython	1
10.	Physicochemical-based	GRAVY	Biopython	1
11.	Physicochemical-based	Extinction Coefficient	Biopython	2
12.	Physicochemical-based	Secondary structure fraction	Biopython	3
13.	Physicochemical-based	Grouped amino acid composition	ifeature	5
14.	Physicochemical-based	Moran autocorrelation	ifeature	232
15.	Physicochemical-based	Composition, Transition and Distribution	ifeature	273
16.	Physicochemical-based	Conjoint Triad	ifeature	343
17.	Annotation-based	Annotation based features (subcellular localisation, binding preference and presence of transmembrane region)	urllib (web-scrapping)	17
			TOTAL	9890

Transmembrane, DNA Binding, Metal Binding, Nucleotide Binding, cytoplasm, membrane, cell wall, secreted, periplasm, cell surface, cell envelope, chlorosome, cellular thylakoid membrane, cellular cromatopore membrane, single-pass membrane protein, multi-pass membrane protein, peripheral membrane protein.

4. **Subsequence based features (Motifs): Dataset contains count of these motifs** [8] **for corresponding protein**.
  G-{A}-{KGR}-x(2)-[LIVMFTAP]-{R}-x-[AGC]-C-[STA](2)-[STAG]-x(2)-{LI}-[LIVMF],G-[AV]-F-[STA]-x-R-[SA]-x(2)-R-P-N,[IVT]-[LIVMC]-[IVT]-[HS]-D-[SGAV]-[AV]-R,[LIVMF]-[LIVMSTA]-x-[LIVMFYC]-[FYWSTHE]-x(2)-[FYWGTN]-C-[GATPLVE]-[PHYWSTA]-C-{I}-x-{A}-x(3)-[LIVMFYWT],[LIVTMS]-[LIVP]-[LIV]-[KQ]-x-[ND]-Q-[INV]-[GA]-[ST]-[LIVM]-[STL]-[DERKAQG]-[STA],"[IL]-[GA]-x(2)-[LIVMF]-[SGADENK]-x(0,1)-[KR]-x-H-[STPA]-[STAV]-[LIVM]-x(2)-[SGAMN]-x(3)-[LIVM]",[LIV]-[LIVFY]-[FY]-x-[ST]-{V}-x-[AGC]-x-T-{P}-x(2)-A-{L}-x-[LIV],"[HQ]-[IVT]-x-[LIVFY]-x-[IV]-x(4)-{E}-[STA]-x(2)-F-[YM]-x(2,3)-[LMF]-G-[LMF]","[LIVMA]-[AG]-[IVT]-[LIVMFY]-[AG]-x-G-[NHKRQGSAC]-[LIV]-G-x(13,14)-[LIVMFT]-{A}-x-[FYWCTH]-[DNSTK]",Y-[CSAM]-x(2)-[VSG]-A-[GSA]-[LIVAT]-[IV]-G-x(2)-[LMSC]-x(2)-[LIV],"[GSTAI]-[SANQCVIT]-D-x-K-[GSACN]-x(1,2)-[LIVMA]-x(2)-[LIVMFY]-x(12,17)-[LIVM]-x-[LIVMF]-[LIVMSTAGC]-[LIVMFA]-x(2)-[DNGM]-E-E-x(0,1)-[GSTNE]","C-x(2,4)-C-x(3)-[LIVMFYWC]-x(8)-H-x(3,5)-H″,[GW]-x-[DNIE]-x-H-H-x(2)-E−[STAGC]-x-[VMFYHS]-K,"[FWV]-x(0,1)-[LIVM]-D-P-[LIVM]-D-[SG]-[ST]-x(2)-[FYA]-x(0,1)-[HKRNSTY]","[LIVM]-x(2)-[GSACIVM]-x-[LIV]-[GTIV]-[STP]-C-x(0,1)-T-N-[GSTANI]-x(4)-[LIVMA]",[YA]-[GLIVMSTAC]-D-T-D-[SG]-[LIVMFTC]-{LA}-[LIVMSTAC],[LYGSTANEQ]-x(3)-[GSTAENQ]-x-[PGE]-R-x-[LIVFYWA]-x-[LIVMFTA]-[STAGNQ]-[LIVMFYGTA]-x-[LIVMFYWGTADQ]-x-F>,E-x(5)-[GND]-x-[SAG]-x(2)-[IV]-x-[DE]-[LIV]-x(2)-[ST]-G-x-T-[LMI],[RH]-G-x(2)-P-x-G(3)-x-[LIV],[GDC]-x(2)-[NSTAVY]-x(2)-[IV]-[GSTA]-x(2)-[LIVMFYWCT]-x-[LIVMFYWCR]-x(3)-[NST]-[LIVM]-x(2)-{T}-x(2)-[NRHSA]-[LIVMSTA]-x(2)-[KR],P-[LIVM]-x-[FYL]-[LIVMAT]-[GS]-{Q}-[GS]-[EQ]-x-{K}-x(2)-[LIVMF],"F-[GSADEI]-x-[LVAQ]-A-x(3)-[ST]-x(3,4)-[STQ]-x(3,5)-[GER]-G-x-[LIVM]-[GS]",[LIVMFYC]-{A}-[HY]-x-D-[LIVMFY]-[RSTAC]-{D}-{PF}-N-[LIVMFYC](3),W-x(2)-[LIVM]-D-[VFY]-[LIVM](3)-D-x-P-P-G-T-[GS]-D,"K–P-[LIVMFYA]-x(3,5)-[NPAT]-[GA]-[GSTAN]-[GA]-x-H-x(3)-S″,[FYLVA]-x-{GVEP}-{DILV}-G-[QE]-{LPYG}-C-[LIVMGSTANC]-[AGCN]-{HE}-[GSTADNEKR],[LIVMF]-x-[LIVMFAG]-{T}-x-[STAGI]-H-D-[STANQ]-{V}-[LIVM]-x(2)-[LIVMFY]-x(2)-[STA],D-R-G-H-[QLIM]-x(3)-[AG],[LIVM]-E-x-E−[LIVM]-G-x(2)-[GM]-[GSTA]-x-E,R-G-x(2)-E-N-x-N-G-[LIVM](2)-R-[QE]-[LIVMFY](2)-P-K,"[PALF]-x(2,3)-[LIV]-x(3)-[LIVM]-[STAC]-[STV]-x-[GANK]-G-x-T-x(2)-[AG]-[LIV]-x(2)-[LMF]-[DENQK]",D-P-x-F-[LIVMFYW]-x(2)-H-x(3)-D,"[KR]-[LIVM](2)-[GASL]-x-[GT]-x-[LIVMA]-x(2,5)-[LIVMF]-x-[LIVMF]-x(3,4)-[LIVMFCA]-[ST]-x(2)-A-x(3)-[LIVM]-x(3)-G″,[LIVM]-x(2)-P-x(2)-[FY]-x(4)-C-x-G-x-C,[LIVMFTAR]-[LIVMF]-x-D-x-K-x(2)-D-[IV]-[ADGP]-x-T-[CLIVMNTA],P-[LIVM]-x-[LIVM]-x(2)-[LIVM]-A-x(2)-[LIVMFT]-x(2)-[HS]-x-S-T-[LIVM]-S-R,"[FYKH]-G-[FL]-[IL]-x(6,7)-[DER]-[LIVM]-[FQ]-x-H-x-[STKR]-x-[LIVMFYC]",H–Y-x-[GT]-D-[LIVMAF]-[DNSH]-x-P-x-H-[PA]-x-N,[ST]-[LIVMFY]-D-[LIVM]-D-x(3)-[PAQ]-x(3)-P-[GSA]-x(7)-G,[EQ]-{LNYH}-x-[ATV]-[FY]-{LDAM}-{T}-W-{PG}-N,"[PS]-x-[SAC]-x-[LIVMFY](2)-[QN]-x(2)-N-P-x(4)-[TA]-x(9,11)-[KRD]-x-[LIV]-[GN]-x-C″,R-R-T-[IV]-[ATN]-K-Y-R,[LIVF]-x(2)-D-x-[NH]-x(7)-[ACL]-x(6)-[LIVMF]-x(7)-[LIVM]-E−[DENQ]-P,[LIV]-[GALMY]-[LIVMF]-{Q}-[GSA]-H-x-D-[TV]-[STAV],"[YWG]-[LIVFYWTA](2)-[VGS]-H-[LNP]-x-V-x(44,47)-H-H″,H-x(3)-[GA]-[LIVMT]-R-[HF]-[LIVMF]-x-[FYWM]-D-x-[GVA],[FY]-{L}-C-{PGAD}-[VA]-{LC}-H,G-x-[LIVM](2)-x-R-Q-R-G-x(5)-G,"K-[STNV]-{F}-x-[GSAM]-[SAILV]-x-[KRA]-R-[IVFY]-x(14,16)-[GSANQKR]-H″,"[LIVMFY]-[LIVMC]-x-E−[LIVMFYC]-K-[KRSPQV]-[STAHKRYC]-S-P-[STRK]-x(3,7)-[LIVMFYST]",[DENKS]-x-[FLIV]-x(2)-[GSTC]-x-P-C-x-{V}-[FYWLIM]-S,[GS]-x-[LIVMFA]-x(2)-[AS]-[DNEQASH]-[GNEKT]-G-[STIM]-[LIVMFY](3)-[DE]-[EK]-[LIVM],"C-x(2)-C-x(3,5)-[STACD]-x(4)-C-x-[LIVFQ]-C-x(4)-[RD]-[NQDS]",[LIV]-{LA}-[EDQ]-[FYWKR]-V-{VF}-[LIVF]-G-[LF]-[ST],G-I-[GR]-P-x-Y-x(2)-K-x(2)-R,[GSTEQKRV]-Q-[LIVT]-[VAF]-[SAGQ]-G-{DG}-[LIVMNK]-{TK}-x-[LIVMFY]-{S}-[LIVMFYA]-[DENQKRHSIV],D-[LIVMA]-P-G-[LIVM](2)-[DEYPKQV]-[GN]-A-x(2)-G-x-G,[LIVM]-x(2)-[LIVM]-[STAVC]-[GE]-[QV]-x(2)-[LIVMA]-x-[STC]-x-[STAG]-[KRH]-x-[STA],[IL]-x-[STV]-[GT]-x(2)-[KR]-x-[KRAF]-x(6)-[DE]-x-[LIMV]-[LIVMT]-[TE]-x-[STAG]-[KR],"[LIVM]-G-x(3)-Q-x(2,3)-[ND]-[IFL]-x-[RE]-D-[LIVMFY]-x(2)-[DE]-x(4,7)-R-x-[FY]-x-P″,"[LIVMFY]-x-[LIVM]-[STAG]-G-T-[NK]-G-K-x-[STG]-x(4)-{A}-x-{EAD}-[LIVM](2)-x(3,4)-[GSKQT]",F-[GSTV]-P-R-L-[G>],R-C-[LIVM]-x-C-x-R-C-[LIVMT]-x-[LMFY],G-[GAV]-S-[GS](2)-G-x-[GSAE]-[GSAVYCT]-x-[LIVMT]-[GSA]-x(6)-[GSAT]-x-[GA]-x-[DE]-x-[GA]-x-S-[LIVM]-R-x-P-[GSACTL],[GR]-C-[IV]-G-R-[ILS]-x-W,[LIV]-x(3)-C-[NDP]-[LIVMF]-[DNQRS]-C-x-[FYM]-C,"[LIVMSTAG]-[LIVMFSAG]-{SH}-{RDE}-[LIVMSA]-[DE]-{TD}-[LIVMFYWA]-G-R-[RK]-x(4,6)-[GSTA]",[SAV]-[IVW]-[LVA]-[LIV]-G-[PNS]-G-L-[GP]-x-[DENQT],D-G-[PD]-S-A-[GS]-[LIVMCA]-[TA]-[LIVM],R-P-C-x(11)-C-V-S,[LIV]-[LIVMFYWGA](2)-[DNEQG]-[LIVMGST]-{SENR}-N-E−[PV]-[RHDNSTLIVFY],[FY]-{GL}-x-[LIVMA]-{IP}-x(2)-[FYWHNT]-[DENQSA]-x-L-x-[DN]-x(3)-[KR]-{F}-{P}-[FYI],K-[LIVM]-x-R-D-x(3)-R-G-x-[ST]-x-E,[LIVM]-[ST]-A-[STAG]-H-C,R-x(2)-[GSAV]-K-x(3)-[LIVMFY]-[AGQ]-x(2)-Y-x(2)-[GS]-x(3)-[LIVMA],D-x-[LI]-x(4)-G-x-D-x-[LI]-x-G-G-x(3)-D,Q-[LV]-[NT]-[FY]-[ST]-x(2)-W,P-x(3)-[LIVM](2)-x-G-x-C-[LIVMF](2)-K,F-x-[EK]-x-S-[GT]-R-T,"W-x(2)-[LIVF]-x(6,7)-G-[LIVM]-[FYRA]-[NH]-x(3)-[STAQLIVM]-[ASC]-x(2)-[PA]","C–P-x(0,1)-[ST]-N-[ILV]-G-T″,H-[GSA]-x-[LVCYT]-H-[LAI]-[LIMSANQVF]-G-[FYWMH]-x-[HD],[GSDNA]-W-T-[LIVM]-x-[FY]-W-x-W-W,"[RP]-x(0,1)-C-x(11,12)-[LIVMF]-{L}-[LIVMF]-[SC]-[RG]-x-{D}-{PK}-[RN]",[KNQS]-[PSTLNH]-{D}-{F}-[LIVMFA]-[KRGSADN]-x-[LIVYSTA]-[KR]-[KRHQS]-[DESTANQRL]-[LIV]-A-[KRCQVT]-[LIVMA],"[IVRLP]-[DYN]-[YLF]-x(2,3)-[LIVMTPFS]-x(2)-[LIVM]-x(2)-[FYTS]-[LIVMT]-[STNQG]-[DERPN]-x(1,2)-[GYAH]-[KCR]-[LIVM]-x(3)-[RHG]-[LIVMASR]","L-R-x(2)-[TS]-[GSDNQ]-x-[GSA]-[LIVMF]-x(0,1)-[DENKAC]-x-K-[KRNEQS]-[AV]-L″,"[CA]-[DE]-[LIVM](2)-[NQV]-[GTA]-D-[GA]-[SG]-x(2,3)-[TAVLC]-[AT]",[STAGN]-{E}-[STAG]-[LIVMF]-R-L-{LP}-[SAGV]-N-[LIVMT],D-[SGDN]-D-[PE]-[LIVMF]-D-[LIVMGAC],[GSTALIVN]-{PCHR}-{KND}-H-E−[LIVMFYW]-{DEHRKP}-H-{EKPC}-[LIVMFYWGSPQ],[LI]-[IVCAP]-D-x-K-[LIFY]-E−[FI]-G,[KRG]-[KR]-x-[GSAC]-[KRQVA]-[LIVMK]-[WY]-[LIVM]-[KRN]-[LIVM]-[LFY]-[APK],[LIVMN]-[KR]-G-N-H-E,[STVN]-G-C-x(3)-C-x(6)-[DE]-[LIVMF]-[GAT]-[LIVMF],[KRQSEAT]-[GS]-x-R-H-x(2)-[GSNHKLCD]-x(2)-[LIVMCT]-[RNH]-G-Q,[LIVMA]-G-[EQ]-H-G-[DN]-[ST],H-G-[STM]-x-[VIC]-[STAGC]-[GS]-x-[LIVMA]-[STAGCLV]-[SAGM],"[DENG]-{A}-[DENQGSTARK]-x(0,2)-[DENQARK]-[LIVFY]-{CP}-G-{C}-W-[FYWLRH]-{D}-[LIVMTA]",[LIVMACST]-H-P-[LIVM]-x-[KRQV]-[LIVMF](2)-x-[AP]-H,"[KR]-x(2)-[ST]-G-[GAR]-x(5,6)-[KRHSA]-x-[KRT]-x-[KR]-x-[EA]-[LIMPA]-G″,[PK]-x-[LIVMFY]-x-[LIVMFY]-x(2)-{E}-x-H-[STAG]-x-E-x-[LIVM]-[STAG]-{L}-x(5)-[LIVMFYTA],[DKG]-x(2)-[FLV]-[STKD]-x(5)-C-[LMNQ]-[GA]-x-C-x(2)-[GA]-P,K-[LIVM]-x(5)-[LIVMA]-D-[RK]-[DN]-[LI]-Y,[DE]-[LIVMF](2)-[HEQS]-x-G-x-[LIVMFA]-G-L-[LIVMFYE]-x-[GSAM]-[LIVMAP],T-[LIVMFYW]-[STAG]-K-[SAG]-[LIVMFYWR]-[SAG]-{ENKR}-{TNDR}-[SAG],[LIVM]-[STAG]-x-[LIVM]-[DENQRHSTA]-G-x(3)-[AG](3)-x(4)-[LIVMST]-x-[CSTA]-[DQHP]-[LIVMFYA],[GS]-{PR}-S-M-{RS}-[PS]-[AT]-[LF],"E-x(2)-[ERK]-E-x-C-x(6)-[EDR]-x(10,11)-[FYA]-[YW]",A-[AS]-{L}-[DEQ]-E−{A}-{Q}-{R}-x-G-G-[GA],G-x(2)-[GNF]-x(4)-[VAI]-x(2)-G-[FY]-x(2)-[NH]-[FYWL]-L-x(5)-[GA]-x(3)-[STNG],[ASL]-[FY]-S-G-G-[LV]-D-T-[ST],"G-[YV]-x-[ST]-x(2)-[IVAS]-G-K-x(0,1)-[FYWMK]-[HL]",Y-R-N-x-W-[NS]-E−[LIVM]-R-T-L-H-F-x-G,E−[ST]-C-G-x-C-x-P-C-R-x-G,[GN]-[AS]-G-D-Q-G-x(3)-G-[FYHG],T-G-x-P-[LIVM](2)-D-A-x-M-[RA]-x-[LIVM],"[GDEN]-D-x-[IV]-x-[IV]-[LIVMA]-x-G-x(2)-[KRA]-[GNQK]-x(2,3)-[GA]-x-[IV]",G-[LIVMFYKRSAQT]-[LIVMAGPF]-[QAM]-x-[LIVMFYCA]-x-D-[AGIM]-[LIVMFTA]-[KS]-[LVMYSTI]-[LIVMFYGA]-x-[KRE]-[EQG],"[KRQ]-[LIVMA]-x(2)-[GSTALIV]-{FYWPGDN}-x(2)-[LIVMSA]-x(4,9)-[LIVMF]-x-{PLH}-[LIVMSTA]-[GSTACIL]-{GPK}-{F}-x-[GANQRF]-[LIVMFY]-x(4,5)-[LFY]-x(3)-[FYIVA]-{FYWHCM}-{PGVI}-x(2)-[GSADENQKR]-x-[NSTAPKL]-[PARL]",D-x(3)-G-[LIVMF]-x(6)-[STAV]-[LIVMFYW]-[PT]-x-[STAV]-x(2)-[QR]-x-C-x(2)-H,"[FYWL]-D-G-S-S-x(6,8)-[DENQSTAK]-[SA]-[DE]-x(2)-[LIVMFY]",[DGH]-[IVSAC]-T-[ST]-N-P-[STA]-[LIVMF](2),[LIVMFY]-{G}-[LIVMFYAC]-[DNQ]-[RKHQS]-[PST]-F-[LIVMFY]-[LIVMFYC]-x-[LIVMFAH],[RKQN]-x(2)-{G}-x-[RH]-[GAS]-x-G-[KRQS]-x(8)-{L}-[HDN]-[LIVM]-{A}-[LIVMS]-x-[LIVM],[GSTA]-R-[NQ]-P-x(5)-{A}-x-{F}-x(2)-[LIVMFYW](2)-x(3)-[LIVMFYW]-x-[DE],[ASV]-S-C-[NT]-T-{S}-x-[LIM],"W-x(9,11)-[VFY]-[FYW]-x(6,7)-[GSTNE]-[GSTQCR]-[FYW]-{R}-{SA}-P″,H-[GN]-x(2)-[GC]-E−[DNT]-G-x-[LIVMAFT]-[QSAPH]-[GSA],[MFYGS]-x-[PST]-x(2)-K-[LIVMFYW]-{G}-W-[LIVMF]-{E}-[DENQTKR]-[ENQH],C-x(3)-[KRSN]-P-[KRAGL]-C-x(2)-C-x(5)-C,[FYW]-P-[GS]-N-[LIVM]-R-[EQ]-L-x-[NHAT],<M-R-[DE]-[IL],[TS]-[ST]-R-x(2)-[KR]-x(2)-[DE]-x(2)-[GA]-x(2)-Y-x-[FY]-[LIVMKHRT],"[LIVM](2)-x-D-D-x(2,4)-D-x(4)-R-R-[GH]",G-[GA]-G-[ASC]-[FY]-S-x-K-[DE],C-[STAGM]-G-[HFYL]-C-x-[ST],"G-[KRQEA]-x(3)-[FYVIM]-x-[ACVTI]-x(2)-[LIVMA]-[LIVMAT]-[AG]-[DN]-x(2,3)-G-x-[LIVMA]-[GS]-x-[SAG]-x(5,6)-[DEQGHS]-[LIVMARFY]-x(2,3)-[AS]-[LIVMFRY]",[FY]-[PA]-x-K-[SACV]-[NHCLFW]-x(4)-[LIVMF]-[LIVMTA]-x(2)-[LIVMA]-x(3)-[GTE],[LIVMF]-[DN]-x-F-P-[QHYWM]-[ST]-x-[HR]-[LIVMFYT]-E,[GSW]-x-[LIVTSACD]-[GH]-x(2)-[GSAE]-[GSHYQ]-x-[LIVTP]-[GAST]-[GAS]-x(3)-[LIVMT]-x-[HNS]-[GA]-x-[GTAC],H-x-C-G-G-N-V-G-D,"P–F-D-[LIVMFYQN]-[STAGPVMI]-E−[GACS]-E-x(0,2)-[EQLN]-[LIVMS]-x(1,2)-G″,[LIVMF]-x(2)-E−[AG]-[YWG]-[QRFGS]-[SG]-[STAN]-G-x-[SAF],G-x-[KRC]-[DENQRH]-L-[SA]-Y-x-I-[KRNSA],[LIVMFYSNAD]-x(2)-A-x(2)-R-[NH]-[KRQLYAT]-[LIVMFSA]-[KRA]-R-x-[LIVMTA]-[KR],[LIVM]-x(2)-H-[LIVMFY]-x(3)-{S}-x-D-x(2)-[STAGN]-x(3)-[LF]-x(2)-{A}-x(6)-[LIVM]-x(2)-[FY],[LIVFYAN]-[LIVMFA]-x(2)-D-[LIVMF]-[ND]-G-T-[LIV]-[LVY]-[STANLM],[IF]-x-[RH]-x(4)-[EQ]-R-x(2)-H-x(2)-[GAS]-[GASTFY]-[GAST],[GSTNP]-x(6)-[FYVHR]-[IVN]-[KEP]-x-G-[STIVKRQ]-Y-[DNQKRMV]-[EP]-x(3)-[LIMVA],G-x-[FYW]-x-[LIVMFYW]-x-[CST]-x-{PR}-{K}-x(2)-{S}-x-{LFH}-G-[LM]-x(3)-[LIVMFYW],[LIVMFA]-x-{GPRV}-[LIVMFYC](2)-{LPC}-[STAC]-[GSTANQEKR]-[STALV]-[HY]-[LIVMF]-G,"[STNAQ]-[LIAMV]-x(0,1)-[RNGSYKE]-x(4,5)-[LM]-[EIVLA]-x(2)-[GESD]-[LFYWHA]-[LIVC]-x(7)-[DNS]-[RKQG]-[RK]-x(6)-[TS]-x(2)-[GAS]","[GA]-[LIVM]-[PKV]-x(0,1)-E-x(3)-[NG]-E-x(1,3)-R-[VT]-[AG]-x-[ST]-P-x-[GSTVN]-[VA]-x(2)-[LI]-x-[KRHNGSED]-x-G″,G-[GNHD]-[SGA]-[GR]-x-R-x-[SGAWRV]-C-x(2)-[IV],A-L-[KR]-[IF]-[FY]-[STA]-[STAD]-[LIVMQ]-R,[LIVMFGAC]-[LIVMTADN]-[LIVFSA]-D-[ST]-G-[STAV]-[STAPDENQ]-{GQ}-[LIVMFSTNC]-{EGK}-[LIVMFGTA],[IV]-{K}-[TACI]-Y-[RKH]-{E}-[LM]-L-[DE],[LIVMF]-[LIMN]-E−[LIVMCA]-N-[PATLIVM]-[KR]-[LIVMSTAC],[ST]-x(3)-G-[DY]-G-[KR]-[IV]-[FW]-[LIVM]-x(2)-[LIVM],[RKH]-x-{Y}-{I}-x-{I}-{L}-D-x-M-G-x-N-x-[LIVMA],"[KFQ]-[RGMP]-[TN]-[FYWL]-[EQSG]-x(5)-[KRHS]-x(4,5)-G-F-x(2)-R″,"[DN]-P-[PAS]-R-x-G-x(14,19)-[LIVMAF]-[LIVMCAFT]-[YAHG]-x-[SAG]-C-[NAMDSYHKGQ]-x(1,2)-[TNKSI]",C-[DESN]-x-[CTS]-x(3)-I-x(3)-[RK]-x(4)-P-x(4)-[CSLAT]-x(2)-[CAYF],[FQ]-x-[LIVMFY]-x-[NH]-[PGT]-[NSKQR]-x(4)-C-x-C-[GSN]-x-S-F,[LIVMFYWCTA]-[LIVM]-[LIVMA]-[LIVMFC]-[DE]-D-[LIVMS]-[LIVM]-[STAVD]-[STAR]-[GAC]-x-[STAR],"[LIVMF]-x-[KRGTIEQSN]-x-[GSAIYN]-[KRQDAVLSIH]-[VGAIT]-[RSNAK]-x(0,1)-[KRAQ]-[SAKG]-[KYR]-[KLI]-[LYSFT]-[YF]-[LIM]-[RK]","[GA]-x(0,2)-[YSA]-x(0,1)-[VFY]-{SEDT}-C-x(1,2)-[PG]-x(0,1)-H-x(2,4)-[MQ]","[CHDS]-x(2)-[CND]-x(2)-[LIVM]-x-R-x(3)-[LIVMNR]-x-[LIVM]-x-[CN]-x(3,4)-[KRSN]-[HLFR]-x-[QCAV]-x-Q″,[GAVS]-[ST]-D-x-A-P-H-x(4)-K,[GN]-x-[DE]-[KRHST]-[LIVMFA]-[LIVMF]-P-[IV]-D-[LIVMFYWA]-[LIVMFYWK]-x-P-x-C-P-[PT],[GSA]-Q-x-K-S-[FY]-x-Q-x-K-[SA],"[RKN]-x-[LIVM]-x-G-[ST]-x(2)-[SNQ]-[LIVM]-G-x-{M}-[LIVM]-x(0,1)-[DENG]","<x(10,115)-[DENF]-[ST]-[LIVMF]-[LIVSTEQ]-V-{AGPN}-[AGP]-[STANEQPK]",D-[LIM]-H-[SANDT]-x-[QS]-[IMSTAVF]-[QMLPH]-[GA]-[FY]-F-x(2)-P-[LIVMFCT]-D,G-D-x-[LIV]-x-[LIVA]-x-[QEK]-x-[RK]-P-[LIV]-S,S-[LIVMFYW]-x-{KG}-x(3)-K-[LIVMFYWGH]-[LIVMFYWG]-x-{R}-x-[LIVMFYW]-{V}-[CA]-x(2)-[LIVMFYWQ]-{K}-x-[RK],G-x-T-L-x-H-E-H-[LIV],[AV]-x(3)-[GDNSR]-[LIVMSTAG]-x(3)-G-P-[LIVM]-x-[LIVM]-P-T,"E-G-[LIVMA]-[LIVM]-[LIVMA]-[KR]-x(5,8)-[YW]-[QNEKTI]-x(2,6)-[KRH]-x(3,5)-K-[LIVMFY]-K″,[LIVMFY]-x-{D}-[DENQGA]-x-{E}-x(2)-[LIVMFTA]-x-[KRV]-x(2)-[KW]-P-x(3)-[SEQ]-x(5)-{D}-{CG}-[LIVT]-[LIVGA]-[LIVFGAST],[LIVMRPA]-[LIVFY]-[PLNRKG]-[LIVMF]-E-x-[IV]-[LVCATI]-R-x(3)-[TAEYSI]-G-[ST],Y-x-D-x-N-H-K-P-E,[GAC]-[LIVM]-[ST]-E-x(2)-[GSAN]-G-[ST]-D-x(2)-[GSA],"[DENGQST]-[LIVMPF]-[LIVM]-x(1,2)-[KRNQELD]-[DENKGS]-[LIVM]-x(3)-[STG]-x-C-[EP]-H-H″,D-V-[LIV]-x(2)-G-H-[ST]-H-x(12)-[LIVMF]-N-P-G,"D-G-D-T-[LIVM]-x-[LIVMC]-x(9,10)-R-[LIVM]-x(2)-[LIVM]-D-x-P-E″,G-x-T-x-[KRM]-G-N-D-x(2)-R-F,"[DENQLF]-[KRVW]-N-[HRY]-[STAPV]-[SAC]-[LIVMFS]-[LIVMFSA]-[LIVMFS]-W-[GSV]-x(2,3)-N-E″,"[GRH]-[DEQKG]-[STVM]-[LIVMA](3)-[GA]-G-[LIVMFY]-x(11)-[LIVM]-P-[LIVMFYWGS]-[LIVMF]-[GSAE]-x-[LIVMS]-P-[LIVMFYW]-[LIVMFYWS]-x(2,3)-[LV]-[FK]",[GS]-[LIVMFYTAC]-[GSTA]-K-x(2)-[GSALVN]-[LIVMFA]-x-[GNAR]-{V}-R-[LIVMA]-[GA],R-[LIVMFSTAN]-F-[GASTNP]-Y-x-D-[AST]-[QEH],N-x(2)-H-[GA]-S-D-[GSA]-[LIVMPKNE],[DA]-[AI]-[SGA]-[NQS]-[LIVMF](2)-K-[PT]-x-[LM]-x(2)-G,"[IMGV]-x(2)-[LIVA]-x(2,3)-[LIVMY]-[GAS]-x(2)-[LMSF]-[GSNH]-[PTKR]-[KRAVG]-[GN]-x-[LIMF]-P-[DENSTKQPRAGVI]",[NHS]-x(2)-[NK]-x-[TINAS]-[DN]-G-[ILVM]-D-G-[LM],H-[FW]-x-[LIVM]-x-G-x(5)-[LV]-H-x(3)-[DE],C-D-G-P-[GE]-R-G-G-T-C,[DENQ]-x(6)-[LIVMF]-[GA]-x(2)-[LIVM]-A-[LIVM]-P-H-[GAC],K-[LIVMF]-D-G-[LIVMAS]-[SAG]-x(4)-Y-x(2)-[GRD]-x-[LF]-x(4)-[ST]-R-G-[DN]-G-x(2)-G-[DE]-[DENL],[SGALC]-[LIMF]-[LIVMF]-T-D-[GA]-R-[LIVMFY]-S-[GA]-[GAV]-[ST],E-R-E-x(2)-[DE]-[LIVMFY](2)-x(6)-[HK]-x(3)-[KRP]-x-[LIVM]-[LIVMYS],[GS]-G-G-x(2)-[GSA]-[QK]-x(2)-[SA]-x(3)-[GSA]-x-[GSTAV]-[KR]-[GSALVD]-[LIFV],"[AG]-G-x(0,1)-[GAP]-x-N-{AGLS}-[STA]-x(2)-{A}-x-{G}-{GNKA}-[GS]-x(9)-G″,[DEQ]-[KRQT]-[LMF]-E−[FYW]-[LV]-G-D-[SARHG],[GN]-[LIVMS]-K-G-[GST]-[AG]-[AST]-G-[GAS]-G-[YLHRKF],"[LI]-x-[STN]-[HN]-x-H-[GSTAD]-D-x(2)-G-[GP]-x(7,8)-[GS]",[GE]-[SAV]-x-[LIVM](2)-D-[LIVMF]-G-[GPA]-x(2)-[STA]-x-P,"C-x-C-x(2)-[GP]-[FYW]-x(4,8)-C″,[TG]-[STV]-x(8)-[LIVMF]-x(2)-R-x(3)-[DEQNH]-x(2)-{S}-x(4)-[IFY]-x(7)-[LIVMF]-x(3)-[LIVMF]-x(5)-{I}-x(5)-[LIVMFA]-x(2)-[LIVMF],K-x-[WQA]-[CA]-x(2)-[FYH](2)-x-[LIVM]-x-[HY]-R-x-E-x-R-G-[LIVMT]-G-G-[LIVM]-F-[FY]-D,"[LVAGC]-[LIF]-G-x(4)-[LIVMF]-P-W-x(4,5)-[DE]-x(3)-[FYIV]-x(3)-[STIQ]","[LIVM]-[STAG]-[RHNWM]-x(2)-[LIM]-[GA]-x-[LIVMFYAS]-[LIVSC]-[GA]-x-[STACN]-x(2)-[MST]-x(1,2)-[GSTN]-R-x-[LIVMF]-x(2)-[LIVMF]",[LIVMY]-x-[LIVMF]-x-G-G-x-[ST]-{LS}-[LIVM]-P-x-[LIVM]-x-[DEQKRSTA],[LIVM](2)-[FYW]-x(10)-C-x(2)-C-G-x(2)-[FY]-K-L,H-A-Y-[LIVM]-x-G-x(2)-[LIVM]-E-x-M-A-x-S-D-N-x-[LIVM]-R-A-G-x-T-P-K,"[LIVM]-x(2)-G-[LIVMFCT]-G-x-[GA]-[LIVMFA]-x(3)-{V}-x(4)-G-x(3,5)-[GATP]-{G}-x-G-[RKH]",[EDQH]-{K}-K-{VEDI}-[DN]-G-{GLYN}-R-[GACIVM],[FYPH]-x(4)-[LIVM]-G-N-H-E-F-[DN],[IV]-T-x-E-x(2)-[DE]-x(3)-G-A-x-[SAKR],R-[SHF]-D-[PSV]-[CSAVT]-x(4)-[SGAIVM]-x-[IVGSTAPM]-[LIVM]-x-E−[STAHNCG]-[LIVMA],[FM]-x-[DV]-D-x(2)-[GS]-T-[GSA]-x-[IV]-x-[LIVMAT]-[GAST]-[GASTC]-[LIVMFA]-[LIVMFY],[LIVMH]-H-[RT]-[GA]-x-E-K-[LIVMTN]-x-E-x-[KRQ],[LIVM]-x-{L}-T-G-G-T-[IV]-[AGS],H-[GSAD]-x-Y-[LIF]-[LIMN]-N-[LIVMFCAP]-[AGC],[LIVMF]-T-S-P-P-[FY],[LT]-L-E−[FY]-[AVC]-[DE]-[DE]-[KNQHT]-[LMT],[RGT]-[LIVMFY]-[DN]-x-[ST]-E−[LIVMFY]-x-[ED]-[KRQEAS]-x-[STA]-x-[STAD]-[KRS]-[LIVM]-x-G-[STAP],[LIV]-x-G-x-V-Q-[GH]-V-x-[FM]-R,"F-[LF]-x(4)-[GE]-G-[PAT]-x(2)-[YW]-x-[GSE]-[KRQAE]-x(1,5)-[LIVM]-x(3)-H″,[DEQHY]-[LIVMFYA]-x-[GSTMVA]-[GSTAV]-[ST]-[STVM]-[HQ]-K-[STG]-[LFMI]-x-[GAS]-[PGAC]-[RQ]-[GSARH]-[GA],[STANQ]-[ET]-C-x(5)-G-D-[DN]-[LIVMT]-x-[STAGR]-[LIVMFYST],[HA]-[GSYR]-[LIVMT]-[SG]-H-x-[LIV]-G-[LIVMNKS]-x-[IVEL]-[HNC]-[DEV],"R-x(2)-[LIVMT]-x(2,3)-[FWY]-[QNYDI]-x(8,13)-[LVESI]-x-P-C-[HAVMLC]-x(3)-[QMTLHD]-[FYWL]-x(0,1)-[LV]",[DEN]-[WV]-x(3)-G-[RKNM]-x(6)-[FYW]-[SV]-x(4)-[LIVM]-N-x(2)-N-V-x(2)-L-[RKT],Y-x-[NQHD]-[KHR]-[DE]-[IVA]-F-[LM]-R-[ED],D-x-[WF]-E-H-[STA]-[FY](2),C-x(2)-[STAQ]-x-[STAMV]-C-[STA]-T-C-[HR],"[LIVM]-[KRVLYFS]-[GKR]-M-[LIV]-[PST]-x(4,5)-[GSKR]-[NQEKRAH]-x(5)-[LIVM]-x-[AIVL]-[LFYV]-x-[GDNS]",[LIVM]-R-x(2)-P-D-x-[LIVM](3)-G-E−[LIVM]-R-D,[GLES]-x-[LIVM]-x(2)-L-[KR]-[KRHNS]-x-K-x(5)-[LIVM]-x(2)-[GNKADS]-x-[DEN]-[CRG]-[GI],[LIVM](2)-[GSA]-x-G-G-[IV]-x-[STGDN]-x(3)-[ACV]-x(2)-{A}-{R}-x-{L}-G-A,[ELAS]-[LIVMF]-[NVCKGST]-[SCVA]-[QE]-T-D-[FS]-[VLA]-[SAT]-[KRNLAQS],[KRC]-[GSAT]-x(4)-[FYWLMH]-[DQNGKRH]-x-P-x-[LIVMFY]-x(3)-H-x(2)-[GSA]-H-[LIVMFA],H–N–H–P-[SQ]-G,[LIVMFSTC]-[LIVFYS]-[LIV]-[LIVMST]-E-N-G-[LIVMFAR]-[CSAGN],H-x-S-G-H-[GA]-x(3)-[DE]-x(3)-[LM]-x(5)-P-x(3)-[LIVM]-P-x-H-G-[DE],Q-[DEK]-x-x-[LIVMGTA]-[GA]-D-G-T,N-x-[LIVMFYWD]-R-[STACN](2)-H-Y-P-x(4)-[LIVMFYWS](2)-x(3)-[DN]-x(2)-G-[LIVMFYW](4),"[LIVMFY]-x-P-[ILT]-x-[DEN]-[KR]-[LIVMFA](3)-[KREQS]-x(8,9)-[SG]-x-[LIVMFY](3)","[DESH]-x(4,5)-[STVG]-{EVKD}-[AS]-[FYI]-K-[DLIFSA]-[RLVMF]-[GA]-[LIVMGA]","[STAN]-x-[CH]-x(2,3)-C-[STAG]-[GSTVMF]-x-C-x-[LIVMFYW]-x-[LIVMA]-x(3,4)-[DENQKHT]",P-x(2)-C-[YWSD]-x(7)-[GA]-x-C-R-x-C,G-F-R-G-E−[AG]-L,[LIVMFANT]-[LIVM]-x-[LIVMA]-N-x-G-S-[ST](2)-x-[KE],"G-x-[IVT]-x(2)-[LIVMF]-x-[NAK]-[GS]-[GA]-G-[LMAI]-[STAV]-x(4)-[DN]-x-[LIVM]-x(3,4)-[GD]-[GREAK]","[GSA]-x-[LIVMFYW]-{D}-G-[LIVM]-x(7,8)-[HDENQ]-[LIVMF]-{PEQ}-{DTAI}-[AS]-[STALIVM]-[LIVMFY]-[DEQ]",[SAPG]-[LIVMST]-[CS]-[STACG]-P-[STA]-R-x(2)-[LIVMFW](2)-[TAR]-G,[LIVM]-x-[LIVM](2)-[HEA]-[TI]-x-D-x-H-[GSA]-x-[LIVMF],[LIVMC]-[LIVM]-Y-[KR]-x(4)-L-Y-F,"[DES]-[IVT]-x(4)-H-[PT]-[FAVY]-[FYW]-[TISN]-x(9,13)-[GN]-[KRHNQ]",[GSAH]-x-[LIVMF](3)-D-E−[ALIV]-H-[NECR],"[LIVMFYWCS]-[LIVMFYWCAH]-x-D-[ED]-[IVA]-x(2,3)-[GAT]-[LIVMFAGCYN]-x(0,1)-[RSACLIH]-x-[GSADEHRM]-x(10,16)-[DH]-[LIVMFCAG]-[LIVMFYSTAR]-x(2)-[GSA]-K-x(2,3)-[GSTADNV]-[GSAC]",K-x(3)-[KRCV]-x-[LIVM]-W-[IVN]-[STNALVQCMI]-[RH]-[LIVM]-[NS]-x(3)-[RKHSG],[DNSTAGC]-[GSTAPIMVQH]-x(2)-G-[DE]-S-G-[GS]-[SAPHV]-[LIVMFYWH]-[LIVMFYSTANQH],K-[KR]-C-G-H-[LMQR],[RK]-x-P-N-S-[AR]-x-R,[LIVMFYH]-[LIVMFST]-H-[AG]-[AGSP]-[LIVMNQA]-[AG]-C,"[LIVFYCHT]-[DGH]-[LIVMFYAC]-[LIVMFYA]-x(2)-[GSTAC]-[GSTA]-[HQR]-K-x(4,6)-G-x-[GSAT]-x-[LIVMFYSAC]",[IV]-D-L-G-T-[ST]-x-[SC],G-[LIVM]-H-[STAV]-R-[PAS]-[GSTA]-[STAMVN],G-x(2)-[LIVMFA]-[LIVMF](2)-H-[LIVMF]-G-[LIVMF]-x-T-[LIVA],G-x-[SA]-G-E−[LIVM]-R-Y-P-S-Y,"[GS]-[LIVMFYSP]-x(2,3)-[TS]-[LIVMTA]-x(2)-[LIVM]-x(5)-[LIVQSA]-[STAGENQH]-x-[GPART]-x-[LIVMFA]-[FYSTNRH]-x-[HFYRA]-[FVW]-x-[DNSTKAG]-[KQMT]-x(2,3)-[LIVM]",[GSARY]-[LIVMF]-[CT]-[LIVMFY]-D-T-C-H,[LIVM]-x(2)-[LIVMA]-x(2)-[LIVM]-x-R-H-[GN]-x-R-x-[PAS],G-T-S-x-[SA]-x-P-x-{L}-[STAVC]-[AG],[LIVMFATQ]-[LIVMA]-x(2)-H-x-G-x-[GT]-x-[ST]-[LIVMA]-x-[TAVC]-x(3)-G,[AT]-x-[SAGCN]-[SAGC]-[LIVM]-[DEQ]-x-A-[LA]-x-[DE]-[LIA]-x-[GA]-[KRQ]-x(4)-[PSA]-[LIV]-x(2)-L-[LIVMF]-G,[GDN]-x(2)-[LIVF]-x(3)-{VH}-{M}-[LIVMFCA]-x(2)-[LIVMFA]-{LDFY}-{KPE}-x-K-[GSTAIVW]-[STAIVQDN]-x(2)-[LIVMFS]-x(5)-[GCN]-x-[LIVMFY],[HNQA]-{D}-N-P-[STA]-[LIVMF]-[ST]-[LIVMF]-[GSTAFY],[LIVMF]-P-C-H-R-[LIVMF](2),[DNHKR]-[LIVMF]-x-[LIVMF](2)-[VSTAC]-[STAC]-G-x-G-[GKN]-G-T-G-[ST]-G-[GSARC]-[STA]-P-[LIVMFT]-[LIVMF]-[SGAV],"G-x(7)-[DEN]-G-x(6)-[FY]-x-A-[DNG]-x(2,3)-G-[FY]-x-[APV]","[STALIV]-[LIVF]-x-[DE]-x(6,7)-P-x(4)-[ALIV]-x-[GST]-x(2)-D-[TAIVM]-[LIVMF]-x(4)-E″,"[LIVMF]-G-E-x-[GAS]-[LIVM]-x(5,11)-R-[STAQ]-A-x-[LIVMA]-x-[STACV]","[LIVM]-[PK]-x-[GSTA]-x(0,1)-G-[LM]-[GS]-S-S-[GSA]-[GSTAC]","N-[ST]-D-x-[QS]-x-L-x(16,18)-G-x-G-[ATVS]-G-[GSAN]-x-P-x(2)-G″,S-x(2)-S-[PK]-[LIVMF]-[AG]-x-[SAGNE]-[LIVM]-[LIVY]-x(4)-[DNG]-[DE],[LIVM]-x-[LIVMFYT]-x(3)-[LIVMT]-[DENQK]-x-{G}-[LIVM]-x-[GSA]-G-[LIVMFYGA]-{S}-[LIVM]-[KRHENQ]-x-[GSEN],[LIVMF]-x-G-[LIVMFA]-{V}-x-G-{KP}-x(7)-[LIFY]-x(2)-[EQ]-x(6)-[RK],[DT]-[KRP]-[YQ]-[GQ]-R-x-[LVY]-[GA]-x-[IV]-[FYW],C-{C}-{C}-[GA]-{C}-C-[GAST]-{CPDEKRHFYW}-C,[LIVMT]-x-[LIVM]-[KR]-L-[STAK]-R-{E}-G-[AKR],"[FYLV]-[DNST]-[PHEAYVS]-x(2)-[HMACNQ]-x-[ALV]-[LIVMTNSF]-x(16,21)-[GYP]-[FY]-x(3,4)-[DENGKS]-x(2,3)-[LIV]-[KRIV]-x-[STAG]-x-V-x(0,1)-[IV]",[LIVMGSTAN]-{IEVK}-H-[GSACE]-[LIVM]-{GPSI}-[LIVMAT](2)-G-{SLAG}-[GSADNH],[LIVMFY]-[DN]-G-[LIVMF]-[DN]-[LIVMF]-[DN]-x-E,"R-x(3)-[LIVMTA]-[DENQSTHKF]-x(5,6)-[GSN]-G-H-[PLIVMF]-[GSTA]-x(2)-[LIMC]-[GS]","H-x(2,4)-[SC]-x(2)-{A}-x-[LIVMF](2)-[ST]-H-G″,[WYV]-D-x-[AC]-[GSA]-[GSAPV]-x-[LIVFACP]-[LIVM]-[LIVAC]-x(3)-[GH]-[GA],[FL]-x(6)-[DN]-x(2)-[AGS]-x-[ST]-x-G-[KRH]-G-x(2)-G-x(3)-R,[GTA]-{QNAG}-{GSV}-[LIVN]-x-[IVMF]-[ST]-E−[LIY]-[DN]-[LIVMF],[GAP]-[LIVMFA]-[STAVDN]-x-{H}-x(2)-[GSAV]-[LIVMFY](2)-Y-[ND]-x(3)-[LIVMF]-x-[KNDE],[GSA]-[LIVMF]-x-[LIVM]-[ST]-[PGA]-S-H-[NIC]-P,"[LIVMAC]-[LIVMFYWT]-[DE]-x-G-[STAPVLCG]-G-x-[GAS]-x-[LIVMF]-[ST]-x(2,3)-[LIVMA]-x(5,8)-[LIVMYF]-x-[STAGVLC]-[LIVMFYHCS]-E-x-D″,[STA]-[LIVMF]-x-[LIVM]-x-D-E−[LIVMFY]-[GCA]-[RKHAS]-[GS]-[GST]-x(4)-G,"[STAIV]-{ERDL}-[LIVMF]-[LIVM]-D-[DSTA]-G-[LIVMFC]-x(2,3)-[DNH]",[VTI]-x-T-A-H-P-T-[EQ]-x(2)-R-[KRHAQ],"[GVPS]-x-[GKS]-x-[KRS]-x(3)-[FL]-x(2)-G-x(0,1)-C-x(3)-C-x(2)-C-x-[NLF]",[SN]-P-x-[LV]-x(2)-H-A-x(3)-F,[LIVMFE]-[FY]-P-W-M-[KRQTA],"[LIVMF]-H-[LIVMFY]-D-[LIVM]-x-D-x(1,2)-[FY]-[LIVM]-x-N-x-[STAV]",[LM]-[LF]-T-x-R-[SA]-x(3)-[RK]-x(3)-G-x(3)-F-P-G-G,[LIVM]-[VIC]-x-{H}-G-[DENQTA]-x-[GAC]-{L}-x-[LIVMFY](4)-x(2)-G,"W-x(0,2)-[KDN]-{Q}-{L}-K-[KRE]-[LI]-E−[RKN]","[AT]-x(1,2)-[RK](2)-[GP]-R-G-R-P-[RK]-x",[GSTALIVMFYWC]-[GSTANCPDE]-{EDPKRH}-x-{PQ}-[LIVMNQGA]-{RK}-{RK}-[LIVMFT]-[GSTANC]-[LIVMFYWSTAC]-[DENH]-R-[FYWCSH]-{PE}-x-[LIVM],E-Y-F-G-[SA](2)-L-W-x-L-Y-K,"[DEH]-[LIVMF]-[LIVMFC]-[LIVMF]-R-[STPV]-[SGAC]-[GEN]-x(1,2)-R-x-S-x-[FY]-[LMFV]-[LIPMVT]-[YWL]",[KRHEQSTAG]-G-[FYLIVM]-[ST]-[LT]-[LIVP]-E−[LIVMFWSTAG](14),N-[LIVMFY]-x(5)-C-x-T-R-[LIVMF]-x-[LIVMF]-x-[LIVM]-x-[DQEN],"[KR]-x(2)-E-x(3)-[LIVMF]-x(8,12)-[LIVMF](2)-[SA]-x-G(3)-x-[LIVMFG]",G-x(2)-[LIVMFY](2)-x-[IF]-x-E-x(2)-[LIVM]-x-G-Y-P,[LIVM]-x-[DE]-[LIVM]-A-x(2)-[STAGV]-x-V-[GSTP]-x(2)-[STAG]-[LIVMA]-x(2)-[LIVMFYAN]-[LIVMC],[LIVM]-[DERA]-x-R-[LI]-x(3)-[LIVMC]-[VMFYHQL]-[KRTS]-x(3)-[STAGCVF]-x-[ST]-x(3)-[SAI]-[KRQ]-x-[LIVMF](2),"[LIV]-G-{P}-G-{P}-[FYWMGSTNH]-[SGA]-{PW}-[LIVCAT]-{PD}-x-[GSTACLIVMFY]-x(5,18)-[LIVMFYWCSTAR]-[AIVP]-[LIVMFAGCKR]-K″,[LIVMFA]-[STAGC](2)-G-x-{TAV}-H-[STAGLI]-[LIVMFA]-{KI}-[LIVM],D-[LIVMFYWSAP]-H-[LIVA]-H-[LIVF]-[RN]-x-[PGANF],[FW]-[SGNH]-x-[GD]-{F}-[RKHPT]-{P}-C-[LIVMFAP]-[GAD],"S-x-[GS]-x(2)-D-x(5)-[LIVW]-x(10,12)-[LIV]-x(2)-[KR]-P-G-[KRL]-P-x(2)-[LIVMF]-[GA]","[LIVFAG]-x-[GASV]-[LIVFA]-x-[IV]-H-x(3)-[LIVM]-[GSTAE]-[STANH]-x(1,3)-[STN]-W-[LIVMFYW]",C-D-K-x(2)-P-[GA]-x(3)-[GA],[LIV]-[STAG]-V-[DEQV]-[FLI]-D-[ST],[HQ]-[EQ]-x(3)-H-x-[LMA]-[NEQHRCS]-[GSTA]-H-[LIVMSTAC](2)-x-E,N-x-G-x-R-[LIVM]-D-[LIVMFYH]-x-[LV]-x-S,"[CH]-[AGV]-E-x(2)-[LIVMFGAT]-[LIVM]-x(17,33)-P-C-x(2,8)-C-x(3)-[LIVM]",[GSTNAD]-x(2)-[GAS]-x-G-[GC]-[IM]-x-[STAG]-K-[LIVMCT]-x-[SAI]-[TCAGFS]-x(2)-[GALVCMI],K-x(2)-[LIVF]-x(4)-[LIVF]-D-x(3)-R-x(2)-L-x(5)-[LIV]-Y,"[IVTPM]-[DEG]-x(2,3)-[AYEPQ]-G-[PT]-[ST]-[ED]-[LIVSTA]-[LIVMAECGFT]-[LIVMA]-[LIVMAYF]-[ACNDSTI]-x(2,3)-[ACNGVST]-x(4,6)-[LIVMAC]-[AVLKIT]-[SACLYWNRMTV]-[DEG]-[LIVMFCA]-[LIVMKFR]-[SAGVI]-x(2)-E-H″,[STIV]-x-R-[IVT]-[CSA]-G-Y-{GI}-[GACV],"[LIVMA]-x-[GT]-x-[TA]-[DAN]-x(2,3)-[DG]-[GSTPNKQ]-x(2)-[LFYDEPAVI]-[NQS]-x(2)-[LI]-[SG]-[QEA]-[KRQENAD]-R-A-x(2)-[LVAIT]-x(3)-[LIVMF]-x(4,5)-[LIVMF]-x(4)-[LIVM]-x(3)-[SGW]-x-G″,"G-x-[GA]-x-[AG]-x-K-x-[EQA]-[IVM]-x(16,19)-D-x-[SAVT]-D-[AG]-x-[AGS]-[LIVMCA]-[ACS]",Q-[LIVM]-x-N-x-A-x-[LIVM]-P-x-I-x(6)-[LIVM]-P-D-x-H-x-G-x-G-x(2)-[IV]-G,[SA]-[LIVM]-[NGS]-[STA]-D-D-P,G-x-[KN]-[LIVMFA]-[STAC]-[GSTNR]-x-[HSTA]-[GSAI]-[QNH]-K-[GL]-[IVTEC],[IVTAS]-[LIVM]-x(2)-[LF]-x-[LI]-x-[KRHQEG]-x(2)-[STNQH]-x-[IVTR]-x(10)-[LMSN]-[LIV]-x(2)-[LIVA]-x(2)-[LMFY]-[IVT],"[PA]-[ASTPV]-R-[SACVF]-x-[LIVMFY]-x(2)-[GSAKR]-x-[LMVA]-x(5,8)-[LIVM]-E−[MI]",[LIVMF]-[GSA]-x(5)-P-x(4)-[LIVMFYW]-x-[LIVMF]-x-G-D-[GSA]-[GSAC],F-E-N-[RK]-G-x(3)-G-x(4)-H-P-H-x-Q,[LMFYCVI]-[DN]-R-x(3)-[PGA]-L-[LIVMCA]-E−[LIVMT]-x-[STL]-x-[PA],G-H-E-x-{EL}-G-{AP}-x(4)-[GA]-x(2)-[IVSAC],[IVTL]-x(3)-[KR]-x(3)-[KRQ]-[KT]-x(6)-G-[HFY]-[RK]-[RQT]-x(2)-[STL],G-x-D-x-[LIVM](2)-[IV]-K-P-[GSA]-x(2)-Y,G-[GA]-x-[STN]-x-H-[STA]-[STAV]-[LIVM](2)-[STAV]-[RG],"[GD]-[VI]-[LIVM]-x(0,1)-[GS]-x(5)-[FY]-x-[LIVM]-[FYWL]-[GS]-[DNTHKWE]-[DNTAS]-[IV]-[DNTAY]-x(5)-[DEC]","G-[NTKQ]-x(0,5)-[GA]-[LVFY]-[GH]-H-[IVF]-[CGA]-x-[STAGLE]-x(2)-[DNC]","[KR]-x(1,3)-[RKSAQ]-N-{VL}-x-[SAQ](2)-{L}-[RKTAENQ]-x-R-{S}-[RK]",[LIVMFY]-[DH]-x-[LIVM]-[GA]-E-R-x(3)-[LIF]-[GDN]-x(2)-[PA],Y-G-G-[LIV]-T-{I}-{N}-x(2)-N,P-[LIVM]-x-[LIVM]-H-x-R-x-[TA]-x-[DE],[RK](2)-[AM]-[IVFYT]-[IV]-[RKT]-L-[STANEQK]-x(7)-[LIVMFT],[STA]-x-[STAC](2)-x(2)-[STA]-D-[LIVMY](2)-L-P-x-[STAC](2)-x(2)-E,W-[FY]-x-G-[ST]-[AS]-[DNSH]-[AS]-[LIVMFYW],P-[SAP]-[LIV]-[DNH]-{LKGN}-{F}-{S}-S-{DCPH}-S,[LIVM]-G-x-[LIVM]-G-G-[AG]-T,"[LIVMF]-[LIVMFC]-x-[ST]-x-H-[GS]-[LIVM]-P-x(4,5)-[DENQKRLHAFSTI]-x-[GN]-[DPC]-x(1,4)-[YA]",[KRHQSA]-[DENQ]-E-L>,[LIVSPADNK]-x(9)-{P}-x(2)-Y-[PSTAGNCV]-[STAGNQCIVM]-[STAGC]-K-{PC}-[SAGFYR]-[LIVMSTAGD]-x-{K}-[LIVMFYW]-{D}-x-{YR}-[LIVMFYWGAPTHQ]-[GSACQRHM],"[DN]-R-x-R-[LIVM]-[LIVMN]-x-[STA]-[STAQ]-F-[LIVMFA]-x-K-x-L-x(2,3)-W-[KRQ]","[LIVMTR]-x-[LIVMT]-[LIVMF]-x-[GATMC]-[ST]-[NS]-x(4)-[LIVM]-D-x-[AS]-[LIFAV]-x(1,2)-R″,P-x(2)-[LIVMF](2)-[LIVMS]-x-[GDN]-x(3)-[DENL]-x(3)-[LIVM]-x-E-x(4)-[GNQKRH]-[LIVM]-[AP],T-x-[GS]-x(2)-H-[LIVMF]-x(3)-E−[DE]-x-P,[LIVMAC]-[LIVFYWA]-{DYP}-[DN]-P-P-[FYW],[SA]-[GS]-R-[GA]-[LIV]-x(2)-[TAP]-[GAS]-G-T-x-D-x-[LIVMF]-[EDS],[PS]-[DENS]-x-Y-K-[GA]-K-G-[LIVM],[GSA]-x(4)-[GK]-[GSTA]-[LIVFSTA]-[GST]-x(3)-[NQRK]-x-G-[NHY]-x(2)-P-[RTV],[STDNQ]-G-[KRNQMHSI]-x(6)-[LIVM]-x(4)-[LIVMC]-[GSD]-x(2)-[LFI]-[GAS]-[DE]-[FYM]-x(2)-[ST],"[NSK]-[LIMYTV]-[FYDNH]-[GEA]-[DNGSTY]-[IMVYL]-x-[STGDN]-[DN]-x(1,2)-[SGAP]-x(3,4)-[GE]-[STG]-[LIVMPA]-[GA]-[LIVMF]","A-x(3)-G-[LIVMFY]-[STAG]-x(2,3)-[DNS]-P-x(2)-D-[LIVM]-x-G-x-D-x(3)-K″,[LIVMF](2)-D-E-A-D-[RKEN]-x-[LIVMFYGSTN],[LIVAC]-x-[LIVM](2)-[SAPCV]-K-[LIV]-E−[NKRST]-x-[DEQHS]-[GSTA]-[LIVM],"[IVMSEQ]-E-x(1,2)-[LIVTA]-[HY]-[GSA]-x-[STAVM]-Y-x(2)-[LIVMQ]-x(3)-[LIVFY]-[IVFYCSA]",[LIVMFY]-{E}-{VES}-[STG]-[STAG]-G-[ST]-[STEI]-[SG]-x-[PASLIVM]-[KR],[KRHD]-x-[GA]-[PSAE]-R-x(2)-D-[LIV]-D-[LIVM](2),[LIVMFW]-H-x-N-[DEG]-[SA]-x(4)-[GNAQ]-x(3)-D-x-H,[FYV]-[PS]-[LIVMC]-[LIVMA]-[LIVM]-[KR]-[PSA]-[STA]-x(3)-[SG]-G-x-[AG],C-[SA]-D-S-R-[LIVM]-x-[AP],D-[LIVMFY]-[DNV]-x-[DNS]-x(2)-[LIVM]-[DN]-[SALM]-x-D-x(3)-[LIVMF]-x-[RKS]-x-[LIVMF],[GP]-[DEQGSANPHVT]-[DN]-G-[PAEQ]-[ST]-[HQ]-x-[PAGM]-[LIVMYACNQS]-[DEFYWLA]-x(2)-[STAPG]-x(2)-[RGANQS],[NS]-[TS]-D-A-E-G-R-[LVMI],[KNQ]-x(2)-{K}-x(3)-{A}-{L}-x(9)-[LIVMFY]-x(2)-[DENHR]-x(2)-[GS]-[LIVMF]-[STDNQC]-[VTA]-x-[DENQKHPSA]-[LIVMSAD]-x(2)-[LIMF]-[KR],[APV]-[GS]-M-G-[LIVMN]-Y-[IVC]-[LIVMFY]-x(2)-[DENPHKRQS],"[EQ]-x-L-Y-[DEQSTLM]-x(3,12)-[LIVST]-[ST]-Y-x-R-[ST]-[DEQSN]",G-[FYIL]-[DE]-[LIVMT]-[DE]-[LIVMF]-{PS}-{YG}-x-[LIVMA]-[VAGC]-{TPRG}-{GL}-[LIVMAGN],[LIVMFY]-G-x(2)-[FYL]-Q-[LIVM]-x-D-D-[LIVMFY]-x-[DNG],"[GA]-[LIV](3)-x(9,10)-[DNS]-G-x(4)-[FY]-x(2)-[NT]-x(2)-V-[LIV]",[GE]-x(2)-[LIV](2)-[STY]-[ST]-{A}-x-G-[LIVM](2)-x(4)-[AG]-[KRHAYIL],[EQ]-x(4)-[HGQ]-x(5)-[GSTA]-x(3)-[FYV]-x(3)-[AG]-x(2)-[AV]-H-x(7)-P,[LIVMY]-[DE]-x-H-H-x(2)-E-x(2)-[GCA]-[LIVM]-[STAVCL]-[LIVMF],[STA]-x(5)-G-x-[QKRN]-x(2)-[LIVMQ]-[KRQT]-x(2)-[KR]-x-[GS]-x(2)-[KQ]-x-[LIVM](3),[LIVMFY](2)-[EK]-x-G-[LIVM]-[GA]-G-x(2)-D-x-[GST]-x-[LIVM](2),"[GSA]-x-[LIVMFA]-[ASM]-x(2)-[STACLIV]-[GSDENQR]-[LIVC]-[STANHK]-x(3)-[LIVM]-[RHF]-x-[YW]-[DEQ]-x(2,3)-[GHDNQ]-[LIVMF](2)",R-M-G-x-[GR]-K-G-x(4)-[FWKR],"[GSK]-F-x(2)-[LIVMF]-x(4)-[RKEQA]-x(2)-[RST]-x(1,2)-[GA]-x-[KN]-P-x-[TN]","R-{G}-x(2)-[LIVM]-x(3)-[LIVM]-x(16,17)-[STA]-x(2)-T-[LIVMA]-[RH]-[KRNAQ]-D-[LIVMF]",[DE]-x-A-[LIY]-[KR]-[RA]-[FL]-K-[KR]-x(3)-[KR],L-R-[DE]-G-x-Q-x(4)-{L}-x(5)-K,[LIV]-{KG}-[LIVFY]-[LIVMST]-G-[HYWV]-S-{YAG}-G-[GSTAC],S–V-A-G-L-G-G-C-P-Y,"H–F-x(2)-[EQ]-[ENQ]-x(2)-[LMF]-x(4,7)-[FY]-x(5,6)-H-x(3)-[HR]","G-x(8,9)-G-x-[STA]-H-[LIVMFY]-[LIVMC]-[DERN]-[HRKL]-[LMFAT]-x-[LFSTH]-x-[GSTAN]-[GST]",[LIV]-[GAED]-x(2)-[STAV]-x-[LIV]-x(3)-[LIVAC]-x-[LIV]-[GAED]-x(2)-[STAVR]-x-[LIV]-[GAED]-x(2)-[STAV]-x-[LIV]-x(3)-[LIV],[RK]-x(4)-[GAS]-H-x-[QL]-[QR]-[GS]-[GF]-x(5)-[DE]-[RL],[GA]-[IMFAT]-H-[LIVF]-H-{S}-x-[GP]-[SDG]-x-[STAGDE],"A-x(3)-[GDTN]-[IF]-x-[DNQTKEH]-x-[DEAQ]-x-[LIVM]-x-[LIVMC]-x-[NS]-x(2)-[GS]-x(4,5)-[AV]-x-[LIVMEF]-[STY]",K–F-G-x-G-D-G,[APF]-D-[LIVMF](2)-{T}-[LIVM]-Q-E−{G}-K,[LIVM]-[LIVMF]-G-[GAV]-G-x-[AV]-[GA]-x(2)-[SA]-x(3)-[GA]-x-[SGR]-[LIVM]-[GN]-A-x-V-x(3)-[DE],"[GS]-[STG]-[LIVM]-[STG]-[SAC]-S-G-[DH]-L-x-[PN]-L-[SA]-x(2,3)-[SAGVTL]",C–K-x(2)-N-T-F,R-P-[LIVMT]-x(3)-[LIVM]-x(6)-[LIVMWPK]-x(4)-S-x(2)-H-R-x-[ST],[LIVM]-x-[AG]-[LIVMF](2)-N-x-T-x-[DN]-S-[FLMI]-x-D-x-[SG],"[FYVMT]-x(1,3)-[LIVMH]-[APNT]-[LIVM]-x(1,2)-[LIVM]-H-x-D-H-[GACH]",[SAG]-G-G-T-G-[SA]-G,"[LIVM]-[DNG]-[LIVMF]-N-x-G-C-[PS]-x(3,4)-[LIVMASQ]-x(5,6)-G-[SACY]",G-A-K-R-H,T-x(2)-[LIVMF]-G-x-A-[SAC]-S-[MSA]-[PAG]-[STA],[LIV]-[LIFYMV]-x-[LIVM]-D-[DEA]-[LIVF]-x(2)-[EHCGK]-L-D-x(2)-[KRH]-x(3)-[LIVF],G-S-x(2)-M-x-{RS}-K-x-N,[IV]-G-[KR]-[ST]-G-x-[LIVM]-[STNK]-x-[VTLYF]-x(2)-[LVMF]-x-[PS]-[IV],[LIVMFYW](2)-{NLPA}-{T}-G-D-[NH]-{PIEW}-x(2)-[SND]-x(2)-[SG],R-I-A-R-N-[TQ]-x(2)-[LIVMFY](2)-x-[EQH]-E-x(4)-[KRN]-x(2)-D-P-x-[GSA]-G-S,L-[IV]-A-H-[STACH]-Y-[STV]-[RT]-Y-[LIVM]-G,[GA]-G-x-G-D-[TV]-[LT]-[STA]-G-x-[LIVM],[RKQGTF]-x(2)-G-N-[SA]-[LIVF]-x-[VIP]-x-[LVMT]-x(3)-[LIVM]-x(3)-[LIVM],[LIVMA]-{R}-E-G-[DN]-S-A-{F}-[STAG],"[AG]-[RK]-[LI]-x(1,2)-[LIV]-[FY]-E-x(2)-P-[LIVM]-[GSA]",C-x-{P}-C-{C}-x-C-{CP}-x-{C}-C-[PEG],E-x-[LIVM]-N-[ST]-[SA]-[LIV]-E-x(2)-V-D,"[GS]-x(4)-[LIVMT]-x(4)-[LIVMF]-x(2)-[CSAM]-[LMFY]-x(6)-[STC]-x(4,5)-[PAC]-x-[LIVMF]-x-[LIVMF]-x(8)-C-x(1,2)-[CH]",[LIVMFYC]-x-[HY]-x-D-[LIVMFY]-K-x(2)-N-[LIVMFYCT](3),[IV]-x-D-S-[GAS]-[GASC]-[GAST]-[GA]-T,[IV]-x-[IV]-[SA]-T-[NQ]-M-A-G-R-G-x-D-I-x-L,H-[STAG]-{ADNV}-{VGFI}-{YAR}-[LIVME]-{SDEP}-x-[LIVMFYW]-P-[FYW],[AG]-G-G-x-G-[STKA]-x-L-x(2)-L-[TA]-x(3)-[AST]-x-P-[AS]-[LV],D-[KRSTGANQFYW]-x(3)-E−[KRAQ]-x-[RKQD]-[GC]-[IVMK]-[ST]-[IV]-x(2)-[GSTACKRNQ],G-[AM]-G-[AR]-Y-[LIVM]-C-G-[DE](2)-[STA](2)-[LIM](2)-[END]-S,[LIVMFYC]-[SA]-[SAPGLVFYKQH]-G-[DENQMW]-[KRQASPCLIMFW]-[KRNQSTAVM]-[KRACLVM]-[LIVMFYPAN]-{PHY}-[LIVMFW]-[SAGCLIVP]-{FYWHP}-{KRHP}-[LIVMFYWSTA],[SA]-x-[RK]-x-Q-[LIVMT]-Q-E−[RNAK]-[LIM]-[TSNV],F-[IVFY]-G-[LM]-M-[G>],[LIVMFW]-x(2)-H-x-H-[DN]-D-x-G-x-[GAS]-x-[GASLI],[KRHGTCVN]-[VT]-[LIVMF]-[LIVMC]-R-x-D-x-N-[SACV]-P,"[YW]-x-[STKV]-x-[KR]-[NSKQ]-x(3,4)-[PATQS]-x(1,2)-[LIVMF]-[EAQVSIT]-x(2)-K-[FYH]-[CSD]","[LIVAMSFT]-x(3)-[GAHDVSI]-x-[GSAIVCT]-R-[LIVMCAFST]-[DE]-[LIVMFAYGT]-[LIVMFAR]-x(7,12)-[LIVWCAF]-x-[EK]-[LIVAPMT]-N-[STPA]-x-P-[GA]",[ST]-[DM]-H-[LIC]-x(2)-[FA]-[LIY]-[EQK]-R-x(2)-[QNKA],[DET]-[LIVMTA]-{NSYL}-{RPFC}-[LIVM]-[LIVMSTAG]-[SAG]-[LIVMSTAG]-H-[STA]-[LIVMFY],G-x(5)-E-x(4)-[TAGCV]-[LIVMACF]-x-R-[EL]-[LIVMFGSTA]-x-[EA]-E-x-[GNDTHR],[LIVM]-x-K-[FY]-G-G-[ST]-[SC]-[LIVM],[LIVMSTAC]-[LIVMFYWSTAGC]-[LIMSTAG]-[LIVMSTAGC]-x(2)-[DN]-x-{P}-[LIVMWSTAC]-{DP}-[LIVMFSTAG]-W-[DEN]-[LIVMFSTAGCN],"[EKH]-[LHVI]-x(9,10)-[IVNLR]-x(3)-[LIV]-x(6)-G-D-x(2)-E-N-[GSA]-x-Y″,D-{W}-[DNS]-{ILVFYW}-[DENSTG]-[DNQGHRK]-{GP}-[LIVMC]-[DENQSTAGC]-x(2)-[DE]-[LIVMFYW],"D-[ST]-[FY]-[RP]-[KHQ]-x(7,8)-[FYWD]-[ST]-[FYW](2)","[LIFAT]-{IL}-x(2)-W-x(2,3)-[PE]-x-{VF}-[LIVMFY]-[DENQS]-[STA]-[AV]-[LIVMFY]",E-x-G-G-P-x(2)-[GA]-x-G-C-[AG]-G,[FYIV]-{ND}-[FYVG]-[LIVM]-D-[LIVMF]-x-[STA]-K-x-{K}-[FY],[LIVF]-{LV}-x-[GANQK]-[NLG]-[SA]-[GA]-[TAI]-[STAGV]-{N}-R-x-[LIVMFYAT]-x-[GSTAP],C-x(2)-D-[LIVM]-x(6)-[ST]-x(4)-S-[HYR]-[HQ],[LIVMFGA]-E−[LIMSTAC]-[GS]-G-[KNLM]-[SADN]-[TAPFV],"H-x(4,5)-F-[LIVMFTP]-x-[FW]-H-R-x(2)-[LVMT]-x(3)-E″,"[LIVMFR]-x-[GSTACQI]-[LIVMF]-x(1,2)-[GSTALVM]-x(0,1)-[GSN]-[LIVMFY]-x-[LIVM]-x(4)-[DEN]-x-[TS]-[PS]-x-[PA]-[STCHF]-[DN]",[GDN]-[DEQTR]-x-[LIVMFY]-x(2)-[LIVM]-x-[AIV]-M-K-[LVMAT]-x(3)-[LIVM]-x-[SAV],"G-[LIVMFY]-x(2)-[LIVMFY]-x-[LIVM]-D-[DF]-x(1,2)-W-x(3,7)-[RV]-[DNSF]",[LIVMFY](3)-x-G-[DEQ]-[STE]-G-[STAV]-G-K-x(2)-[LIVMFY],[LIVM]-x-D-{EK}-[EDNTY]-[DG]-[RKHDENQ]-x-[LIVM]-x-{E}-{Q}-x(2)-Y-x-[LIVM],[PSA]-[LQ]-x-E−[YF]-Y-[LIVM](2)-[DE]-x-[FYWHN],D-x-L-G-D-V-V-C-G-G-F-[AGSP]-x-P,W-[QKR]-[NSD]-[SA]-[LIV]-R-H,"C-x(1,4)-C-[GSANHK]-x(1,2)-[IVML]-x(7,11)-R-[GSANPVLMT]-x(2)-[FYWIL]-C-x(2)-C-Q″,[LIVMT]-[RK]-[LIVM]-G-[LIVM]-G-x-G-[SRK]-[LIVMAT]-C-x-T,"[GSTA]-[KR]-x(6)-G-x-[LIVMT]-x(2)-[NQSCH]-x(1,3)-[LIVFCA]-x(3)-[LIV]-[DENQ]-x(7)-[LMT]-x(2)-G-x(2)-[GS]",G-[IVT]-[LVAC](2)-[IVT]-D-[DE]-[FL]-[DNST],[LIVM]-[PAIV]-[KR]-[ST]-{EPQG}-{RFI}-x(2)-R-{SVAF}-x-[GSTAEQK]-[NSL]-x-{LVRI}-[LIVMFA],[PG]-x-[GS]-C-[GA]-E−[EQ]-x-[LIVM],[FY]-P-S-[AGMS]-C-G-K-T-[NS],"[STAIV]-[PQDEL]-[DE]-[LIV]-[LIVTA]-Q-x-[STAV]-[LIVMFYC]-[LIVMAK]-x-[GSTAIV]-[LIMFYWQ]-x(12,14)-[STAP]-[FYW]-[LIF]-x(2)-[IV]",L-I-G-D-D-E-H-x-W-x-[DEPKVNA]-x-[GVS]-[IV]-x-N,G-x-[DN]-F-x-K-x-D-E,S-E−[HN]-x-[LIVM]-x(4)-[FYH]-x(2)-E−[LIVMGA]-H-[LIVMFA](2),[LIVMFY]-x(2)-G-x(2)-Y-x-F-x-K-x(2)-[SN]-[STAV]-[LIVMFYW]-V,[IVLC]-M-[LIVM]-G-Y-S-D-S-x-K-[DF]-[STAG]-G,[GSAIVK]-{FE}-[FYW]-x-[LIVMF]-x(2)-{K}-x-[NHG]-[FY]-[DE]-x-[LIVMFY]-[LIVM]-{N}-{G}-[LIVMAKR],[LIFV]-x(6)-[LIF]-[LIVF]-x-[GSDE]-[GSTADNPE]-[PASG]-x(2)-R-R-x-[FYW]-[LIVMF]-[DN],C-x-C-x(2)-{V}-x(2)-G-{C}-x-C,G-R-x-N-[LIV]-I-G-[DE]-H-x-D-Y,[DEQGSTALMKRH]-[LIVMFYSTAC]-[GNQ]-[LIVMFYAG]-[DNEKHS]-S-[LIVMST]-{PCFY}-[STAGCPQLIVMF]-[LIVMATN]-[DENQGTAKRHLM]-[LIVMWSTA]-[LIVGSTACR]-{LPIY}-{VY}-[LIVMFA],"[GSA]-x(2,6)-[LIVMSCP]-x-{N}-[LIVMF]-[DNS]-[LIVMCA]-G-G-G-[LIVMFY]-[GSTPCEQ]",[LIVMFC]-G-D-[GSANQ]-x-N-D-x(3)-[LIMFY]-x(2)-[AV]-x(2)-[GSCP]-x(2)-[LMP]-x(2)-[GAS],[LIVM]-x-[LIVMT]-x(2)-G-C-x(3)-C-[STAN]-[FY]-C-x-[LIVMT]-x(4)-G,C–P-x-C-[DE]-x-[GS](2)-x-C-x-L-Q,[STAGDN]-Y-x-Y-E−{AV}-{L}-[DE]-[KR]-[STAGCI],"[LIVMF]-[QKRHSA]-{E}-x-[LIVMAC]-x(5,6)-[LIVMW]-[RKAYF]-x-[STACIVMF]-[PV]-{LG}-[LIVMF]-x-[FYI]-x(2)-D″,V-[ASV]-[TS]-[IVLA]-[RQ]-[AGS]-[LIM]-[KER]-x-[HN]-[GAS]-[GLKD],[SGATV]-{D}-x(2)-[LIVMA]-R-[LIVMA]-x-[FW]-H-{V}-[SAC],[NQH]-x(4)-P-x-H-x(2)-[SAG]-x(11)-[SAGC]-x-H-[SAG](2),S-[DN]-[GA]-D-[LIVAP]-[LIVAG]-x-H-[STAC]-x(2)-[DNT]-[SAG]-x(2)-[SGA],[GSTADE]-[KREQSTIV]-x-{EPRK}-{VPGL}-x-[KRDN]-S-[LIVMF](2)-{EVPL}-[LIVM]-{EATN}-x-[LIVM]-[GADE],G-x(2)-[LIVWPQT]-x(3)-[GACST]-C-[GSTAM]-[LIMPTA]-C-[LIMV]-[GA],[DNSK]-[PSTV]-x-[SAG](2)-[GD]-D-x(3)-[SAGV]-[AG]-[LIVMFYA]-[LIVMSTAP],[STN]-x(2)-[DENQ]-[LIVMT]-[GAS]-x(4)-[LIVMF]-[PSTG]-x(3)-[LIVMA]-x-[NQR]-[LIVMA]-[EQH]-x(3)-[LIVMFWK]-x(2)-[LIVM],[DEQSKN]-x-[LIVMF]-[SA]-[LIVMF]-G-[ST]-N-D-[LIVM]-x-Q-[LIVMFYGT]-[STALIV]-[LIVMFY]-[GAS]-x(2)-R,Y-[KR]-G-[AS]-[AE]-Y,"[GSTENA]-x-[LIVMF]-P-x(5)-[LIVMW]-x(2,3)-[LI]-[PAS]-G-[IV]-[GA]-x(3)-[GAC]-x(2,3)-[LIVMA]-x(1,2)-[GSALVI]-[LIVMFYW]-[GANKD]","P-x(0,2)-[GSTAN]-[DENQGAPK]-x-[LIVMFP]-[HT]-[LIVMYAC]-G-[HNTG]-[LIVMFYSTAGPC]",L-x(3)-[GRS]-[LIVY]-x(2)-[STA]-x(2)-G-x(2)-G-G-[FYIV]-x-[LIF],[GSDN]-[DEQHKM]-x(2)-L-x(3)-[SAG](2)-G-G-x-G-x(4)-Q-x(2)-[KRS],[LIVMF]-[LIVMFY]-[DN]-[LIVMFS]-G-[GSH]-[GS]-[AST]-x(3)-[ST]-[LIVM]-[LIVMFC],G-G-x-C-[LIVA]-x(2)-G-C-[LIVM]-P,[QDE]-x-{P}-G-[GS]-x-G-[LIVMFY]-x(2)-[DEN]-x(4)-[KR]-x(3)-[DEN],[LIVMCA]-[LIVM](2)-[LITF]-[LITN]-G-G-T-G-x(4)-D,"[LIVNS]-x-{L}-[LIVMFA]-x-C-x-[STAGCDNH]-C-x(3)-[LIVFG]-{LV}-x(2)-[LIV]-x(9,11)-[IVA]-x-[LVFYS]",[LIVMFYWCA]-[LIVMFYW](2)-D-G-[FYI]-P-R-x(3)-[NQ],[QGF]-[WLCF]-G-D-E−[GA]-K-[GA],D-K-T-G-T-[LIVM]-[TI],R-[LF]-G-D-P-E-x-[EQIM],[DENSK]-x-[LIVMDET]-x(3)-[LIVMFTA](2)-x(6)-G-K-[KR]-x(5)-[LIVMF]-[LIVMFC]-x(2)-[STAC],[LVSAT]-[LIVA]-x(2)-[LIVMT]-[PSD]-x(3)-[LI]-[LIVMT]-[LIVMST]-E-T-D-x-P,Y-[LIVAC]-R-[VA]-S-[ST]-x(2)-Q,G-[DE]-x(2)-[LIVM]-{E}-x-{V}-[LIVM]-[DT]-R-[LIVM]-[GSA],[FY]-x(2)-[STCNLVA]-x-[FV]-H-[RH]-[LIVMNS]-[LIVM]-x(2)-F-[LIVM]-x-Q-[AGFT]-G,W-x(4)-[YF]-D-x(3)-[DN]-[LIVMFYT]-[LIVMFY](3)-x(2)-G-x(2)-[STAG]-[PVT],"[LIVMA]-x-[LIVM]-M-[ST]-[VS]-x-P-x(3)-[GN]-Q-x(0,1)-[FMK]-x(6)-[NKR]-[LIVMC]",[LIV]-[LIVMGSTC]-[DET]-[RH]-[FYHCS]-x(2)-S-[GSTNP]-x-[AVC]-[FY]-[STANQ],E−[ST]-[EA]-R-E-A-[RK]-x-[LI],[EQ]-[YF]-A-[LIVM]-x(2)-[LIVM]-x(4)-[LIVMF](3)-x-G-H-x(2)-C-G,[RK]-x-[STA]-x(2)-S-x-C-Y-[SL],[GSTAP]-x(2)-[DNEQA]-[LIVM]-[GSA]-x(2)-[LIVMFYT]-[GAN]-[LIVMST]-[ST]-x(6)-R-[LIVT]-x(2)-[LIVM]-x(3)-G,[LIVMFY](2)-D-[STA]-H-x-H-[LIVMFP]-[DN],"[GA]-x(1,2)-[DE]-x-Y-x-[STAPV]-x-C-[NKR]-x-[CH]-[LIVMFYWH]",K-x-[AV]-x(4)-G-x(2)-[LIVT]-x-V-P-x(2)-[LIVC]-x(2)-[GD],"[MFY]-x-G-H-G-[LIVMC]-[GSHN]-x(3)-H-x(4)-[LIVM]-x(1,2)-[HN]-[YWVHF]",P-x(2)-R-G-[STAIV](2)-x-N-[APK]-x-[DE]
  A complete list of 1739 GO terms (of molecular function domain only) assigned as target labels for forming Train/Test datasets is shown below:

GO:0047529,GO:0008565,GO:0015254,GO:0017040,GO:0005534,GO:0003906,GO:0048307,GO:0008926,GO:0047462,GO:0004396,GO:0050421,GO:0002020,GO:0052856,GO:0008800,GO:0003989,GO:0001217,GO:0003690,GO:0033752,GO:0005355,GO:0016677,GO:0016208,GO:0043199,GO:0047985,GO:0004802,GO:0003896,GO:0016830,GO:0031071,GO:0052621,GO:0047943,GO:0004639,GO:0004747,GO:0046428,GO:0043142,GO:0015094,GO:0018457,GO:0008673,GO:0016855,GO:0032267,GO:0004130,GO:0008774,GO:0046526,GO:0004721,GO:0018506,GO:0008885,GO:0036094,GO:0102009,GO:0008705,GO:0106099,GO:0015423,GO:0036355,GO:0004055,GO:0032131,GO:0008745,GO:0050660,GO:0004672,GO:0015112,GO:0005516,GO:0008904,GO:0008138,GO:0015344,GO:0000310,GO:0000210,GO:0004109,GO:0008974,GO:0015426,GO:0047806,GO:0044183,GO:0036220,GO:0052858,GO:0061798,GO:0046564,GO:0004615,GO:0102162,GO:0016403,GO:0004854,GO:0010333,GO:0047595,GO:0005506,GO:0030337,GO:0015595,GO:0047429,GO:0047761,GO:0004477,GO:0046857,GO:0061503,GO:0042578,GO:0098519,GO:0102568,GO:0018455,GO:0070968,GO:0008907,GO:0043822,GO:0004823,GO:0048365,GO:0015288,GO:0008795,GO:0015623,GO:0016168,GO:0015293,GO:0003746,GO:0004411,GO:0016774,GO:0047862,GO:0004180,GO:0009384,GO:0004072,GO:0005215,GO:0042289,GO:0008980,GO:0004832,GO:0004156,GO:0047702,GO:0051537,GO:0070026,GO:0043140,GO:0004473,GO:0008710,GO:0004640,GO:0004739,GO:0003862,GO:1904047,GO:0003910,GO:0019003,GO:0016799,GO:0016651,GO:0030272,GO:0015420,GO:0008802,GO:0008902,GO:0015099,GO:0018467,GO:0043792,GO:0030983,GO:0140078,GO:0017136,GO:0016868,GO:0004129,GO:0047324,GO:0050020,GO:0052912,GO:0004359,GO:0016615,GO:0031420,GO:0008137,GO:0043022,GO:0004619,GO:0050262,GO:0015095,GO:0016410,GO:0009389,GO:0047330,GO:0004113,GO:0004497,GO:0019146,GO:0016491,GO:0004812,GO:0003908,GO:0047112,GO:0008869,GO:0008713,GO:0047348,GO:0031177,GO:0009486,GO:0016298,GO:0016841,GO:0004160,GO:0004328,GO:1901973,GO:0004821,GO:0034700,GO:0008725,GO:0050001,GO:0016620,GO:0070569,GO:0102921,GO:0005085,GO:0036054,GO:0097367,GO:0050301,GO:0015232,GO:0003842,GO:0004222,GO:0016436,GO:0016775,GO:0008690,GO:0008237,GO:0050319,GO:0018452,GO:0004065,GO:0103046,GO:0008833,GO:0043873,GO:0008198,GO:0008853,GO:0008409,GO:0102008,GO:0017091,GO:0008964,GO:0004066,GO:0019826,GO:0035439,GO:0036222,GO:0015643,GO:0052821,GO:0004590,GO:0045145,GO:0031419,GO:0005345,GO:0043682,GO:0005384,GO:0004750,GO:0008324,GO:0043885,GO:0051745,GO:0003935,GO:0008073,GO:0050279,GO:0036200,GO:0017137,GO:0008758,GO:0016880,GO:0008413,GO:0052908,GO:0003874,GO:0033848,GO:0008134,GO:0016810,GO:0032448,GO:0070181,GO:0050256,GO:0000155,GO:0008047,GO:0005507,GO:0016852,GO:0004594,GO:0050163,GO:0016165,GO:0015616,GO:0004386,GO:0052618,GO:0008968,GO:0015413,GO:0004842,GO:0033958,GO:0030248,GO:0008556,GO:0034335,GO:0030170,GO:0043904,GO:0008121,GO:0008872,GO:0008772,GO:0004674,GO:0047389,GO:0102252,GO:0019825,GO:0018799,GO:0106026,GO:0050223,GO:0000287,GO:0004644,GO:0003697,GO:0034025,GO:0004748,GO:0009009,GO:0047974,GO:0008483,GO:0043365,GO:0016655,GO:0009028,GO:0051287,GO:0070566,GO:0016758,GO:0047067,GO:0008720,GO:0047150,GO:0002134,GO:0009012,GO:0004340,GO:0004503,GO:0018805,GO:0004399,GO:0046820,GO:0005344,GO:0015609,GO:0019150,GO:0017151,GO:0018597,GO:0008879,GO:0032549,GO:0097351,GO:0047917,GO:0008478,GO:0015184,GO:0047121,GO:0004779,GO:0018710,GO:0050897,GO:0103039,GO:0009046,GO:0035888,GO:0016433,GO:0004310,GO:0047900,GO:0070905,GO:0061602,GO:0042626,GO:0044212,GO:0008768,GO:0050385,GO:0045148,GO:0015436,GO:0016882,GO:0000179,GO:0032575,GO:0008970,GO:0008835,GO:0035539,GO:0047840,GO:0022857,GO:0004829,GO:0004452,GO:0047113,GO:0008888,GO:0102299,GO:0004526,GO:0003848,GO:0008177,GO:0034611,GO:0008080,GO:0008097,GO:0052739,GO:0001108,GO:0061599,GO:0008819,GO:0033613,GO:0018818,GO:0015462,GO:0047652,GO:0004131,GO:0003868,GO:0003863,GO:0033748,GO:0016740,GO:0047972,GO:0008876,GO:0008792,GO:0033677,GO:0005354,GO:0008934,GO:0033204,GO:0050484,GO:0004176,GO:0008375,GO:0018456,GO:0030507,GO:0018802,GO:0035731,GO:0004040,GO:0050515,GO:0008787,GO:0050292,GO:0008878,GO:0015438,GO:0004788,GO:0047545,GO:0061693,GO:0004377,GO:0016778,GO:0016798,GO:0050140,GO:0000822,GO:0016207,GO:0016795,GO:0015021,GO:0047553,GO:0047154,GO:0004089,GO:0043752,GO:0015562,GO:0004325,GO:0016836,GO:0051575,GO:0102391,GO:0000175,GO:0016989,GO:0008716,GO:0047693,GO:0003856,GO:0047844,GO:0008553,GO:0008779,GO:0004824,GO:0004475,GO:0047929,GO:0035438,GO:0015667,GO:0090729,GO:0008296,GO:0060698,GO:0008863,GO:0051063,GO:0008986,GO:0016879,GO:0015572,GO:0036422,GO:0004749,GO:0102908,GO:0051213,GO:0004527,GO:0008854,GO:0004532,GO:0036311,GO:0009045,GO:0016614,GO:0070406,GO:0052764,GO:0031072,GO:0004137,GO:0097100,GO:0016878,GO:0003922,GO:0034005,GO:0019899,GO:0015441,GO:0070538,GO:0004024,GO:0052693,GO:0050519,GO:0052680,GO:0044603,GO:0004482,GO:0004512,GO:0008263,GO:0004635,GO:0015078,GO:0000049,GO:0003977,GO:0004813,GO:0008234,GO:0047991,GO:0000309,GO:0102229,GO:0003779,GO:0008976,GO:0047075,GO:0008756,GO:0004614,GO:0018753,GO:0047584,GO:0050084,GO:0000150,GO:0009041,GO:0000166,GO:0043273,GO:0047609,GO:0030975,GO:0003729,GO:0047527,GO:0009030,GO:0004148,GO:0003917,GO:0043884,GO:0005518,GO:0050083,GO:0004494,GO:0018859,GO:0016979,GO:0051499,GO:0008320,GO:0009975,GO:0050695,GO:0016823,GO:0050470,GO:0016763,GO:0016627,GO:0008940,GO:0050518,GO:0043798,GO:0045300,GO:0008922,GO:0005319,GO:0030246,GO:0035312,GO:0051212,GO:0004149,GO:0004311,GO:0050560,GO:0052619,GO:0048502,GO:0047471,GO:0030145,GO:0052692,GO:0018861,GO:0047817,GO:0090612,GO:0004736,GO:0004806,GO:0018459,GO:0048039,GO:0034040,GO:0018800,GO:0003678,GO:0004081,GO:0017108,GO:0047465,GO:0004576,GO:0033739,GO:0047954,GO:0004412,GO:0009381,GO:0033999,GO:0008727,GO:0004400,GO:0004743,GO:0003904,GO:0030613,GO:0036356,GO:0008966,GO:0003755,GO:0003905,GO:0032357,GO:0004401,GO:0030234,GO:0046812,GO:0008108,GO:0008783,GO:0051907,GO:0009881,GO:0043726,GO:0001000,GO:0018648,GO:0047721,GO:0043750,GO:0004138,GO:0034459,GO:0004332,GO:0008738,GO:0008477,GO:0004556,GO:0035375,GO:0009007,GO:0003920,GO:0016817,GO:0061712,GO:0048038,GO:0050225,GO:0016154,GO:0017110,GO:0009038,GO:0061501,GO:0102264,GO:0043751,GO:0008463,GO:0004540,GO:0009017,GO:0043878,GO:0051787,GO:0102710,GO:0016301,GO:0018492,GO:0004765,GO:0016887,GO:0050192,GO:0004844,GO:0003924,GO:0071111,GO:0019158,GO:0015419,GO:0002094,GO:0019104,GO:0004015,GO:0047700,GO:0017113,GO:0001968,GO:0004136,GO:0004630,GO:0016767,GO:0036361,GO:0009024,GO:0009020,GO:0031176,GO:0008706,GO:0008695,GO:0051392,GO:0004715,GO:0070403,GO:0017168,GO:0003824,GO:0015208,GO:0047869,GO:0047334,GO:0035595,GO:0004825,GO:0016743,GO:0008757,GO:0008817,GO:0046983,GO:0001073,GO:0008763,GO:0003714,GO:0050572,GO:0008820,GO:0032843,GO:0050066,GO:0015093,GO:0004044,GO:0000900,GO:0052911,GO:0043880,GO:0004035,GO:0018706,GO:0016833,GO:0008685,GO:0032556,GO:0003826,GO:0047540,GO:0043906,GO:0047441,GO:0102040,GO:0015035,GO:0008199,GO:0004427,GO:0004001,GO:0050278,GO:0004856,GO:0005536,GO:0016209,GO:0016831,GO:0034480,GO:0004450,GO:0061594,GO:0015291,GO:0034618,GO:0008784,GO:0004071,GO:0004735,GO:0004316,GO:0004416,GO:0050531,GO:0005524,GO:0016625,GO:0008276,GO:0050510,GO:0000286,GO:0032450,GO:0070041,GO:0008171,GO:0018788,GO:0030570,GO:0000985,GO:0016805,GO:0018697,GO:0043023,GO:0017116,GO:0008852,GO:0050532,GO:0005525,GO:0046917,GO:0008796,GO:0008094,GO:0004592,GO:0004155,GO:0008659,GO:0008694,GO:0001406,GO:0070404,GO:0004525,GO:0051500,GO:0061598,GO:0004425,GO:0044374,GO:0047695,GO:0008127,GO:0047727,GO:0004520,GO:0034701,GO:0030697,GO:1990404,GO:0047419,GO:0004818,GO:0004106,GO:0008807,GO:0010855,GO:0034200,GO:0003984,GO:0000908,GO:0008235,GO:0008868,GO:0005471,GO:0004008,GO:0047611,GO:0031418,GO:0003996,GO:0050565,GO:0070011,GO:0008270,GO:0004781,GO:0004017,GO:0002953,GO:0046992,GO:0051015,GO:0008650,GO:0008815,GO:0009025,GO:0043907,GO:0004252,GO:0008740,GO:0052636,GO:0050194,GO:0050096,GO:0043531,GO:0008973,GO:0008428,GO:0016840,GO:0009029,GO:0050311,GO:0009037,GO:0043775,GO:0016779,GO:0008448,GO:0102007,GO:0030340,GO:0009883,GO:0004462,GO:0016675,GO:0004019,GO:0050242,GO:0008906,GO:0043856,GO:0102023,GO:0004817,GO:0004828,GO:0015169,GO:0015612,GO:0042380,GO:0008936,GO:0090586,GO:0008831,GO:0008765,GO:0019200,GO:0044600,GO:0008301,GO:0098848,GO:0033850,GO:0015080,GO:0030729,GO:0050110,GO:0070095,GO:0034001,GO:0009022,GO:0015162,GO:0043743,GO:0004574,GO:0016812,GO:0003960,GO:0003887,GO:0004145,GO:0004523,GO:0004397,GO:0047480,GO:0034353,GO:0015408,GO:0031409,GO:0008479,GO:0047770,GO:0043773,GO:0042803,GO:0004363,GO:0052833,GO:0050570,GO:0005385,GO:0003952,GO:0047059,GO:0052929,GO:0051082,GO:0008908,GO:0016866,GO:0008289,GO:0004488,GO:0034545,GO:0018838,GO:0050023,GO:0050394,GO:0016150,GO:0008901,GO:0016672,GO:0047443,GO:0050662,GO:0016725,GO:0008910,GO:0015489,GO:0047294,GO:0004324,GO:0004049,GO:0050308,GO:0033296,GO:0030151,GO:0004722,GO:0032561,GO:0050611,GO:0052577,GO:0047590,GO:0047725,GO:0046555,GO:0016152,GO:0061603,GO:0016966,GO:0004174,GO:0050270,GO:0050297,GO:0004636,GO:0004197,GO:0004725,GO:0051060,GO:0015418,GO:0004088,GO:0019203,GO:0016706,GO:0050487,GO:0050622,GO:0004346,GO:0050555,GO:0008484,GO:0004087,GO:0061799,GO:0003684,GO:0004356,GO:0008677,GO:0046556,GO:0004816,GO:0047120,GO:0004417,GO:0016262,GO:0003921,GO:0008953,GO:0004016,GO:0008937,GO:0015926,GO:0016832,GO:0048031,GO:0016783,GO:0008884,GO:0015188,GO:0004751,GO:0047133,GO:0047942,GO:0047451,GO:0032067,GO:0009374,GO:0102481,GO:0008766,GO:0047134,GO:0008730,GO:0003677,GO:0004038,GO:0033926,GO:0036423,GO:0097718,GO:0004612,GO:0004357,GO:0034979,GO:0004528,GO:0004585,GO:0004115,GO:0003913,GO:0015666,GO:0044877,GO:0008962,GO:0004558,GO:0004159,GO:0004857,GO:0044715,GO:0043621,GO:0089715,GO:0034024,GO:0102210,GO:0030295,GO:0004833,GO:0016405,GO:0047473,GO:0004638,GO:0018756,GO:0004364,GO:0004151,GO:0033883,GO:0030612,GO:0000907,GO:0030492,GO:0018620,GO:0043814,GO:0003850,GO:0047828,GO:0047395,GO:0032791,GO:0008998,GO:0015658,GO:0050583,GO:0003864,GO:0008949,GO:0017124,GO:0032136,GO:0042242,GO:0005034,GO:0047971,GO:0043818,GO:0008764,GO:0004783,GO:0003680,GO:0019144,GO:0031956,GO:0050661,GO:0008742,GO:0004422,GO:0008430,GO:0004053,GO:0004647,GO:0003987,GO:0000976,GO:0004849,GO:0004601,GO:0102527,GO:0120147,GO:0019165,GO:0043908,GO:0043866,GO:0030371,GO:0009000,GO:0004181,GO:0019136,GO:0000774,GO:0004618,GO:0004591,GO:0090614,GO:0071949,GO:0015430,GO:0046993,GO:0004633,GO:0005504,GO:0052737,GO:0009927,GO:0015444,GO:0015192,GO:0020037,GO:0015450,GO:0019787,GO:0016849,GO:0018525,GO:0042623,GO:0004177,GO:0004521,GO:0046429,GO:0050355,GO:0019134,GO:0042301,GO:0008649,GO:0008771,GO:0008890,GO:0102276,GO:0018685,GO:0018551,GO:0043565,GO:0008996,GO:0035529,GO:0004797,GO:0047753,GO:0016861,GO:0031249,GO:0030772,GO:0004150,GO:0015087,GO:0016903,GO:0042954,GO:0070180,GO:0004493,GO:0047456,GO:0016746,GO:0004061,GO:0004076,GO:0004589,GO:0016891,GO:0004132,GO:0043802,GO:0008798,GO:0047868,GO:0004654,GO:0017111,GO:0004020,GO:0016782,GO:0003886,GO:0004641,GO:0070040,GO:0008995,GO:0004645,GO:0004045,GO:0047304,GO:0008777,GO:0004161,GO:0004530,GO:0050567,GO:0031459,GO:0000014,GO:0008841,GO:0003934,GO:0047257,GO:0004565,GO:0008865,GO:0047111,GO:0043722,GO:0003883,GO:0015445,GO:0004756,GO:0071161,GO:0051996,GO:0008941,GO:0008889,GO:0004056,GO:0004737,GO:0004392,GO:0001530,GO:0003972,GO:0031402,GO:0018468,GO:0008741,GO:0061605,GO:0004553,GO:0004003,GO:0047694,GO:0047931,GO:0050439,GO:0043916,GO:0015668,GO:0052689,GO:2001070,GO:0008559,GO:0019807,GO:0004371,GO:0048029,GO:0035485,GO:0008824,GO:0070336,GO:0016984,GO:0016018,GO:0015097,GO:0070006,GO:0030976,GO:0016881,GO:0008828,GO:0019002,GO:0070733,GO:0010698,GO:0032217,GO:0047388,GO:0043364,GO:0004476,GO:0000156,GO:0005381,GO:0004623,GO:0008236,GO:0030552,GO:0002135,GO:0032564,GO:0016780,GO:0071160,GO:0052928,GO:0008928,GO:0051989,GO:0008509,GO:0097098,GO:0019904,GO:0001150,GO:0043177,GO:0034015,GO:0015592,GO:0051699,GO:0008703,GO:0051538,GO:0015412,GO:0001216,GO:0008201,GO:0016671,GO:0047445,GO:0004784,GO:0004799,GO:0004789,GO:0047585,GO:0050545,GO:0030955,GO:0016854,GO:0050009,GO:0008887,GO:0004489,GO:0016773,GO:0019829,GO:0004826,GO:0042931,GO:0004815,GO:0033786,GO:0016896,GO:0043715,GO:0004637,GO:0004413,GO:0008700,GO:0061711,GO:0009982,GO:0033972,GO:0052914,GO:0008670,GO:0004820,GO:0003727,GO:0018522,GO:0052857,GO:0004333,GO:0045152,GO:0004451,GO:0004337,GO:0050093,GO:0035496,GO:0003983,GO:0018858,GO:0030385,GO:0046566,GO:0004550,GO:0102567,GO:0004845,GO:0004372,GO:0000121,GO:0009027,GO:0016765,GO:0050060,GO:0018169,GO:0051266,GO:0015226,GO:0046565,GO:0019147,GO:0017118,GO:0050097,GO:0004355,GO:0047482,GO:0033898,GO:0003861,GO:0004819,GO:0008233,GO:0003916,GO:0050418,GO:0102573,GO:0034000,GO:0008776,GO:0003730,GO:0015662,GO:0102130,GO:0043141,GO:0015417,GO:0097077,GO:0070628,GO:0070063,GO:0008816,GO:0009032,GO:0102480,GO:0016874,GO:0008186,GO:0004070,GO:0046583,GO:0051065,GO:0050032,GO:0047474,GO:0005275,GO:0047475,GO:0008801,GO:0045340,GO:0045735,GO:0050299,GO:0003723,GO:0008697,GO:0015638,GO:0015437,GO:0042586,GO:0003994,GO:0008942,GO:0001017,GO:0015267,GO:0050587,GO:0015611,GO:0009039,GO:0047488,GO:0008882,GO:0005324,GO:0033727,GO:0018798,GO:0048474,GO:0008173,GO:0003941,GO:0016838,GO:1990238,GO:0003918,GO:0015620,GO:0008979,GO:0000405,GO:0004190,GO:0003735,GO:0004798,GO:0035250,GO:0050074,GO:0015411,GO:0016747,GO:0018814,GO:0019177,GO:0016776,GO:0015086,GO:0050393,GO:0034039,GO:0070273,GO:0050538,GO:0004470,GO:0003954,GO:0019862,GO:0015407,GO:0005315,GO:0043721,GO:0004830,GO:0047143,GO:0008948,GO:0008744,GO:0047878,GO:0004478,GO:0004458,GO:0008551,GO:0016213,GO:0042277,GO:0050467,GO:0003909,GO:0003959,GO:0010181,GO:0009496,GO:0140032,GO:0016987,GO:0102313,GO:0102131,GO:0008972,GO:0050286,GO:0061473,GO:0004655,GO:0034004,GO:0019154,GO:0016462,GO:0047343,GO:0000984,GO:0008312,GO:0033558,GO:0008963,GO:0005304,GO:0015036,GO:0015238,GO:0046872,GO:0050564,GO:0019164,GO:0045156,GO:0004517,GO:0008821,GO:0016437,GO:0016407,GO:0005198,GO:0003747,GO:0008728,GO:0009036,GO:0052927,GO:0004141,GO:0008494,GO:0008781,GO:0030350,GO:0009378,GO:0033982,GO:0003743,GO:0015292,GO:0048037,GO:0008657,GO:0043783,GO:0047548,GO:0009055,GO:0008860,GO:0042314,GO:0008254,GO:0004632,GO:0016597,GO:0042834,GO:0016857,GO:0019239,GO:0008092,GO:0044716,GO:0004112,GO:0004075,GO:0047808,GO:0050490,GO:0070204,GO:0005102,GO:0004588,GO:0008804,GO:0033763,GO:0008909,GO:0005047,GO:0032567,GO:0047490,GO:0000104,GO:0008993,GO:0050473,GO:0052740,GO:0043765,GO:0019143,GO:0003939,GO:0016722,GO:0003879,GO:0103012,GO:0046810,GO:0001047,GO:0042781,GO:0008534,GO:0003724,GO:0008081,GO:0004810,GO:0004827,GO:0004077,GO:0016668,GO:0140035,GO:0016639,GO:0050338,GO:0004808,GO:0016709,GO:0008965,GO:0015591,GO:0050021,GO:0035598,GO:0061710,GO:0008641,GO:0052869,GO:0046933,GO:0047831,GO:0004474,GO:0003847,GO:0008443,GO:0045127,GO:0043532,GO:2001065,GO:0008531,GO:0004063,GO:0004555,GO:0047777,GO:0017150,GO:1990002,GO:0016791,GO:0003899,GO:0033719,GO:0046522,GO:0016811,GO:0043874,GO:0043546,GO:0047798,GO:0015594,GO:0016002,GO:0036397,GO:0002935,GO:0046873,GO:0004298,GO:0004152,GO:0043755,GO:0004326,GO:0036380,GO:0004634,GO:0008441,GO:0018628,GO:0043136,GO:0016888,GO:0043737,GO:0008877,GO:0052832,GO:0002057,GO:0008704,GO:0034028,GO:0008743,GO:0003914,GO:0046961,GO:0018564,GO:0016992,GO:0046316,GO:0036524,GO:0052868,GO:0047789,GO:0050218,GO:0051536,GO:0103025,GO:0033978,GO:0046523,GO:0003951,GO:0004518,GO:0008408,GO:0070402,GO:0050053,GO:0008668,GO:0004419,GO:0018523,GO:0004175,GO:0015446,GO:0047837,GO:0050138,GO:0004712,GO:0102121,GO:0032296,GO:0010436,GO:0031404,GO:0102561,GO:0005216,GO:0102132,GO:0016772,GO:0008686,GO:0030973,GO:0015190,GO:0004385,GO:0004803,GO:0008834,GO:0008294,GO:0004467,GO:0008106,GO:0008810,GO:0019172,GO:0004349,GO:0004831,GO:0052906,GO:0000030,GO:0051539,GO:0031218,GO:0004048,GO:0016803,GO:0016161,GO:0004140,GO:0015614,GO:0016813,GO:0035599,GO:0004642,GO:0004652,GO:0004724,GO:0031403,GO:0008452,GO:0016151,GO:0046570,GO:0010334,GO:0033676,GO:0050480,GO:0016869,GO:0004321,GO:0016163,GO:0008982,GO:0004860,GO:0004370,GO:0042802,GO:0047434,GO:0004822,GO:0016787,GO:0035446,GO:0003756,GO:0003681,GO:0052822,GO:0003872,GO:0047435,GO:0070290,GO:0008168,GO:0004805,GO:0008893,GO:0004673,GO:0008832,GO:0016853,GO:0102302,GO:0032794,GO:0070573,GO:0042959,GO:0045158,GO:0004424,GO:0008683,GO:0008026,GO:0071972,GO:0003852,GO:0047238,GO:0004022,GO:0015415,GO:0016616,GO:0015049,GO:0004595,GO:0000721,GO:0051903,GO:0047683,GO:0000986,GO:0102560,GO:0003688,GO:0003877,GO:0043175,GO:0016892,GO:0008143,GO:0052855,GO:0046914,GO:0016818,GO:0016757,GO:0008666,GO:0018817,GO:0008311,GO:0009019,GO:0016701,GO:0071667,GO:0047711,GO:0043169,GO:0046421,GO:0009035,GO:0003993,GO:0004559,GO:0042577,GO:0008083,GO:0008379,GO:0050492,GO:0003991,GO:0042132,GO:0016149,GO:0033785,GO:0000703,GO:0016679,GO:0016628,GO:0005342,GO:0008253,GO:0005509,GO:0008867,GO:0050228,GO:0004775,GO:0004738,GO:0008959,GO:0004000,GO:0050071,GO:0004658,GO:0103047,GO:0031216,GO:0000400,GO:0004659,GO:0016682,GO:0052923,GO:1904680,GO:0016730,GO:0008892,GO:0004631,GO:0047516,GO:0033592,GO:0052873,GO:0102127,GO:0001072,GO:0008967,GO:1990594,GO:0070497,GO:1901359,GO:0043815,GO:0008310,GO:0003676,GO:0043716,GO:0008470,GO:0046982,GO:0019534,GO:0042279,GO:0005291,GO:0008987,GO:0016669,GO:0004534,GO:0034008,GO:0050081,GO:0015416,GO:0030341,GO:0050483,GO:0003999,GO:0008737,GO:0004143,GO:0016829,GO:0003953,GO:0008663,GO:0097216,GO:0004157,GO:0015234,GO:0003725,GO:0097163,GO:0043897,GO:0034909,GO:0008898,GO:0098531,GO:0018583,GO:0002196,GO:0052751,GO:0016636,GO:0097063,GO:0019843,GO:0004604,GO:0002058,GO:0052717,GO:0008672,GO:0015645,GO:0047086,GO:0004004,GO:0050415,GO:0102026,GO:0015343,GO:0003700,GO:0050559,GO:0070025,GO:0015439,GO:0048027,GO:0002161,GO:0018662,GO:0004127,GO:0033863,GO:0008897,GO:0033743,GO:0019156,GO:0052916,GO:0003938,GO:0015633,GO:0004042,GO:0042410,GO:0004713,GO:0106029,GO:0004536,GO:0008146,GO:0004329,GO:0000701,GO:0042895,GO:0050347,GO:0008701,GO:0004814,GO:0030604,GO:0046537,GO:0004515,GO:0000062,GO:0046870,GO:0051116,GO:0003911,GO:0034336,GO:0004096,GO:0016702,GO:0016788,GO:0008170,GO:0061733,GO:0032451,GO:0043899,GO:0004126,GO:0004471,GO:0050136,GO:1905576,GO:0004613,GO:0047489,GO:0015148,GO:0102300,GO:0035368,GO:0004335,GO:0015191,GO:0016851,GO:0004170,GO:0008994,GO:0018819,GO:0103117,GO:0008912,GO:0004484,GO:0008989,GO:0047577,GO:0008921,GO:0001727,GO:0046558,GO:0016790,GO:0043895,GO:0008671,GO:0022885,GO:0016835,GO:0047689,GO:0008927,GO:0051087,GO:0043168,GO:0005347,GO:0008759,GO:0008662,GO:0030769,GO:0018860,GO:0016041,GO:0050300,GO:0003963,GO:0015038,GO:0004029,GO:0004557,GO:0050043,GO:0004657,GO:0004309,GO:0009385,GO:0019213,GO:0004519,GO:0032553,GO:0019206,GO:0000034,GO:0052381,GO:0043024,GO:0009392,GO:0034057,GO:0004455,GO:0004529,GO:0047911,GO:0009014,GO:0047631,GO:0016530,GO:0047110,GO:0103053,GO:0018645,GO:0009678,GO:0033862,GO:0042888,GO:0046553,GO:0047400,GO:0016463,GO:0003919,GO:0033680,GO:0015424,GO:0004123,GO:0034027,GO:0018850,GO:0043236,GO:0033797,GO:0008661,GO:0008144,GO:0034784,GO:0004362,GO:0050626,GO:0035597,GO:0035870,GO:0033910,GO:0015168,GO:0036055,GO:0045154,GO:0003871,GO:0005388,GO:0004456,GO:0004730,GO:0052657,GO:0043771,GO:0001884.
